# Supplementary material for: Association between depressive symptoms and objective/subjective socioeconomic status among older adults of two regions in Myanmar
Source: PLoS One. 2021 Jan 28;16(1):e0245489. doi: 10.1371/journal.pone.0245489 (PMC7842968; doi:10.1371/journal.pone.0245489)
Supplement: S1 Questionnaires — (PDF) [file pone.0245489.s001.pdf]

Registration sheet for research project, “Healthy Ageing in Myanmar”

ကုတ် တိုင်း–မြို့နယ်–ရပ်ကွက်–ID

မေးမြန်းသည့် နေ့စွဲ ---

ဖြေဆိုသူ အမည် -------------------------------- ၁။ ကျား --- ၂။ မ ------

မွေးနေ့ --------------------------------------- ပြည့်ပြီးအသက် ---------

လိပ်စာ --

(ဖြေဆိုသူ၊ ဇနီး/ခင်ပွန်း၊ သားသမီး၊ ဆွေမျိုး ဖုန်းနံပါတ် -----------------------------------)

No.	Phone No	Who’s no
၁.		၁. ဖြေဆိုသူ ၂. ဇနီး/ခင်ပွန်း ၃. သား ၄. သမီး ၅. ဆွေမျိုး ( )
၂.		၂. ဖြေဆိုသူ ၂. ဇနီး/ခင်ပွန်း ၃. သား ၄. သမီး ၅. ဆွေမျိုး ( )
၃.		၃. ဖြေဆိုသူ ၂. ဇနီး/ခင်ပွန်း ၃. သား ၄. သမီး ၅. ဆွေမျိုး ( )
၄.		၄. ဖြေဆိုသူ ၂. ဇနီး/ခင်ပွန်း ၃. သား ၄. သမီး ၅. ဆွေမျိုး ( )
၅.		၅. ဖြေဆိုသူ ၂. ဇနီး/ခင်ပွန်း ၃. သား ၄. သမီး ၅. ဆွေမျိုး ( )

တိုင်းတာချက်များ

1. Blood pressure (လက်မောင်းတစ်ဖက်ထဲတိုင်းရန်)

1st:____________/____________mmHg 2nd:____________/____________mmHg

2-1). အရပ်: (actual measure)_________._______cm 2-2). (Demi-span)___________._______cm

Women ----- 1.35 x demi-span (cm) + 60.1 → Height ___________________ cm

Men --------- 1.40 x demi-span (cm) + 57.8 → Height ___________________ cm

3. Body weight:______.______kg

4. Body composition 1) FAT:________._______% 2) MUSCLE:________._________kg

5. Abdominal circumference:___________._______cm

6. Grip strength (please measure the same side) 1st:___________kg 2nd:___________kg

မေးခွန်းများ မေးမြန်းရာတွင် ဖြေဆိုသူနှင့် မေးမြန်းသူ တစ်ဦးချင်းသာမေးမြန်းရန်။ ဖြစ်နိုင်လျှင် ဖြေဆိုသူအနီးတွင် တခြားသူများ ရှိမနေရန်။ ရှိပါက မည်သူဟု ဖော်ပြရန်။ (တစ်ခုထက်ပို၍ ဖြေဆိုနိုင်သည်။)

၁။ အိမ်ထောင်ဖက် ၂။ သားသမီးများ ၃။ ညီအကိုမောင်နှမ ၄။ မိဘ

၅။ မြေးများနှင့် ၆။ဆွေမျိုးများ ၇။ အိမ်နီးချင်း ၈။ သူငယ်ချင်း၊ မိတ်ဆွေများနှင့်

၉။ အခြား (ဖော်ပြပါ ------------------)

အကျဉ်းချုံးမှတ်ဉာဏ်စစ်ဆေးချက်

(မေးမြန်းသူမှ မေးခွန်း ၁ မှ ၁၀ ထိမေး၍ အဖြေများ မှတ်သားထားပါ)

	မေးခွန်း	မှား	မှန်
၁။	အသက်		
၂။	အချိန် (အနီးစပ်ဆုံး အချိန်)		
၃။	လိပ်စာ (စစ်ဆေးချက်အပြီး မှတ်မိမှု ရှိ၊ မရှိ)		
၄။	လက်ရှိခုနှစ်		
၅။	လက်ရှိနေရာအမည်		
၆။	သင်သိသော လူ ၂ ယောက်ကို ရွေးချယ်ပြပါ။<br>(ဆွေမျိုး၊ မေးမြန်းသူစသည်)		
၇။	မွေးနေ့သက္ကရာဇ်		
၈။	၈၈ အရေးအခင်းဖြစ်သည့် ခုနှစ်		
၉။	မြန်မာနိုင်ငံ၏လက်ရှိသမ္မတအမည်		
၁၀။	အမှတ်စဉ် ၂၀ မှ ၁ သို့ နောက်ပြန်ရေတွက်ပါ		
	စုစုပေါင်း		

အကျဉ်းချုံးမှတ်ဉာဏ်စစ်ဆေးချက် အမှတ် ၇ မှတ် နှင့် ၇ မှတ် အထက် ရလျှင် မေးခွန်းဆက်လက်မေးမြန်းရန်။ အကျဉ်းချုံးမှတ်ဉာဏ်စစ်ဆေးချက် အမှတ် ၆ မှတ် နှင့် ၆ မှတ် အောက် ရလျှင် ဆက်လက်မေးမြန်းခြင်း မပြုရန်။

Information of surveyor

Name of surveyor: 1.______________________2.______________________

ကုတ် တိုင်း–မြို့နယ်–ရပ်ကွက်-ID

မေးခွန်း (၁)။ ပထမဦးစွာ သင်၏ရုပ်ပိုင်းဆိုင်ရာ အခြေအနေကို မေးမြန်းပါမည်။

(၁) သင်၏လက်ရှိကျန်းမာရေးအခြေအနေက ဘယ်လိုရှိပါသလဲ။

၁။ အလွန်ကောင်း ၂။ ကောင်း ၃။ သင့် ၄။ ညံ့

(၂) သင့်တစ်နေ့တာဘဝမှာ တစ်ဦးတစ်ယောက်ရဲ့ အကူအညီ (သို့) ပြုစုစောင့်ရှောက်မှုလိုအပ်ပါသလား။

၁။ မလိုပါ ၂။ လိုပါသည် (သို့သော်) မခံယူပါ ၃။ လိုပါသည်၊ ခံယူပါသည်

(၃) လွန်ခဲ့သော ၆ လ အတွင်း ဘယ်အတိုင်းအတာအထိ သင့်သွားလာလှုပ်ရှားမှု ပြသနာရှိပါသလား။ အဘယ့်ကြောင့်ဆိုသော် ကျန်းမာရေးပြသနာရှိသူဖြစ်ခဲ့လျှင် ပုံမှန်လှုပ်ရှားမှုကို ထိခိုက်နိုင်ပါသည်။

၁။ အများကြီးရှိပါသည် ၂။ အနည်းငယ်ရှိပါသည်။ ၃။ ကန့်သတ်မှု မရှိပါ။

မေးခွန်း (၂) သင့်တွင် ဘာအခက်အခဲတွေရှိပါသလဲ။

(၁) (က) အမြင်နှင့် ပတ်သက်သော အခက်အခဲရှိပါသလား။

၁။ လုံးဝအခက်အခဲမရှိပါ ၂။ အခက်အခဲအနည်းငယ်ရှိပါသည် ၃။ အခက်အခဲများစွာရှိပါသည်

၄။ ဘာမှကိုမလုပ်နိုင်ပါ။

(၁) (ခ) သင် မျက်မှန်အသုံးပြုပါသလား။

၁။ သုံးပါသည် ၂။ မသုံးပါ

(၁) (ဂ) သင် အမြင်အာရုံ ပြသနာများကြောင့် မျက်စိဆေးခန်း သွားပြဖူးပါသလား။

၁။ ပြဖူးပါသည် ၂။ မပြဖူးပါ

(၂) (က) အကြားနှင့်ပတ်သက်သော အခက်အခဲရှိပါသလား။

၁။ လုံးဝအခက်အခဲမရှိပါ ၂။ အခက်အခဲအနည်းငယ်ရှိပါသည် ၃။ အခက်အခဲများစွာရှိပါသည်

၄။ ဘာမှကိုမလုပ်နိုင်ပါ။

(၂) (ခ) သင် အကြားအကူကိရိယာအသုံးပြုရပါသလား။

၁။ သုံးပါသည် ၂။ မသုံးပါ

(၂) (ဂ) အကြားအာရုံ ပြသနာကြောင့် နား နှာခေါင်း လည်ချောင်း ဆေးခန်း သွားပြဖူးပါသလား။

၁။ ပြဖူးပါသည် ၂။ မပြဖူးပါ

(၃) (က) လမ်းလျှောက်ခြင်း၊ လှေကားတက်ခြင်း၊ ပစ္စည်းများသယ်ယူခြင်းနှင့်ပတ်သက်သော အခက်အခဲရှိပါသလား။

၁။ လုံးဝအခက်အခဲမရှိပါ ၂။ အခက်အခဲအနည်းငယ်ရှိပါသည် ၃။ အခက်အခဲများစွာရှိပါသည်

၄။ ဘာမှကိုမလုပ်နိုင်ပါ။

(၃) (ခ) လေးချောင်းထောက် (သို့) တောင်ဝှေး အသုံးပြုပါသလား။

၁။ သုံးပါသည်   ၂။ မသုံးပါ

(၃) (ဂ) လမ်းလျှောက်ရာတွင် အခက်အခဲရှိသောကြောင့် ဆရာဝန်ပြဖူးပါသလား။

၁။ ပြဖူးပါသည်   ၂။ မပြဖူးပါ

(၄) မှတ်ဉာဏ်/ အာရုံစူးစိုက်ခြင်းနှင့်ပတ်သက်သော အခက်အခဲရှိပါသလား။

၁။ လုံးဝအခက်အခဲမရှိပါ   ၂။ အခက်အခဲအနည်းငယ်ရှိပါသည်   ၃။ အခက်အခဲများစွာရှိပါသည်

၄။ ဘာမှကိုမလုပ်နိုင်ပါ။

မေးခွန်း (၃) သင်၏ နေ့စဉ်နေထိုင်သွားလာမှုနှင့်ပတ်သက်သောမေးခွန်းများ၊ နေ့စဉ်နေထိုင်မှု အခြေခံလုပ်ငန်းများ ဆောင်ရွက်ရာတွင် သူတပါးအပေါ် မှီခိုခြင်း ရှိ၊ မရှိ ဆန်းစစ်ခြင်းမေးခွန်းလွှာ

နေ့စဉ်လုပ်ငန်း	မှီခိုရခြင်းမရှိပါ	မှီခိုရပါသည်
ရေချိုးခြင်း	၁။ လက်လှမ်းမမီသောနေရာတစ်ခု တစ်လေမှလွဲပြီး ကိုယ့်ဖာသာ သန့်ရှင်းအောင်ရေချိုးနိုင်သည်။	၂။ ရေချိုးရာတွင်လည်းကောင်း၊ ရေချိုးခန်း အတွင်း ဝင်ထွက်နိုင်ရန်အတွက်လည်းကောင်း အကူအညီလိုအပ်သည်။ အခြားသူမှ ရေချိုးပေး ရပါသည်။
အဝတ်အစားဝတ်ဆင်ခြင်း	၁။ အဝတ်ထုတ်ယူခြင်း၊ ဝတ်ဆင်ခြင်း တို့အတွက် အကူအညီမလိုပါ။	၂။ အဝတ်အစားဝတ်ရာတွင် အကူအညီ လိုပါသည်။
အိမ်သာတက်ခြင်း	၁။ အိမ်သာသွားခြင်း၊ သန့်ရှင်းခြင်း အစဆုံးတို့တွင် အကူအညီမလိုပါ။	၂။ အကူအညီလိုအပ်သည်။ (သို့) အထူးစီမံ ထားသော အိမ်သာပုံစံ အသုံးပြုရသည်။
အိမ်တွင်းလှုပ်ရှားသွားလာခြင်း	၁။ အိမ်ရာဝင်ခြင်း၊ ထိုင်ခုံတွင် ထိုင်ခြင်း၊ ထခြင်းတို့တွင် လူတွဲရန် မလိုပါ။	၂။ လှုပ်ရှားသွားလာမှုတိုင်းတွင် လူတွဲရန် လိုအပ်ပါသည်။
ဆီး/ဝမ်းထိန်းနိုင်ခြင်း	၁။ လုံးဝထိန်းနိုင်ပါသည်။	၂။ တခါတရံ (သို့) လုံးဝမထိန်းနိုင်ပါ။
အစာစားခြင်း	၁။ အခြားသူပြင်ဆင်ပေးသော စားသောက်ဖွယ်ရာများကို ကိုယ့်ဖာသာ စားနိုင်ပါသည်။	၂။ တပါးသူကူညီမှသာ စားသောက်နိုင် ပါသည်။ နှာခေါင်းပိုက်မှတဆင့် စားရပါသည်။

မေးခွန်း (၄)။ အောက်ပါမေးခွန်းများသည် သင်၏ လက်ရှိနှင့်ယခင် ကျန်းမာရေးအခြေအနေနှင့်ပတ်သက်သောမေးခွန်း များဖြစ်ပါသည်။

(၁) ဆရာဝန် (သို့) သူနာပြု မှ သင့်မှာ သွေးတိုးရောဂါရှိနေပြီဟု ပြောဖူးပါသလား။

၁။ ပြောဖူးပါသည် ၂။ မပြောဖူးပါ ၃။ မသိပါ

(၂) သွေးတိုးရောဂါအတွက် ကုသမှုခံယူနေရခြင်း ရှိပါသလား။ (သွေးတိုးကျဆေးသောက်နေရခြင်း)

၁။ ရှိပါသည် (ဗမာဆေး) ၂။ ရှိပါသည် (အင်္ဂလိပ်ဆေး) ၃။ မရှိပါ ၄။ မသိပါ

(အကယ်၍ ၃၊ ၄ ဖြေခဲ့လျှင် ------ (၅) သို့ ကျော်ပါ။)

(၃) သွေးတိုးရောဂါအတွက် ညွှန်ကြားထားသောဆေးများအားလုံး(ဗမာဆေး၊ အင်္ဂလိပ်ဆေး)ကို ပုံမှန်သောက်ပါသလား။

၁။ သောက်သည် ၂။ မသောက်ပါ။

(၄) အကယ်၍ ဆေးမှန်မှန်မသောက်ဖူးဆိုလျှင် မသောက်ရသည့်အကြောင်းအရင်းကို ဖော်ပြပါ။

(တစ်ခုထက်ပို၍ဖြေဆိုနိုင်သည်။)

၁။ ကုန်ကျငွေမတတ်နိုင်၍ မသောက်ပါ။

၂။ ဆေးအလွယ်တကူဝယ်မရ၍ (ဆေးပေးခန်း၊ ဆေးဆိုင် တို့နှင့်ဝေး၍)

၃။ ဆေးသောက်ရတာ မကြိုက်၍

၄။ လိုတယ်ဟု ထင်မှသာ သောက်သည်

၅။ ဆေး၏ဘေးထွက်ဆိုးကျိုးများကို မကြိုက်၍

၆။ အနောက်တိုင်းဆေးပညာမဟုတ်သောကုထုံးများကို ပိုကြိုက်၍

၇။ ဆေးမှန်မှန်သောက်ရန် မေ့နေ၍

၈။ မသိပါ။ ၉။အခြား-------------------------------

(၅) သွေးတိုးရောဂါ၏ နောက်ဆက်တွဲဆိုးကျိုးများကို ခံစားနေရခြင်းရှိပါသလား။

၁။ မရှိပါ ၂။ ကျောက်ကပ်ရောဂါ ၃။ လေဖြတ်ခြင်း ၄။ အမြင်အာရုံချို့တဲ့ခြင်း

၅။ နှလုံးသွေးကြောရောဂါ ၆။ အခြား ၇။ မသိပါ။

(၆-၁) လွန်ခဲ့သော ၁၂ လ အတွင်း သွေးပေါင်ချိန်တိုင်းထားပါသလား။

၁။ တိုင်းသည်။ ၂။ မတိုင်းပါ။ (၂ ဖြေဆိုလျှင် (၇) သို့ ကျော်ပါ။)

(၆-၂) လွန်ခဲ့သော ၁၂ လ နှင့် နှိုင်းယှဉ်လျှင် သင်၏သွေးပေါင်ချိန်မှာ

၁။ ပိုကောင်းသည် ၂။ တူတူပဲ ၃။ ပိုဆိုးသည် ၄။ မသိပါ

(၇) သင် ယခုလက်ရှိကုသမှုခံယူနေသောရောဂါ (သို့) ဆရာဝန်ကပြောသော (သို့) ခံစားနေရသောဝေဒနာနှင့် ပတ်သက်သည်ကို ဝိုင်းပေးပါ။ (တစ်ခုထက်ပို၍ ဖြေဆိုနိုင်သည်။)

၁။ မရှိပါ	၂။ မသိပါ။
၃။ လေဖြတ် (ဥပမာ– ဦးနှောက်သွေးကြော ပေါက်/ ပိတ်)	၄။ နှလုံးရောဂါ
၅။ ဆီးချိုသွေးချို	၆။ သွေးတွင်းအဆီဓါတ်များခြင်း၊ သွေးတွင်းအဆီ ပုံမမှန်ဖြစ်ခြင်း
၇။ အသက်ရှူလမ်းကြောင်းနှင့်ဆိုင်သောရောဂါ (ဥပမာ– အဆုတ်ရောင်ရောဂါ၊ လေပြွန်ရောင် ရောဂါ)	၈။ အစာအိမ်/အူလမ်းကြောင်း၊ အသည်း/ သည်းခြေအိတ်နှင့် ဆိုင်သောရောဂါ
၉။ ကျောက်ကပ်/ဆီးကျိတ်ရောဂါ	၁၀။ ကျောရိုး/ ကြွက်သားနှင့်ဆိုင်သောရောဂါ (အရိုးပွရောဂါ/ အဆစ်ရောင်ရောဂါ)
၁၁။ ထိခိုက်ဒဏ်ရာရောဂါ (လိမ့်ကျ/ကျိုး)	၁၂ ။ ကင်ဆာရောဂါ
၁၃။ သွေးနှင့်ကိုယ်ခံအားနှင့်ဆိုင်သောရောဂါ	၁၄။ စိတ်ကျရောဂါ
၁၅။ သတိမှတ်ဉာဏ်လျော့ပါးလာသောရောဂါ (ဥပမာ– အယ်လ်ဇိုင်းမားရောဂါ)	၁၆။ တုန်တောင့်နေးရောဂါ (ပါကင်ဆန်ရောဂါ)
၁၇။ မျက်စိနှင့်ဆိုင်သောရောဂါ	၁၈ ။ နားနှင့်ဆိုင်သောရောဂါ
၁၉။ တီဘီရောဂါ	၂၀။ ခုခံအားကျဆင်းမှုကူးစက်ရောဂါ
၂၁။ ငှက်ဖျားရောဂါ	၂၂ ။ မီးယပ်နှင့်ဆိုင်သောရောဂါများ
၂၃။ အခြား (ဖော်ပြရန်---------------)	

မေးခွန်း (၅)။ အောက်ပါမေးခွန်းများသည် သင်၏ကျန်းမာရေးစောင့်ရှောက်မှုနှင့်သက်ဆိုင်သည်။

(၁) လွန်ခဲ့သော ၁၂ လ အတွင်း သင်နေမကောင်း / ဖျားနာဖူးသလား။

၁။ မဖြစ်ဖူးလျှင် (မေးခွန်း ၆ သို့သွားပါ)

၂။ ဖြစ်ဖူးတယ် ခုထိလဲ နေမကောင်း/ ဖျားနာဖြစ်နေတုန်းပဲ

၃။ ဖြစ်ဖူးတယ် ဒါပေမယ့် ခုကောင်းသွားပြီ

၄။ မမှတ်မိပါ (မေးခွန်း ၆ သို့သွားပါ)

(၂) လွန်ခဲ့သော ၁၂ လ အတွင်း သင်နေမကောင်း/ ဖျားနာတုန်းက ဆရာဝန်/ သူနာပြု/ ကျန်းမာရေးဝန်ထမ်းတစ်ဦးဦး ထံ သွားပြဖူးသလား။

၁။ အမြဲတမ်း ပြတယ်

၂။ တခါတလေမှ ပြတယ်

၃။ တခါမှ မပြဖူးပါ

၄။ မမှတ်မိပါ (၃ နှင့်၄ ကိုဖြေလျှင် ဤမေးခွန်း၏ နံပါတ် ၄ ကို ဆက်မေးရန်မလိုတော့ပါ)

(၃-၁)။ (လွန်ခဲ့သော ၁၂ လ အတွင်း သင်နေမကောင်း/ဖျားနာတုန်းက) ကျန်းမာရေးဌာနများမှာသွားပြဖို့ တုံ့ဆိုင်းနှောင့်နှေးဖူးပါသလား။

၁။ တုံ့ဆိုင်းနှောင့်နှေးဖူးတယ်

၂။ မတုံ့ဆိုင်းပါ။ (၃-၂ ကို ကျော်ပါ)

(၃-၂)။ တုံ့ဆိုင်းနှောင့်နှေးဖူးလျှင် ကျေးဇူးပြု၍ အကြောင်းအရင်းကို ဖော်ပြပါ။ အဖြေမှန်အားလုံးရွေးချယ်ပါ။

၁။ စျေးအရမ်းကြီးပါသဖြင့်

၂။ ဆေးကုဌာနမှာ ကုန်ကျတဲ့ ကုန်ကျစားရိတ်သည် အလွန်မြင့်မားလွန်းသောကြောင့်

၃။ ဆေးကုသဖို့ ဘယ်ဌာနသွားရမယ်ဆိုတာ မသိသောကြောင့်

၄။ ကျန်းမာရေးဌာနတွေက အိမ်နဲ့ အရမ်းဝေးသောကြောင့်

၅။ ဆေးကုသဖို့ လိုတယ်လို့မထင်သောကြောင့်

၆။ ဆရာဝန်ပြဖို့ အချိန်မရှိလို့

၇။ ကျန်းမာရေးအာမခံ မရှိလို့

၈။ အခြား (---)

(၄) လွန်ခဲ့သော ၁၂ လ အတွင်း မည်သည့်ကျန်းမာရေးဌာနကို သင်အသုံးပြုခဲ့ပါသလဲ။ သင့်ရဲ့ ဆေးကုသစရိတ် ဘယ်လောက်ကျပါသလဲ။ (မိမိအိတ်မှ စိုက်ထုတ်ငွေကိုဆိုလိုသည်) ကျေးဇူးပြု၍ ဆေးကုသစရိတ်တစ်ခုစီအတွက် အောက်ပါအတိုင်း နှုန်းသတ်မှတ်ပေးပါ။

(၆= မသိပါ ၅= အရမ်းစျေးကြီး ၄= စျေးကြီး ၃= သင့်တင့် ၂= သက်သာ ၁= အရမ်းသက်သာ)

ဆေးကုသမှုပေးရာနေရာ	အသုံးပြုလျှင် အမှတ်ခြစ်ပါ။ အသုံးမပြုလျှင် ကွက်လပ်ထားပါ	လွန်ခဲ့သော ၁၂ လ က စုစုပေါင်းအသုံးပြုငွေ	၆= မသိပါ။<br>၅= အရမ်းစျေးကြီး<br>၄= စျေးကြီး<br>၃= သင့်တင့်<br>၂= သက်သာ<br>၁= အရမ်းသက်သာ
**အစိုးရကျန်းမာရေးအဖွဲ့အစည်း**			
၁။ ပြည်သူ့ဆေးရုံ/အစိုးရဆေးရုံ			
၂။ ကျေးလက်ကျန်းမာရေးဌာန (RHC)			
၃။ ကျေးလက်ကျန်းမာရေးဌာနခွဲ (Subcenter)			
၄။ စေတနာ့ဝန်ထမ်းပြည်သူ့ကျန်းမာရေးဝန်ထမ်းများ (အရံသားဖွားစသည်) (Voluntary Health worker)			
၅။ အစိုးရရွေ့လျားဆေးခန်း			

၆။ မြို့နယ်ကျန်းမာရေးဌာန/ အစိုးရ မိခင်နှင့်ကလေးစောင့်ရှောက်ရေးဆေးခန်း (MCHS)			
၇။ အစိုးရတိုင်းရင်းဆေးခန်း			
၈။ အခြား			
**အစိုးရမဟုတ်သောအဖွဲ့အစည်း**			
၉။ မယ်ရီစတုတ်			
၁၀။ ကြက်ခြေနီ			
၁၁။ PSI/M (ဆန်းဆေးခန်း)			
၁၂။ မြန်မာနိုင်ငံဆရာဝန်များအသင်း			
၁၃။ အခြား			
ပြင်ပကျန်းမာရေးအဖွဲ့အစည်း			
၁၄။ ကိုယ်ပိုင်/ ပြင်ပဆေးရုံ၊ ဆေးခန်း			
၁၅။ ဆေးဆိုင်			
၁၆။ ပြင်ပဆရာဝန်			
၁၇။ ပြင်ပရွှေလျားဆေးခန်း			
၁၈။ တိုင်းရင်းဆေးခန်း			
၁၉။ အခြား			
**အခြားအရင်းအမြစ်များ**			
၂၀။ ဈေးဆိုင်			
၂၁။ တိုင်းရင်းဆေးဆရာ			
၂၂။ အခြား			
၂၃။ မသေချာပါ။			

မေးခွန်း (၆)။ အောက်ပါမေးခွန်းများသည် သင်၏ခံတွင်းအခြေအနေနှင့် ကျန်းမာရေးစစ်ဆေးမှုအခြေအနေကို မေးမြန်းခြင်းဖြစ်ပါသည်။

(၁) လက်ရှိတွင် သင့်၌ သဘာဝသွား ဘယ်နှစ်ချောင်းကျန်သလဲ။ (သဘာဝသွားဆိုသည်မှာ– ပြုပြင်၍ အစွပ်စွပ်ထားသောသွား၊ ကြီးသွား၊ အံဆုံး စသည်ဖြင့် ၃၂ ချောင်းပါဝင်သည်။)

၁။ သဘာဝသွားမရှိပါ ၂။ သဘာဝသွား ၁ မှ ၄ ချောင်းရှိ ၃။ သဘာဝသွား ၅ မှ ၉ ရှိ

၄။ သဘာဝ သွား ၁၀ မှ ၁၉ ရှိ ၅။ သဘာဝသွား ၂၀ နှင့် အထက်ရှိ

(၂) သင်သွား ဘယ်နှစ်ကြိမ်တိုက်သလဲ။

၁။ တစ်နေ့အနည်းဆုံး ၃ ကြိမ် ၂။ တစ်နေ့ ၂ ကြိမ် ၃။ တစ်နေ့တကြိမ်

၄။ တစ်နေ့တကြိမ်အောက် ၅။ တစ်ခါမှ မတိုက်ပါ။

(၃) သင်ကျန်းမာရေးစစ်ဆေးမှု လုပ်ဖူးပါသလား။ (ကျန်းမာရေးဌာနမှာ (သို့မဟုတ်) သင်ရဲ့အလုပ်ခွင်မှာ/ ဆေးတက္ကသိုလ် တစ်ခုမှာ (သို့မဟုတ်) အခြားတနေရာရာမှာ)

၁။ တစ်နှစ်အတွင်း တစ်ခါလုပ်ဖူးသည် ၂။ ၁နှစ် နှင့် ၄နှစ် အတွင်း တစ်ခါလုပ်ဖူးသည်။

၃။ လွန်ခဲ့သော ၄ နှစ်အထက် တစ်ခါလုပ်ဖူးသည် ၄။ တစ်ခါမျှမလုပ်ဖူးပါ။

မေးခွန်း (၇)။ အောက်ပါမေးခွန်းများသည် သင်၏ အစားအသောက်အမူအကျင့်များ၊ အရက်နှင့်ဆေးလိပ်သောက်ခြင်း ဆိုင်ရာအမူအကျင့်များနှင့် သက်ဆိုင်သည်။

(၁) လွန်ခဲ့သော ၆ လအတွင်း သင်အစာမာများ ဝါးရာတွင် ယခင်ကထက် ပိုခက်ခဲရှိပါသလား။

၁။ ရှိသည် ၂။ မရှိပါ

(၂) လွန်ခဲ့သော ၆ လအတွင်း လက်ဖက်ရည်/ ကော်ဖီ/ ဟင်းရည်စသော အရည်များ သောက်ရာတွင် သီးလေ့ရှိပါသလား။

၁။ ရှိသည် ၂။ မရှိပါ

(၃) လွန်ခဲ့သော ၆ လအတွင်း သင် အစားအစာများ စားသောက်ရာတွင် အခက်အခဲရှိပါသလား။ (ခံတွင်းပျက်ခြင်း၊ အရသာခံမကောင်းခြင်းပါဝင်သည်)

၁။ ရှိသည် ၂။ မရှိပါ

(၄) ပြီးခဲ့သော ၁ လ အတွင်း သင် အသား (သို့) ငါး ဘယ်နှစ်ကြိမ်စားဖြစ်သလဲ။

၁။ တစ်နေ့ ၂ ကြိမ်အထက် ၂။ တစ်နေ့တကြိမ် ၃။ တစ်ပတ် ၄-၆ ကြိမ်

၄။ တစ်ပတ် ၂-၃ ကြိမ် ၅။ တစ်ပတ်တစ်ကြိမ် ၆။ တစ်ပတ်တစ်ကြိမ်အောက်

၇။ တစ်ခါမျှမစားပါ။

(၅) ပြီးခဲ့သော ၁ လ အတွင်း သင် သစ်သီး (သို့) ဟင်းသီးဟင်းရွက် ဘယ်နှစ်ကြိမ်စားဖြစ်သလဲ။

၁။ တစ်နေ့ ၂ ကြိမ်အထက် ၂။ တစ်နေ့တကြိမ် ၃။ တစ်ပတ် ၄-၆ ကြိမ်

၄။ တစ်ပတ် ၂-၃ ကြိမ် ၅။ တစ်ပတ်တစ်ကြိမ် ၆။ တစ်ပတ်တစ်ကြိမ်အောက်

၇။ တစ်ခါမျှမစားပါ။

(၆) သင်အရက်သောက်ပါသလား။

၁။ လက်ရှိ သောက်နေသည်

၂။ လက်ရှိ မသောက်ပါ။ ဖြတ်ထားတာ ၅ နှစ် မပြည့်သေးပါ။

၃။ လက်ရှိ မသောက်ပါ။ ဖြတ်ထားတာ ၅ နှစ် ကျော်နေပြီ။

၄။ လုံးဝ မသောက်ဖူးပါ။

(၇) သင်ဆေးလိပ်/ ဆေးပေါ့လိပ် /စီးကရက် သောက်ပါသလား။

၁။ နေ့တိုင်းနီးပါးသောက်သည်။

၂။ တခါတလေသောက်သည်။ (ပြီးခဲ့သော ၁ လ အတွင်းသောက်ခဲ့သည်)

၃။ လက်ရှိမသောက်ပါ။ ဖြတ်ထားတာ ၅ နှစ် မပြည့်သေးပါ။

၄။ လက်ရှိမသောက်ပါ။ ဖြတ်ထားတာ ၅ နှစ် ကျော်နေပြီ။

၅။ လုံးဝ မသောက်ဖူးပါ။

(၈) သင် ကွမ်း/ဆေးရွက်ကြီး စားသုံးပါသလား။

၁။ နေ့တိုင်းနီးပါး သုံးဖြစ်ပါသည်။

၂။ တခါတလေသုံးသည်။ (ပြီးခဲ့သော ၁ လ အတွင်းသုံးခဲ့သည်)

၃။ လက်ရှိမသုံးပါ။ ဖြတ်ထားတာ ၅ နှစ် မပြည့်သေးပါ။

၄။ လက်ရှိမသုံးပါ။ ဖြတ်ထားတာ ၅ နှစ် ကျော်နေပြီ။

၅။ လုံးဝ မသုံးဖူးပါ။

(၉) အစားစာများကို မည်သူနှင့်အတူသင်စားပါသလဲ။ (တစ်ခုထက်မကဖြေဆိုနိုင်သည်)

၁။ တစ်ယောက်တည်း ၂။ အိမ်ထောင်ဖက်နှင့် ၃။ သားသမီးများနှင့်

၄။ မြေးများနှင့် ၅။ မိတ်ဆွေများနှင့် ၆။ အခြား (ဖော်ပြပါ---------------------)

(၁၀) အခြားသူများနှင့် သင်ဘယ်နှစ်ကြိမ် အတူစားသောက်ဖူးပါသလဲ။

၁။ နေ့တိုင်းနီးပါး (တစ်ပတ် ၄-၇ ကြိမ်) ၂။ တစ်ခါတစ်လေ ၁-၃ကြိမ်

၃။ အနည်းငယ် (တစ်လ ၁-၃ ကြိမ်) ၄။ နည်းပါး (တစ်လတစ်ကြိမ်အောက်)

၅။ မရှိသလောက်ရှား (တစ်နှစ်မှာတစ်ကြိမ်အောက်)

မေးခွန်း (၈)။ အောက်ပါမေးခွန်းများသည် သင်၏ နေ့စဉ်ဘဝလုပ်ဆောင်မှုများနှင့် သက်ဆိုင်သည်။

(၁) သင် အပြင်ဘယ်နှစ်ကြိမ်လောက် သွားဖြစ်သလဲ။ (အပြင်ဆိုသည်မှာ အိမ်ပြင်ပ၊ လယ်ယာ၊ အိမ်နီးချင်းအိမ်၊ ဈေးဝယ်ထွက်၊ ဆေးရုံ၊ ဘုရား၊ ဘုန်းကြီးကျောင်း၊ ဘုရားကျောင်းများ ပါဝင်သည်။)

၁။ တစ်ပတ် ၄ ကြိမ် နှင့်အထက် ၂။ တစ်ပတ် ၂-၃ ကြိမ် ၃။ တစ်ပတ်တစ်ကြိမ်

၄။ တစ်လ ၁-၃ ကြိမ် ၅။ တစ်နှစ်တစ်ကြိမ်အောက် ၆။ မသွားသလောက်နည်း၊ မသွားဖြစ်ပါ။

(၂) သင် အပြင်သွားတဲ့အခါ ဘာကိုအသုံးပြုပါသလဲ။ (တစ်ခုထက်ပို၍ဖြေဆိုနိုင်သည်)

၁။ ခြေလျင် (ခြေလျင်ဟုဖြေဆိုလျှင် အမှတ်စဉ် ၂ မှ ၁၆ ထိ အသုံးပြုရန်မလိုပါ)

၂။ စက်ဘီး　　၃။ ဆိုင်ကယ်　　၄။ မော်တော်ကား (ကိုယ်တိုင်မောင်း၍)

၅။ မော်တော်ကား (ကားမောင်းသူဖြင့်)　　၆။ ရထား　　၇။ အများသုံးယာဉ် (ဘတ်စ်ကား)

၈။ လှေ/သမ္ဗာန်/မော်တော်ဘုတ် (ကိုယ်တိုင်မောင်း၍)

၉။ လှေ/သမ္ဗာန်/မော်တော်ဘုတ် (မောင်းသူဖြင့်)

၁၀။ တွန်းလှည်း　　၁၁။ စက်တပ်တွန်းလှည်း

၁၂။ မသန်စွမ်းသူများ၊ သက်ကြီးရွယ်အိုများအတွက် တီထွင်ထားသော လမ်းလျှောက်ကိရိယာ

၁၃။ အငှားယာဉ် (တက်ကစီ)　　၁၄။ ဆိုက်ကား/သုံးဘီးဆိုင်ကယ်

၁၅။ နောက်တွဲယာဉ်　　၁၆။ အခြား (ဖော်ပြပါ-------------------)

(၃) လွန်ခဲ့သော ၁ နှစ် အတွင်း သင်လိမ့်ကျ/ချော်လဲဖူးပါသလား။ (အကြိမ်ရေ မေးလို့ရလျှင် မေးလာပါ)

၁။ အကြိမ်များစွာ　　၂။ ၁ကြိမ်　　၃။ တစ်ခါမှ မဖြစ်ဖူးပါ

(၄) သင် လိမ့်ကျ/ချော်လဲမှာ အရမ်းစိုးရိမ်နေပါသလား။

၁။ အရမ်းကို စိုးရိမ်နေသည်　　၂။ အတိုင်းအတာတစ်ခုအထိ

၃။ အနည်းငယ်မျှသာ　　၄။ မစိုးရိမ်ပါ

(၅) သင် လှေကားတက်ရာတွင် လက်ရန်း (သို့) နံရံကို မကိုင်ဘဲတက်နိုင်ပါသလား။

၁။ အမြဲတက်နိုင်သည်။　　၂။ တခါတလေ မကိုင်ဘဲ တက်နိုင်သည်။　　၃။ မတက်နိုင်ပါ။

(၆) သင် ထိုင်ခုံပေါ်မှ ထလျှင်/ဆင်းလျှင် ဘယ်အရာဝတ္ထုကိုမျှမကိုင်ဘဲ လုပ်နိုင်ပါသလား။

၁။ အမြဲလုပ်နိုင်သည်။　　၂။ တခါတလေ မကိုင်ဘဲ လုပ်နိုင်သည်။　　၃။ မလုပ်နိုင်ပါ။

(၇) သင်တစ်နေ့လျှင် (တစ်နေ့လုံးပေါင်း) ခန့်မှန်းခြေ အချိန်မည်မျှ လမ်းလျှောက်ဖြစ်ပါသလဲ။

၁။ လမ်းမလျှောက်နိုင်ပါ　　၂။ နာရီဝက်အောက်　　၃။ နာရီဝက် မှ ၁ နာရီ ခန့်

၄။ ၁ နာရီ မှ ၁ နာရီခွဲခန့်　　၅။ တစ်နာရီခွဲနှင့်အထက်

မေးခွန်း (၉)။ အောက်ပါမေးခွန်းများသည် သင့်၏နေ့စဉ်ဘဝနှင့် သက်ဆိုင်သည်။ (အသင့်တော်ဆုံးအဖြေကိုသာ ရွေးချယ်ပါ။)

(၁)	သင်တစ်ယောက်တည်း ရထား (သို့) ဘတ်စ်ကားဖြင့် သွားသလား။	၁။ အမြဲသွားသည်။<br>၂။ သွားသည်၊ အမြဲမဟုတ်ပါ။<br>၃။ လုံးဝမသွားနိုင်ပါ။

(၂)	သင့်ရဲ့နေ့စဉ်အသုံးအဆောင်များလိုအပ်လျှင် ဈေးဝယ်ထွက်သလား။	၁။ အမြဲသွားသည်။<br>၂။ သွားသည်၊ အမြဲမဟုတ်ပါ။<br>၃။ လုံးဝမသွားနိုင်ပါ။
(၃)	သင်ကိုယ်တိုင် ချက်ပြုတ်သလား။	၁။ အမြဲလုပ်သည်။<br>၂။ လုပ်သည်၊ အမြဲမဟုတ်ပါ။<br>၃။ လုံးဝမလုပ်နိုင်ပါ။
(၄)	သင် သတင်းစာ ဖတ်နိုင်သလား။	၁။ ဖတ်နိုင်သည်။<br>၂။ မဖတ်နိုင်ပါ။<br>၃။ စာမတတ်ပါ။
(၅)	သင် စာအုပ်များ(သို့) မဂ္ဂဇင်းများ ဖတ်နိုင်သလား။	၁။ ဖတ်နိုင်သည်။<br>၂။ မဖတ်နိုင်ပါ။<br>၃။ စာမတတ်ပါ။
(၆)	သင် ကျန်းမာရေးနှင့်ပတ်သက်သော စာစောင်များ (သို့) တီဗီအစီအစဉ်များကိုစိတ်ဝင်စားပါသလား။	၁။ စိတ်ဝင်စားပါသည်။<br>၂။ စိတ်မဝင်စားပါ။
(၇)	သင့်ရဲမိတ်ဆွေ/ ဆွေမျိုးများအိမ်သို့ အလည်သွားပါသလား။	၁။ သွားပါသည်။<br>၂။ မသွားပါ။
(၈)	သင့်မိသားစုဝင် (သို့) မိတ်ဆွေများကို အကြံဉာဏ် ပေးဖူးပါသလား။	၁။ ပေးဖူးပါသည်။<br>၂။ မပေးဖူးပါ။
(၉)	သင် လိမ့်ကျ/ ချော်လဲ/ ဖျားနာသောသူထံ လူနာသတင်းမေး သွားဖူးပါသလား။	၁။ သွားပါသည်။<br>၂။ မသွားပါ။
(၁၀)	လူငယ်များနှင့် သင်စကားစမြည် ပြောလေ့ရှိပါသလား။	၁။ ပြောလေ့ရှိပါသည်။<br>၂။ ပြောလေ့မရှိပါ။
(၁၁)	သင် ၁၅မိနစ်ခန့် မရပ်မနားလမ်းလျှောက်နိုင်ပါသလား။	၁။ အမြဲလျှောက်နိုင်သည်။<br>၂။ လျှောက်နိုင်သည်၊ အမြဲမဟုတ်ပါ။<br>၃။ လုံးဝမလျှောက်နိုင်ပါ။
(၁၂)	သင့်အနီးရှိ လူများသည် သင်သတိမေ့တာကို သတိထားမိပါသလား။ (ဥပမာ မေးပြီးသား အကြောင်းအရာကို ပြန်မေးတာမျိုး)	၁။ သတိထားမိသည်။<br>၂။ သတိမထားမိပါ။
(၁၃)	ဒီနေ့ ဘာနေ့လဲ (ရက်စွဲ) ကို တခါတလေ သင်မေ့နေပါသလား။	၁။ မေ့သည်။<br>၂။ မမေ့ပါ။
(၁၄)	အခုရက်ပိုင်းအတွင်း သင်သတိမေ့တတ်တယ်လို့ ထင်ပါသလား။	၁။ ထင်သည်။<br>၂။ မထင်ပါ။

မေးခွန်း (၁၀)။ အောက်ပါ မေးခွန်းများသည် သင်၏ဝါသနာနှင့်သက်ဆိုင်သော လုပ်ဆောင်မှုများဖြစ်သည်။

(၁) သင်ဝါသနာပါသော ကိစ္စများရှိပါသလား။

၁။ ရှိသည်　　　　၂။ မရှိပါ ။　　　　(၂ ဖြေရင် မေးခွန်း ၁၁ ကို ဆက်သွားပါ)

(၂) သင့်၏ ဝါသနာတွေက ဘာလဲ။ သင်၏ ဝါသနာများကို ပြောပြပါ။

မေးခွန်း (၁၁)။ အောက်ပါမေးခွန်းများသည် ယခုလက်ရှိ သင် ပါဝင်လုပ်ဆောင်နေသော အဖွဲ့အစည်း၊ အလုပ်များနှင့် သက်ဆိုင်သည်။

(၁) အောက်ဖော်ပြပါ အဖွဲ့အစည်းများတွင် သင်ဘယ်နှစ်ကြိမ် ပါဝင်လုပ်ရှားဖူးပါသလဲ။

၁။ ဘာသာရေးအဖွဲ့အစည်း

၁။ တပတ် ၄ ကြိမ်နှင့် အထက်　　၂။ တပတ် ၂–၃ ကြိမ်　　၃။ တပတ်တကြိမ်

၄။ တလ ၁–၃ ကြိမ်　　၅။ တနှစ် မှာ အနည်းငယ်　　၆။ မပါပါ

၂။ ပရဟိတအဖွဲ့အစည်း (ဤအဖွဲ့အစည်းသည် သင့်ဆန္ဒအလျောက်ပါဝင်သော ငွေကြေးတစုံတရာမပေးရသည့် အဖွဲ့ဖြစ်သည်။ ဥပမာ– ကြက်ခြေနီ၊ ငွေကြေးလှူဒါန်းသောအဖွဲ့၊ နာရေးကူညီမှုအသင်း စသည်)

၁။ တပတ် ၄ ကြိမ်နှင့် အထက်　　၂။ တပတ် ၂–၃ ကြိမ်　　၃။ တပတ်တကြိမ်

၄။ တလ ၁–၃ ကြိမ်　　၅။ တနှစ် မှာ အနည်းငယ်　　၆။ မပါပါ

၃။ အားကစားအဖွဲ့ (သို့) ကလပ်အသင်း

၁။ တပတ် ၄ ကြိမ်နှင့် အထက်　　၂။ တပတ် ၂–၃ ကြိမ်　　၃။ တပတ်တကြိမ်

၄။ တလ ၁–၃ ကြိမ်　　၅။ တနှစ် မှာ အနည်းငယ်　　၆။ မပါပါ

၄။ ဝါသနာတူအဖွဲ့

၁။ တပတ် ၄ ကြိမ်နှင့် အထက်　　၂။ တပတ် ၂–၃ ကြိမ်　　၃။ တပတ်တကြိမ်

၄။ တလ ၁–၃ ကြိမ်　　၅။ တနှစ် မှာ အနည်းငယ်　　၆။ မပါပါ

၅။ လူထုတွေ့ဆုံပွဲများ (ဥပမာ– မြို့နယ်အသင်းတွေ့ဆုံပွဲများ၊ ဆရာကန်တော့ပွဲများ)

၁။ တပတ် ၄ ကြိမ်နှင့် အထက်　　၂။ တပတ် ၂–၃ ကြိမ်　　၃။ တပတ်တကြိမ်

၄။ တလ ၁–၃ ကြိမ်　　၅။ တနှစ် မှာ အနည်းငယ်　　၆။ မပါပါ

၆။ နိုင်ငံရေးလှုပ်ရှားမှု/ တွေ့ဆုံပွဲများ

၁။ တပတ် ၄ ကြိမ်နှင့် အထက်　　၂။ တပတ် ၂–၃ ကြိမ်　　၃။ တပတ်တကြိမ်

၄။ တလ ၁–၃ ကြိမ်　　၅။ တနှစ် မှာ အနည်းငယ်　　၆။ မပါပါ

(၂) အထက်ပါ အဖွဲ့အစည်းများထဲမှ (၁–၆) သင်အများဆုံးပါဝင်ဖြစ်သောအဖွဲ့ကို ဖော်ပြပါ။

၁။ ဘာသာရေးအဖွဲ့အစည်း　　၂။ ပရဟိတအဖွဲ့အစည်း　　၃။ အားကစားအဖွဲ့ (သို့) ကလပ်အသင်း

၄။ ဝါသနာတူအဖွဲ့　　၅။ လူထုတွေ့ဆုံပွဲများ　　၆။ နိုင်ငံရေးလှုပ်ရှားမှု/ တွေ့ဆုံပွဲများ

မေးခွန်း (၁၂)။ မေးခွန်း ၁၁-၂ တွင် ရွေးချယ်ခဲ့သော သင်အများဆုံးပါဝင်ဖြစ်သောအဖွဲ့အတွက် အောက်ပါအဖြေများ ထဲမှ အသင့်တော်ဆုံးကို ရွေးချယ်ပါ။

(၁) ကျား/မ အချိုး

၁။ ယောက်ျား (သို့) မိန်းမသီးသန့်သာ ၂။ ယောက်ျားဦးရေပိုများ

၃။ မိန်းမဦးရေပိုများ ၄။ ယောက်ျား/မိန်းမအချိုးတူ

(၂) နေရာ

၁။ ရပ်ကွက်တူ/ကျေးရွာတူသူများသာ ၂။ ရပ်ကွက်/ကျေးရွာမတူသူများ

(၃) အသက်အုပ်စု

၁။ အများစုမှာ ရွယ်တူများ ၂။ အသက်အရွယ်မရွေး

(၄) လူမှုအဆင့်အတန်း

၁။ လူမှုအဆင့်အတန်းမြင့်မားသူများ (ဥပမာ- ရာထူး/အာဏာရှိပုဂ္ဂိုလ်နိုင်ငံရေးသမား၊ မြို့တော်ဝန်အဖွဲ့၊ ကုန်သည်၊ ကုမ္ပဏီပိုင်ရှင်၊ ဆရာဝန်၊ ရှေ့နေ စသည်)

၂။ သာမာန်လူမှုအဆင့်အတန်းရှိသူများ

(၅) လူမျိုးဖွဲ့စည်းမှု

၁။ လူမျိုးမရွေး ပါဝင်သည်

၂။ လူမျိုးတူသူများသာ ပါဝင်သည်

မေးခွန်း (၁၃)။ အောက်ပါမေးခွန်းများသည် သင်နှင့်သင့်မိတ်ဆွေများ၏ ပေါင်းသင်းဆက်ဆံရေးနှင့် သက်ဆိုင်သည်။

(၁) သင့်မိတ်ဆွေများ၊ အသိများနဲ့ ဘယ်နှစ်ကြိမ်တွေ့ဖြစ်သလဲ။

၁။ တစ်ပတ် ၄ ကြိမ် နှင့်အထက် ၂။ တစ်ပတ် ၂-၃ ကြိမ် ၃။ ၁ပတ်တကြိမ်

၄။ တစ်လ ၁-၃ ကြိမ် ၅။ တစ်နှစ်မှာအကြိမ်အနည်းငယ် ၆။ မတွေ့ဖြစ်ပါ

(၂) ပြီးခဲ့သောတစ်လအတွင်းသင့်သူငယ်ချင်း/ မိတ်ဆွေအသိအကျွမ်း ဘယ်နှစ်ယောက်နှင့် တွေ့ဆုံဖြစ်ပါသလဲ။ လူတူလျှင် တစ်ကြိမ်သာရေတွက်ပါ။

၁။ မရှိပါ ၂။ ၁-၂ ၃။ ၃-၅ ၄။ ၆-၉ ၅။ ၁၀နှင့်အထက်

(၃) သင် မကြာခဏတွေ့ဆုံဖြစ်တာ ဘယ်သူတွေလဲ။ (တစ်ခုထက်ပို၍ ဖြေဆိုနိုင်သည်)

၁။ အိမ်နီးချင်းမိတ်ဆွေများ ၂။ ငယ်သူငယ်ချင်း

၃။ ကျောင်းနေဘက်သူငယ်ချင်း ၄။ လုပ်ဖော်ကိုင်ဖက်များ

၅။ ဝါသနာတူမိတ်ဆွေ ၆။ ပါဝင်ထားသော အသင်းအဖွဲ့တူသူငယ်ချင်း

၇။ အခြား (ဖော်ပြပါ----------------) ၈။ မိတ်ဆွေသူငယ်ချင်းမရှိပါ။

မေးခွန်း (၁၄)။ သင်၏ပေါင်းသင်းဆက်ဆံရေးနှင့် ပတ်သက်သောမေးခွန်း

(၁) သင်နှင့်အမြဲတမ်းဆက်ဆံနေသောသူများအနက် အောက်ပါနေရာများတွင် နေထိုင်သောသူများရှိပါသလား။

အနီးစပ်ဆုံးနေထိုင်သောနေရာကို ဖော်ပြပါ။ တစ်ဦးချင်းကို ဖြေဆိုပေးပါ။ (တစ်ခုထက်ပို၍ဖြေဆိုနိုင်ပါသည်)

	မိသားစုဝင်များ မိဘ/ မြေးများ/ ဇနီး/ ခင်ပွန်း/ သားသမီး/ မောင်နှမများ	အခြားသော ဆွေမျိုးများ	အလုပ်နှင့် ပတ်သက်သော မိတ်ဆွေများ/ လုပ်ဖော်ကိုင် ဖက်များ	အခြား သူငယ်ချင်း များ	မည်သူမှ မရှိပါ
၁။ ၁၀မိနစ်ခန့် လျှောက်သွားရသော နေရာတွင်နေသည်	၁	၂	၃	၄	၅
၂။ တမြို့တည်း/တရပ်ကွက်တည်း ၁၀မိနစ်အထက် လျှောက် သွားရသောနေရာတွင် နေသည်	၁	၂	၃	၄	၅
၃။ တမြို့တည်းမဟုတ်သော်လည်း ပြည်နယ်/တိုင်း၊ တခုတည်းတွင် နေသည်	၁	၂	၃	၄	၅
၄။ ပြည်နယ်တိုင်းမတူပါ	၁	၂	၃	၄	၅
၅။ နိုင်ငံခြားတွင်နေသည်	၁	၂	၃	၄	၅

(၂) ပုံမှန်အားဖြင့်ဆိုလျှင် လူအများစုက ယုံကြည်လို့ရတယ်လို့ သင်ထင်ပါသလား။

၁။ ထင်ပါသည် ၂။ မထင်ပါ ၃။ အခြေအနေပေါ်မူတည်ပါသည်။

မေးခွန်း (၁၅)။ အောက်ပါမေးခွန်းများသည် သင်နှင့်သင့်အနားရှိသူ၏ အပြန်အလှန်အမှီသဟဲပြုမှုနှင့် သက်ဆိုင်သည်။

(၁) သင့်မှာ သင့်ရဲ့ စိုးရိမ်ပူပန်မှု၊ တိုင်တန်းမှုများကို မည်သူက နားထောင်ပေးပါသနည်း။

(တခုထက်ပို၍ဖြေဆိုနိုင်ပါသည်)

၁။ အိမ်ထောင်ဖက် ၂။ အတူနေသားသမီး ၃။ အတူမနေသားသမီး

၄။ ညီအကိုမောင်နှမ/ဆွေမျိုး/မိဘ၊ မြေး ၅။ အိမ်နီးချင်း ၆။ မိတ်ဆွေသူငယ်ချင်း

၇။ အခြား (ဖော်ပြပါ-------) ၈။ ထိုကဲ့သို့လူများမရှိပါ။

(၂) သင်တစုံတယောက်ရဲ့စိုးရိမ်ပူပန်မှု၊ တိုင်တန်းမှုများကိုနားထောင်ပေးပါသလား။ (တခုထက်ပို၍ဖြေဆိုနိုင်သည်)

၁။ အိမ်ထောင်ဖက် ၂။ အတူနေသားသမီး ၃။ အတူမနေသားသမီး

၄။ ညီအကိုမောင်နှမ/ဆွေမျိုး/မိဘ၊ မြေး ၅။ အိမ်နီးချင်း ၆။ မိတ်ဆွေသူငယ်ချင်း

၇။ အခြား (ဖော်ပြပါ-------) ၈။ ထိုကဲ့သို့လူများမရှိပါ။

(၃) သင်နေမကောင်းတဲ့အခါ/အိပ်ရာထဲလဲနေတဲ့အခါ မည်သူက ကြည့်ရှုစောင့်ရှောက်မှုပေးပါသလဲ။

(တခုထက်ပို၍ဖြေဆိုနိုင်သည်)

၁။ အိမ်ထောင်ဖက် ၂။ အတူနေသားသမီး ၃။ အတူမနေသားသမီး

၄။ ညီအကိုမောင်နှမ/ဆွေမျိုး/မိဘ၊ မြေး ၅။ အိမ်နီးချင်း ၆။ မိတ်ဆွေသူငယ်ချင်း

၇။ အခြား (ဖော်ပြပါ-------) ၈။ ထိုကဲ့သို့လူများမရှိပါ။

(၄) တစုံတယောက်နေမကောင်းတဲ့အခါ၊ အိပ်ရာထဲလဲနေတဲ့အခါ သင်ကြည့်ရှုစောင့်ရှောက်မှုပေးဖူးပါသလား။

(တခုထက်ပို၍ဖြေဆိုနိုင်သည်)

၁။ အိမ်ထောင်ဖက် ၂။ အတူနေသားသမီး ၃။ အတူမနေသားသမီး

၄။ ညီအကိုမောင်နှမ/ဆွေမျိုး/မိဘ၊ မြေး ၅။ အိမ်နီးချင်း ၆။ မိတ်ဆွေသူငယ်ချင်း

၇။ အခြား (ဖော်ပြပါ-------) ၈။ ထိုကဲ့သို့လူများမရှိပါ။

(၅) သင် ဒုက္ခရောက်တဲ့အခါ တစုံတယောက်ကို တိုင်ပင်ဆွေးနွေးဖူးပါသလား။ (တခုထက်ပို၍ဖြေဆိုနိုင်သည်)

၁။ အတူနေမိသားစုဝင်

၂။ အတူမနေသားသမီးများ

၃။ မွေးချင်း/ဆွေမျိုး/မိဘ/မြေး

၄။ အိမ်နီးချင်း/သူငယ်ချင်းမိတ်ဆွေ

၅။ လူထုအသင်းအဖွဲ့ /အိမ်နီးချင်းအသင်းအဖွဲ့

၆။ လူမှုကူညီရေးအသင်း/ပြန်တမ်းဝင်အဖွဲ့ (ဥပမာ စစ်မှုထမ်းဟောင်းအဖွဲ့)

၇။ အထွေထွေရောဂါကုဆရာဝန်၊ သွားဆရာဝန်၊ သူနာပြု

၈။ ဒေသဆိုင်ရာထောက်ပံ့ရေးဌာန/ရုံးအဖွဲ့

၉။အခြား (ဖော်ပြပါ---) ၁၀။ထိုကဲ့သို့သူမျိုးမရှိပါ။

မေးခွန်း (၁၆) ။ အောက်ပါမေးခွန်းများသည် သင်နေထိုင်သောနေရာဒေသနှင့် သက်ဆိုင်သည်။

(၁) သင်နေထိုင်ရာဒေသရှိလူများသည် ယေဘုယျအားဖြင့် ယုံကြည်စိတ်ချရသည်ဟု ထင်ပါသလား။

၁။ အလွန်ယုံကြည်ရသည် ၂။ အတန်အသင့်ယုံကြည်ရသည်

၃။ မပြောတတ်ပါ ၄။ သိပ်မယုံကြည်ရပါ ၅။ လုံးဝမယုံကြည်ရပါ။

(၂) သင့်အနီးဝန်းကျင်တွင်နေထိုင်သူများက နေရာတော်တော်များများတွင် တခြားသူများကို ကူညီချင်စိတ်ရှိသည်ဟု သင်ထင်ပါသလား။

၁။ များစွာကူညီသည် ၂။ အသင့်အတင့် ၃။ မပြောတတ်ပါ ၄။ အနည်းငယ် ၅။ မထင်ပါ

(၃) သင် နေထိုင်သောနေရာကို နှစ်သက်ပါသလား။

၁။ များစွာနှစ်သက်သည်　　၂။ အသင့်အတင့်　　၃။ မပြောတက်ပါ　　၄။ အနည်းငယ်　　၅။ မနှစ်သက်ပါ

(၄) သင့်ပတ်ဝန်းကျင်တွင် ရာဇဝတ်မှုများနှင့်ပတ်သက်၍ သင် စိတ်ပူပန်နေရပါသလား။

၁။ အရမ်း　　၂။ အသင့်အတင့်　　၃။ မပြောတတ်ပါ　　၄။ သိပ်မပူရပါ　　၅။ လုံးဝမပူရပါ။

(၅) သင်နှင့် အိမ်နီးနားချင်းတို့၏ဆက်ဆံမှုအများဆုံးပုံစံကို ဖော်ပြပါ။

၁။ အပြန်အလှန်ကူညီခြင်း၊ နေ့စဉ်သုံးပစ္စည်းများငှားရမ်းခြင်း၊ နေ့စဉ်နေထိုင်မှုတွင်ကူညီခြင်း

၂။ နေ့တိုင်းစကားစမြည်ပြောခြင်း

၃။ နှုတ်ဆက်ရုံသာ နှုတ်ဆက်ခြင်း

၄။ မည်သည့်ဆက်ဆံရေးမှမရှိပါ

(၆) သင်၏အိမ်အနီးအနား (ခြေလျှင်သွား၍ရသော/ ရပ်ကွက်ထဲ) တွင် အောက်ပါနေရာများ ရှိပါသလား။

၁) ပန်းခြံ (သို့) လမ်းလျှောက်လေ့ကျင့်ခန်းလုပ်ရန်နေရာ

၁။ ရှိပါသည်　　၂။ မရှိပါ　　၃။ ရှိမရှိ မသိပါ

၂) သား၊ ငါးနှင့်ဟင်းသီးဟင်းရွက် ဝယ်ယူရရှိနိုင်သောနေရာ (ဈေး/ ဈေးဝယ်စင်တာ)

၁။ ရှိပါသည်　　၂။ မရှိပါ　　၃။ ရှိမရှိ မသိပါ

မေးခွန်း (၁၇)။ အောက်ပါမေးခွန်းများထဲမှ အဖြေမှန်ကို ဝိုင်းပါ။ (မသိဟုဖြေလျှင် ဝိုင်းရန်မလိုပါ)

(၁)	သင်၏လက်ရှိဘဝကိုကျေနပ်မှုရှိပါသလား။	ရှိ	မရှိ
(၂)	တစ်ချိန်ချိန်တွင် ဆက်လက်ရှင်သန်လိုစိတ်မရှိဟု ခံစားဖူးခြင်းရှိပါသလား။	ရှိ	မရှိ
(၃)	နေ့စဉ်လှုပ်ရှားရုန်းကန်နေရသော အင်အားလျော့နည်းသွားခြင်း (သို့) သင့်ပတ်ဝန်းကျင်အပေါ်ကို စိတ်ဝင်စားမှု လျော့နည်းသွားခြင်းရှိပါသလား။	ရှိ	မရှိ
(၄)	ဘဝဟာ အဓိပ္ပါယ်မဲ့နေသည်ဟု ခံစားမိခြင်းရှိပါသလား။	ရှိ	မရှိ
(၅)	မကြာခဏ စိတ်ပျက်ငြီးငွေ့တတ်ခြင်း ရှိပါသလား။	ရှိ	မရှိ
(၆)	အများအားဖြင့် အဆင်ပြေနေသည်ဟု ခံစားမိခြင်း ရှိပါသလား။	ရှိ	မရှိ
(၇)	မကောင်းသောအခြင်းအရာတစ်ခုဖြစ်တော့မည်ဟု ခံစားရခြင်း ရှိပါသလား။	ရှိ	မရှိ
(၈)	သင့်ကိုယ်သင် ကံကောင်းသည်ဟု ထင်ပါသလား။	ရှိ	မရှိ
(၉)	မကြာခဏအကူအညီမဲ့နေသလို ခံစားမိခြင်း ရှိပါသလား။	ရှိ	မရှိ
(၁၀)	အပြင်ထွက်လည်ပတ်ခြင်းထက် အိမ်မှာနေရခြင်းကိုပို၍ ကြိုက်နှစ်သက်ပါသလား။	ရှိ	မရှိ
(၁၁)	သင်သည် အခြားသူများထက် ပိုမေ့တတ်သည်ဟု ထင်ပါသလား။	ရှိ	မရှိ
(၁၂)	ဘဝဟာ နှစ်သက်ပျော်ရွှင်ဖွယ်ကောင်းတယ်လို့ ထင်ပါသလား။	ရှိ	မရှိ

(၁၃) သင့်ကိုယ်သင် စွမ်းအင်များ ပြည့်ဝနေသည်ဟု ထင်ပါသလား။ ရှိ မရှိ

(၁၄) သင့်ဘဝမှာ မျှော်လင့်ချက်မရှိဟုထင်ပါသလား။ ရှိ မရှိ

(၁၅) အခြားသူများက သင့်ထက် ငွေရေးကြေးရေးပိုအဆင်ပြေသည်ဟု ထင်ပါသလား။ ရှိ မရှိ

မေးခွန်း (၁၈)။ သင်၏ ကိုယ်ရေးကိုယ်တာနှင့်သက်ဆိုင်သောမေးခွန်းများ

(၁) လိင် ၁။ ကျား ၂။ မ

(၂) အသက် ------------- နှစ် (ပြည့်ပြီးအသက်)

(၃) လူမျိုး

၁။ ဗမာ ၂။ ကချင် ၃။ ကယား ၄။ ကရင် ၅။ ချင်း ၆။ မွန် ၇။ ရခိုင်

၈။ ရှမ်း ၉။ မြန်မာနိုင်ငံရှိအခြားတိုင်းရင်းသားမျိုးနွယ်စုများ ၁၀။ အခြား ၁၁။ မဖြေလိုပါ

(၄) ပရဟိတလုပ်ငန်းများတွင် ပါဝင်ဆောင်ရွက်ဖူးပါသလား။ (တစ်ဦးချင်းလုပ်ခြင်းကိုမဆိုလိုပါ)

(အလှူလုပ်ခြင်း၊ လူငယ်များနှင့်တွေ့ဆုံခြင်း၊ သင့်ပတ်ဝန်းကျင်အကျိုးရှိရေး မည်မျှဆောင်ရွက်ခဲ့ပါသနည်း။)

၁။ တစ်ပတ် ၄ ကြိမ် နှင့်အထက် ၂။ တစ်ပတ် ၂ ကြိမ် မှ ၃ ကြိမ် ၃။ တစ်ပတ်တကြိမ်

၄။ တစ်လ ၁ ကြိမ် မှ ၃ ကြိမ် ၅။ တစ်နှစ်မှာ အကြိမ်အနည်းငယ် ၆။ မလုပ်ဖြစ်ပါ။

(၅) မကြာခဏ အလှူအတန်းများ ပြုလုပ်လေ့ရှိပါသလား။

၁။ တစ်ပတ် ၄ ကြိမ် နှင့်အထက် ၂။ တစ်ပတ် ၂ ကြိမ် မှ ၃ ကြိမ် ၃။ တစ်ပတ်တကြိမ်

၄။ တစ်လ ၁ ကြိမ် မှ ၃ ကြိမ် ၅။ တစ်နှစ်မှာ အကြိမ်အနည်းငယ် ၆။ မလှူဖြစ်ပါ။

(၆) ကိုးကွယ်ကွယ်သည့်ဘာသာ

၁။ ဗုဒ္ဓဘာသာ ၂။ အစ္စလာမ်ဘာသာ ၃။ ခရစ်ယာန် ၄။ ဟိန္ဒူ

၅။ အခြား (ဖော်ပြပါ -------------------) ၆။ ကိုးကွယ်သည့်ဘာသာမရှိပါ။

(၇) ဘုန်းကြီးကျောင်း/ဘုရား၊ ဗလီ၊ ခရစ်ယာန်ဘုရားကျောင်း ဘယ်နှစ်ကြိမ်ခန့် သွားလေ့ရှိပါသနည်း။

၁။ တစ်ပတ် ၄ ကြိမ် နှင့်အထက် ၂။ တစ်ပတ် ၂ ကြိမ် မှ ၃ ကြိမ် ၃။ တစ်ပတ်တကြိမ်

၄။ တစ်လ ၁ ကြိမ် မှ ၃ ကြိမ် ၅။ တစ်နှစ်မှာ အကြိမ်အနည်းငယ် ၆။ မသွားဖြစ်ပါ။

(၈) အိမ်တွင်တရားထိုင်ခြင်း၊ ဆုတောင်းခြင်း၊ ဘုရားရှိခိုးခြင်း ဘယ်နှစ်ကြိမ် လုပ်လေ့ရှိပါသလဲ။

၁။ တစ်ပတ် ၄ ကြိမ် နှင့်အထက် ၂။ တစ်ပတ် ၂ ကြိမ် မှ ၃ ကြိမ် ၃။ တစ်ပတ်တကြိမ်

၄။ တစ်လ ၁ ကြိမ် မှ ၃ ကြိမ် ၅။ တစ်နှစ်မှာ အကြိမ်အနည်းငယ် ၆။ မလုပ်ဖြစ်ပါ။

(၉) သင့်ဘဝတွင် ဘာသာရေးသည် မည်မျှအရေးပါသနည်း။

၁။ လုံးဝအရေးမပါ ၂။ အနည်းငယ်အရေးပါ ၃။ အသင့်အတင့် ၄။ အရမ်းအရေးပါ

၅။ မသိပါ ၆။ မဖြေဆိုလိုပါ

(၁၀) ယခု သင်မည်မျှ ပျော်ရွှင်သနည်း။ (လုံး၀မပျော်ရွှင် ၀ မှ အရမ်းပျော်ရွှင် ၁၀ ထိ ခန့်မှန်းပေးပါ)

မပျော်ရွှင် ၀--၁--၂--၃--၄--၅--၆--၇--၈--၉--၁၀ ပျော်ရွှင်

(၁၁) ယခုမြို့နယ်တွင် နေထိုင်သည်မှာ မည်မျှကြာပြီနည်း။ ( ) နှစ် ( ) လ

(၁၂) လွန်ခဲ့သော ၅ နှစ် အတွင်း သားသမီးအိမ် (သို့) ဆွေမျိုးအိမ်သို့ ပြောင်းရွှေ့ခြင်း ဘယ်နှစ်ကြိမ်ရှိသနည်း။

၁။ မရှိပါ ၂။ တခါနှစ်ခါ ၃။ ၃ ကြိမ် — ၅ ကြိမ် ၄။ ၆ ကြိမ် နှင့်အထက်

(၁၃) ပညာအရည်အချင်း (မေးမြန်းသူအတွက် မူလတန်းတက်ဖူးသည်ဆိုသည်မှာ မူလတန်းသို့တက်ရောက်ခဲ့ဖူးသော်လည်း ပြီးဆုံးသည်အထိ မရောက်ရှိဟု ဆိုလိုသည်။ အမြင့်ဆုံးပညာရေးကိုသာ ဝိုင်းပေးရန်)

၁။ စာမတတ်ပါ/ကျောင်းမနေပါ ၂။ မူလတန်းတက်ဖူး ၃။ မူလတန်းပြီး ၄။ အလယ်တန်း

၅။ အထက်တန်း ၆။ သက်မွေးဝမ်းကျောင်းပညာ ၇။ ကောလိပ်/တက္ကသိုလ်

၈။ ဘုန်းကြီးကျောင်းပညာရေး (ရေးတတ်ဖတ်တတ်)

(၁၄) လွန်ခဲ့သော ၁ နှစ်အတွင်း မည်သည့်အဖြစ်အပျက် ဖြစ်ခဲ့သနည်း။ မေးခွန်းတစ်ခုထက်မက ဖြေဆိုနိုင်သည်။

(၁) အလုပ်သစ် စတင်သည်
(၂) အလုပ်နား/အလုပ်ပြုတ်/ပင်စင်ယူသည်
(၃) သားသမီးများနှင့် အတူစနေသည်
(၄) တဦးတည်း စနေသည်
(၅) ဝင်ငွေ ပိုကောင်းလာသည်
(၆) ဝင်ငွေ လျော့ကျသွားသည်
(၇) မြေးအသစ်/ မြစ်အသစ်ထပ်မွေးသည်
(၈) ဇနီး/ ခင်ပွန်းကွယ်လွန်သည်
(၉) မိသားစု၊ ရင်းနှီးသောသူငယ်ချင်း ကွယ်လွန်သည်
(၁၀) သူငယ်ချင်း အသစ်ရသည်
(၁၁) အပြင်းအထန်နေမကောင်းဖြစ်သည်
(၁၂) နေမကောင်းသောမိသားစုဝင်ကို ပြုစုရသည်
(၁၃) အခြား (ဖော်ပြပါ ----------------)
(၁၄) ထူးခြားသော ပြောင်းလဲခြင်းမရှိပါ

မေးခွန်း (၁၉)။ သင့်မိသားစုအကြောင်းမေးခွန်းများ

(၁) သင့်အိမ်ထောင်ရေးအခြေအနေ

၁။ အိမ်ထောင်ရှိ ၂။ မုဆိုးဖို/မ ၃။ ကွာရှင်း ၄။ အိမ်ထောင်မပြုဖူးပါ

၅။ အခြား (ဖော်ပြပါ ----------------) (* ၄ ဟု ဖြေလျှင် (၃) သို့ ကျော်ပါ)

(၂) သင့်တွင် သားသမီး ရှိပါသလား။

၁။ ရှိပါသည်၊ အကုန် (သို့) အချို့အသက်ရှင်လျက်ရှိ

၂။ ရှိပါသည်၊ အားလုံး ဆုံးပါးသွားပြီ ၃။ သားသမီး မရှိပါ။

(၃) သင့်မိသားစုဖွဲ့စည်းပုံ

၁။ တယောက်တည်းနေ/အဆောင်နေ　　　　၂။ သွေးရင်းမိသားစုနှင့်နေ

၃။ သွေးရင်းမဟုတ်သောမိသားစုနှင့်နေ　　　　၄။ တရားရိပ်သာ၊ ဘိုးဘွားရိပ်သာစသဖြင့်

(၄) သင့်အိမ်တွင် သင်အပါအဝင် လူအရေအတွက် ဘယ်နှစ်ယောက်ရှိပါသလဲ။

------ ဦး

(၅) သင် မည်သူနှင့်အတူနေသနည်း။ (တစ်ခုထက်မက ဖြေနိုင်သည်)

၁။ မည်သူ့နှင့်မှမနေပါ　၂။ ဇနီး၊ ခင်ပွန်း　၃။ သား　၄။သမီး　၅။ သားမက်/ချွေးမ　၆။ မြေးများ

၇။ ညီအကိုမောင်နှမ　၈။ ဖခင်　၉။ မိခင်　၁၀။ ယောက္ခထီး　၁၁။ ယောက္ခမ

၁၂။ အခြား (ဖော်ပြပါ---------------------)

(၆) မိသားစု တစ်လပျှမ်းမျှဝင်ငွေကို အနီးစပ်ဆုံးဖော်ပြပါ။

၁။ (　　　　　　　　　) ကျပ်　　　　၂။ မသိပါ။

(၇) သင်၏ လက်ရှိစီးပွားရေးအခြေအနေကို အနီးစပ်ဆုံးဖော်ပြပါ။

၁။ အရမ်းခက်ခဲ　၂။ ခက်ခဲ　၃။ အသင့်အတင့်　၄။ အခြေအနေကောင်း　၅။ အလွန်ကောင်း

(၈) သင်ငယ်ငယ်တုံးက နေထိုင်ရသောလူမှုဘဝကို မည်သို့ထင်သနည်း။

၁။ အရမ်းကောင်း　၂။ ကောင်း　၃။ သာမန်　၄။ ညံ့　၅။ အရမ်းညံ့

(၉) သောက်ရေအရင်းအမြစ်ကိုရရန် မိနစ် ၃၀ ထက် ပိုသွားပြီး ယူရပါသလား။

၁။ သွားယူရသည်　　　　၂။ သွားမယူရပါ

(၁၀) အောက်ပါ အသုံးအဆောင် ပစ္စည်း များကို အခြားအိမ်များနှင့် မမျှဝေပဲ အသုံးပြုပါသလား။

၁။ ရေလောင်းအိမ်သာ/ဘိုထိုင်အိမ်သာ　　　　၁။ မျှဝေ　　၂။ မမျှဝေ

၂။ လေဝင်ပေါက်ပါသော တွင်းအိမ်သာ/အဖုံးပါအိမ်သာ　　　　၁။ မျှဝေ　　၂။ မမျှဝေ

(၁၁) သင့်အိမ်တွင် မိသားစုတစ်ဦးစီအတွက် ခြင်ထောင်ရှိပါသလား။

၁။ ရှိ　　　　၂။ ရှိ၊ သို့သော် မလုံလောက်ပါ　　　　၃။ ခြင်ထောင်မရှိပါ

မေးခွန်း (၂၀)။ ယခုလက်ရှိအလုပ်အကိုင်နှင့် ပတ်သက်သောမေးခွန်းများ

(၁) သင် အချိန်အကြာမြင့်ဆုံး လုပ်ခဲ့သောအလုပ်အကိုင်တစ်ခုကို ဖော်ပြပါ။

၁။ ပညာရှင်　　၂။ မန်နေဂျာ/စီမံ အုပ်ချုပ်သူ　　၃။ စာရေး　　၄။ အရောင်းအဝယ်

၅။ ကျွမ်းကျင်လုပါသား　　၆။ စိုက်ပျိုးရေး၊ သစ်တော၊ မွေးမြူရေး　　၇။ ကိုယ်ပိုင်အလုပ်

၈။ အခြား (ဖော်ပြပါ---------------)　　　　၉။ အလုပ်မရှိဖူးပါ

(၂) သင်၏ လက်ရှိအလုပ်အကိုင်အခြေအနေကို ဖော်ပြပါ။

၁။ အလုပ်အကိုင်ရှိ ၂။ အလုပ်မှအနားယူပြီး ၃။ အလုပ်မရှိဖူးပါ

(၃) အကယ်၍ သင် ၂ သို့မဟုတ် ၃ ကို ရွေးချယ်ခဲ့လျှင် ယခုလက်ရှိ သင်အလုပ်ရှာနေပါသလား။

၁။ ရှာ ၂။ မရှာ

မေးခွန်း (၂၁)။ သင့်မိသားစုတွင် အောက်ပါပစ္စည်းများ ပိုင်ဆိုင်ပါသလား။

ရေဒီယို	ရှိ	မရှိ	အဝတ်လျှော်စက်	ရှိ	မရှိ
အဖြူအမည်းရုပ်မြင်သံကြားစက်	ရှိ	မရှိ	ဂက်စ်မီးဖို	ရှိ	မရှိ
ရောင်စုံရုပ်မြင်သံကြားစက်	ရှိ	မရှိ	လျှပ်စစ်ထမင်းအိုး	ရှိ	မရှိ
ခွေပြစက် (DVD/ VCD	ရှိ	မရှိ	လေအေးပေးစက်	ရှိ	မရှိ
ပန်ကာ	ရှိ	မရှိ	စက်ဘီး	ရှိ	မရှိ
ရေခဲသေတ္တာ	ရှိ	မရှိ	မော်တော်ဆိုင်ကယ်	ရှိ	မရှိ
ကွန်ပြူတာ	ရှိ	မရှိ	ကား/ ထော်လာဂျီ	ရှိ	မရှိ
ဝယ်ယူထားသောအိမ်ထောင်ပရိဘောဂ	ရှိ	မရှိ	မိုက်ခရိုဝေ့မီးဖို	ရှိ	မရှိ
ကက်ဆက်/ MP3	ရှိ	မရှိ	လက်ကိုင်ဖုန်း	ရှိ	မရှိ
			အင်တာနက်	ရှိ	မရှိ

မေးခွန်း (၂၂)။ သင့်မိသားစု၏ သင့်အပေါ်ပြုမှုဆက်ဆံသောကိစ္စနှင့် ပတ်သက်သောမေးခွန်းများ

(မေးမြန်းသူအတွက်- သင်မှလွဲ၍ ဖြေဆိုသူအနီးတွင် တစ်စုံတစ်ယောက်ရှိပါက ခေတ္တဖယ်ခိုင်းပြီးမှ ဤမေးခွန်းကိုမေးပါ)

(၁)လွန်ခဲ့သောနှစ်က သင့်အပေါ် သင့်မိသားစုက ရုပ်ပိုင်းဆိုင်ရာ အကြမ်းဖက်မှု (ရိုက်ပုတ်ခြင်း)၊ ကန်ကျောက်ခြင်း၊ ပစ္စည်းများဖြင့်ပစ်ပေါက်ခြင်း၊ အခန်းတွင်းပိတ်လှောင်ထားခြင်း ရှိဖူးပါသလား။

၁။ ရှိပါသည် ၂။ မရှိပါ

(၂) လွန်ခဲ့သောနှစ်အတွင်း သင့်ကို လေးစားမှုလျော့နည်းစေသောအပြုအမူ (ဆဲဆိုခြင်း) မကောင်းသောမှတ်ချက်ပြုခြင်း၊ လျစ်လျူရှုခြင်း) ရှိဖူးပါသလား။

၁။ ရှိပါသည် ၂။ မရှိပါ

(၃) သင့်မိသားစုက သင်ပိုင်သောငွေကို သဘောတူညီမှုမရှိဘဲ (ခွင့်မတောင်းဘဲ သုံးလေ့ရှိပါသလား)။

၁။ ရှိပါသည် ၂။မရှိပါ

မေးခွန်း (၂၃)။ အောက်ပါမေးခွန်းများသည် သင်၏အနာဂတ်နှင့် ပတ်သက်သည်။

(၁)သင့်ရဲ့ဘဝလုပ်ငန်းဆောင်တာများလုပ်နိုင်မှုနှင့် သိမှတ်မှုစွမ်းရည်ကျဆင်းလာသောအခါ (သို့) မသန်မစွမ်းဖြစ်သောအခါ သင့်ကို တစ်ဦးတစ်ယောက်က ဂရုတစိုက်ပြုစုမည်ဟု မျှော်လင့်ပါသလား။

၁။ မျှော်လင့်ပါသည် ၂။ မမျှော်လင့်ပါ ၃။ မသိပါ

(၂) အကယ်၍ သင်သည် မေးခွန်းနံပါတ် (၁) မှ အဖြေ ၁ ကို ရွေးချယ်ခဲ့လျှင်၊ မည်သူ(တွေ)က ဂရုတစိုက်ပြုစုမည်ဟု ထင်ပါသလဲ။ တခုထက်မက ဖြေဆိုနိုင်သည်။

၁။ ခင်ပွန်း/ဇနီး ၂။ သားသမီး ၃။ သားမက်၊ ချွေးမ ၄။ ညီအကိုမောင်နှမများ

၅။ ဆွေမျိုးများ ၆။ မိတ်ဆွေသူငယ်ချင်းများ ၇။ အိမ်နီးနားချင်းများ

၈။ အခြား (ဖော်ပြပါ --------------------------------)
