# Supplementary material for: Association between depressive symptoms and objective/subjective socioeconomic status among older adults of two regions in Myanmar
Source: PLoS One. 2021 Jan 28;16(1):e0245489. doi: 10.1371/journal.pone.0245489 (PMC7842968; doi:10.1371/journal.pone.0245489)
Supplement: S2 Questionnaires — (PDF) [file pone.0245489.s002.pdf]

Registration sheet for research project, “Healthy Ageing in Myanmar”

Date of Interview (dd/mm/yyyy): 【day_18】 / 【mth_18】 /2018

W	X	Y	Y	Z	Z

ID (W:Region, X:Township, YY:Ward/Village tract, ZZ:Person)
【rgn_18】 【tsp_18】 【wrd_18】 【psn_18】

Name* (respondent): 【name_18】 **Sex*:** 1. Male 2. Female 【sex_2_18】

Date of birth (dd/mm/yyyy): 【dob_18】 / 【mob_18】 / 【yob_18】 **Age*:** 【age_ysl18】 y.o.

Address*: 【add_18】

These data will be obtained from ledger list.

Contact phone numbers of respondent, spouse, children, and relatives (for follow up purpose):

No	Phone number	Who’s number
1	【phn_a_18】	【ph5a_18】<br>1. Respondent 2. Spouse 3. Son 4. Daughter 5. Relative (【ph5aot_18】)
2	【phn_b_18】	【ph5b_18】<br>1. Respondent 2. Spouse 3. Son 4. Daughter 5. Relative (【ph5bot_18】)
3	【phn_c_18】	【ph5c_18】<br>1. Respondent 2. Spouse 3. Son 4. Daughter 5. Relative (【ph5cot_18】)
4	【phn_d_18】	【ph5d_18】<br>1. Respondent 2. Spouse 3. Son 4. Daughter 5. Relative (【ph5dot_18】)
5	【phn_e_18】	【ph5e_18】<br>1.Respondent 2. Spouse 3. Son 4. Daughter 5. Relative (【ph5eot_18】)

Objective measurement

1. Blood pressure (please measure by the same arm)

1st: 【k_sbp1_2018】 / 【k_dbp1_2018】 **mmHg 2nd:** 【k_sbp2_2018】 / 【k_dbp2_2018】 **mmHg**

2-1). Body height: (actual measure) 【htac_18】 **. cm 2-2).(Demi-span)** 【htdemi_18】 **. cm****

**Women--- 1.35 X demi-span (cm) + 60.1 -> Height 【htdemical_18】 . cm

Men------- 1.40 X demi-span (cm) + 57.8 -> Height 【htdemical_18】 . cm

Circle either of 2-1) or 2-2) which you used for setting BW scale (in case that a respondent’s back is bent, use 2-2))

3. Body weight: 【wt_18】 **. kg**

4. Body composition 1) FAT: 【fat_18】 **. % 2) MASCLE:** 【msl_18】 **. kg**

5. Abdominal circumference: 【ac_18】 **. cm**

6. Grip strength (please measure the same side) 1st: 【gs1_18】 **kg 2nd:** 【gs2_18】 **kg**

Please, make an interview to respondent directly in person to person situation (NO person surrounded as much as possible at least from Q22).
If anyone is besides respondent, please specify bellow:

1. Spouse 2. Children 3. Brother/sister 4. Parent(s) 5. Grandchildren
【ardsp_18】【ardch_18】【ardbr_18】【ardpa_18】【ardgc_18】

6. Relative(s) 7. Neighbor 8. Friend 9. Other ( )
【ardrl_18】【ardnb_18】【ardfr_18】【ardot_18】【ardottxt_18】

Q0. Abbreviated Mental Test (AMT)
(*To interviewer: Please ask questions 1-10 in this list and record all answers.*)

QUESTION	Incorrect	Correct
1.Age? 【amt2ag_18】		
2. Time? (to nearest hour) 【amt2tm_18】		
3. Address for recall at end of test? 【amt2ad_18】		
4. Year? 【amt2yr_18】		
5. Name of this place? 【amt2pl_18】		
6. Identification of two person (relative, surveyor, etc)? 【amt2id_18】		
7. Date of birth? 【amt2db_18】		
8. Year of Demonstration 8888? 【amt2yd_18】		
9. Name of present the President? 【amt2pr_18】		
10. Count backwards 20 to 1? 【amt2ct_18】		
Total【amt2to_18】		

Continue to the next section, if the respondent is eligible for the survey.
Score of Q0. Abbreviated Mental Test (AMT) is 7 or more, continue the interview.
Score of Q0. Abbreviated Mental Test (AMT) is 6 or less, FINISH interview.

Information of surveyor

Name of surveyor: 1._ _【nms1_18】_ 2._【nms2_18】____

Q1. First, we would like to ask you about your physical status.

W	X	Y	Y	Z	Z

1) How is your current health status? 【srh_4_18】
1. Excellent 2. Good 3. Fair 4. Poor

2) Do you need any nursing care or assistance in your daily life from anyone? 【adl3ra18】
1. I do not need nursing care or assistance. 2. I need nursing care or assistance but do not receive it.
3. I need and receive nursing care or assistance.

3) For at least the past 6 months, to what extent have you been limited, because of a health problem in activities people usually do? Would you say you have been…【limit_3_18】
1. Severely limited 2. Limited but not severely 3. Not limited at all

Q2. Do you have any difficulties?

Seeing
1-1) Do you have any difficulty of seeing?【eye4see18】
1. No difficulty 2. Yes, some difficulty 3. Yes, a lot of difficulty 4. Cannot do at all

1-2) Do you usually use glasses?【eye3gls18】
1. Yes, without difficulty 2. Yes, with difficulty 3. No

1-3) Have you ever been to an eye clinic due to difficulty of seeing?【eye2cl18】
1. Yes 2. No

Hearing
2-1) Do you have any difficulty of hearing?【ear4hr18】
1. No difficulty 2. Yes, some difficulty 3. Yes, a lot of difficulty 4. Cannot do at all

2-2) Do you usually use a hearing aid device?【ear3dev18】
1. Yes, without difficulty 2. Yes, with difficulty 3. No

2-3) Have you ever been to an ENT (ear, nose, and throat) clinic due to difficulty of hearing?【ear2cl18】
1. Yes 2. No

Walking
3-1) Do you have any difficulty of walking, climbing steps, carrying items?【wlk4dif18】
1. No difficulty 2. Yes, some difficulty 3. Yes, a lot of difficulty 4. Cannot do at all

3-2) Do you usually use a walking aids (e.g. walker, stick)【wlk2aid18】
1. Yes 2. No

3-3) Have you ever been to seeing a doctor due to walking difficulty?【wlk2cl18】
1. Yes 2. No

Remembering
4) Do you have any difficulty of remembering or concentrating? 【rem_4_18】
1. No difficulty 2. Yes, some difficulty 3. Yes, a lot of difficulty 4. Cannot do at all

Q3. The following questions are about activities of daily living.
For each category, circle the number of the answer that applies.

Bathing	1. Bathes self completely or needs help in bathing only a single part of the body such as the back, genital area or disabled extremity. 【adl2ba_18】	2. Need help with bathing more than one part of the body, getting in or out of the tub or shower. Requires total bathing.
Dressing	1. Get clothes from closets and drawers and puts on clothes and outer garments complete with fasteners. May have help tying shoes. 【adl2dr_18】	2. Needs help with dressing self or needs to be completely dressed.
Toileting	1. Goes toilet, gets on and off, arranges clothes, cleans genital area without help. 【adl2to_18】	2. Needs help transferring to the toilet, cleaning self or uses bedpan or commode.
Transferring	1. Moves in and out of bed or chair unassisted. Mechanical transfer aids are acceptable. 【adl2tr_18】	2. Needs help in moving from bed to chair or requires a complete transfer.
Continence	1. Exercises complete self control over urination and defecation. 【adl2co_18】	2. Is partially or totally incontinent of bowel or bladder.
Feeding	1. Gets food from plate into mouth without help. Preparation of food may be done by another person. 【adl2fe_18】	2. Needs partial or total help with feeding or requires parenteral feeding.

Q4. The following questions are about your past/present medical history.

1). Have you ever been diagnosed as hypertension by a medical doctor, nurse or health officer? 【ht3dgns18】
1. Yes 2. No 3. I don't know

2). Have you been prescribed any antihypertensive (or blood pressure-lowering medicine)? 【ht4med18】
1. Yes (Western medicine)
2. Yes (Traditional medicine)
3. No -> go to 5)
4. I don't know -> go to 5)

3). Do you take antihypertensive as instructed? 【ht2tret18】
1. Yes 2. No

4). If you don't take your medication regularly, what are the reasons you don't take them as directed? Circle all that apply.
1. I cannot afford the cost 【ht9rsnco18】
2. Medication is not easily available (ex: too far to take medication from home to health facilities, pharmacies etc) 【ht9rsnna18】
3. I do not like to take medications 【ht9rsndl18】
4. I only take them when I feel that I need them. 【ht9rsnif18】
5. I do not like the side effects of the medication. 【ht9rsnse18】
6. I prefer traditional medicine. 【ht9rsnam18】
7. I forget to take medication regularly. 【ht9rsnft18】
8. I don't know 【ht9rsnid18】
9. Other 【ht9rsnot18】 ______【ht9rsnottxt18】_____

5) Have you had any complications from your hypertension?
1. No 2. Renal disease 3.Stroke 4. Retinopathy 5.Cardiovascular
【ht7cmplno18】【ht7cmplrd18】【ht7cmplst18】【ht7cmplrt18】【ht7cmplcv18】
6. Other【ht7cmplot18】 ___【ht7cmplottxt18】___ 7. I do not know【ht7cmplid18】

6-1) Did you measure your blood pressure last 12 months ago?
1.Yes 2.No (if No-→ go to Q 7)【ht2mbp18】

6-2) Compared to 12 months ago, is your blood pressure: 【ht4ctl18】
1.Better 2.Same 3.Worse 4.I don't know

7) Circle the number of all diseases as which you have been diagnosed by a doctor.

1. None 【dgns2no18】	2. I don't know 【dgns2dk18】
3. Stroke (e.g., brain hemorrhage, cerebral infarction) 【dgns2st18】	4. Heart disease【dgns2hd18】
5. Diabetes【dgns2dm18】	6. Hyperlipidemia (lipid abnormality) 【dgns2hl18】
7. Respiratory disease (e.g., pneumonia, bronchitis) 【dgns2rd18】	8. Gastrointestinal, liver, or gallbladder disease 【dgns2gl18】
9. Kidney or prostate gland disease 【dgns2kp18】	10. Musculoskeletal disease (e.g., osteoporosis, arthritis) 【dgns2oa18】
11. Traumatic injury (e.g., fall, fracture) 【dgns2fx18】	12. Cancer (malignant tumor) 【dgns2ca18】
13. Blood or immune system disease 【dgns2im18】	14. Depression【dgns2dp18】
15. Dementia (e.g., Alzheimer's disease) 【dgns2dt18】	16. Parkinson's disease (an illness in which your muscles become very weak and your arms and legs become shaky, slow, stiff and rigid) 【dgns2ps18】
17. Eye disease【dgns2vi18】	18. Ear disease【dgns2he18】
19. Tuberculosis【dgns2tb18】	20. HIV【dgns2hi18】
21. Malaria【dgns2ml18】	22. Gynecological problem【dgns2gn18】
23. Other ( ) 【dgns2ot18】【dgns2ottxt18】	

Q5. The following questions are about your health care.

1) Have you been ill/sick in the past 12 months? 【hlt4is18】
1. No →Go to Q6
2. Yes, I have, and I am still ill/sick now.
3. Yes, I have, but I am not ill/sick now.
4. I do not remember →Go to Q6

2) Did you see a doctor or nurse when you were ill/sick in the past 12 months? 【hlt4dn18】
1. Yes, I always see a doctor or nurse.
2. Yes, I sometimes see a doctor or nurse.
3. No, I did not -> Go to Q6.
4. I do not remember -> Go to Q6.

3)-1 Did you hesitate to visit health facilities even you are ill/sick in the past 12 months? 【hlt2hv18】
1. Yes 2. No -> Go to Q6

3-2) If you answer Yes in 3-1), please specify the reason(s) why you hesitated to visit health facility. Circle all that apply.

1. It's too expensive for me【hlt8hhte18】
2. The fares to/from the clinical department are too high for me【hlt8hhth18】
3. I don't know which department to visit for the medical care I need【hlt8hhwd18】
4. Health facilities are too far from my home【hlt8hhtf18】
5. I don't think I need medical care【hlt8hhdn18】
6. I don't have time to see a doctor【hlt8hhht18】
7. I don't have health insurance【hlt8hhhi18】
8. Other【hlt8hhot18】 (【hlt8hhottxt18】)

4) Which health facilities did you use in the past 12 months? How much did you pay for your medical treatment/care (i.e. your "out-of-pocket costs") for the visit(s)? Please rate each medical cost following the criteria [(5) very expensive, (4) Expensive, (3) Appropriate, (2) Cheap, (1) Very cheap, (0) don't know]

**Treatment facility**	**Please tick if you used. If nothing used, please leave it as blank.**	**Total amount in the past 12 months (Kyat)<br>If you don't know, please leave it as blank.**	**Rating [(6) Don't know (5) very expensive,<br>(4) Expensive,<br>(3) Appropriate,<br>(2) Cheap,<br>(1) Very cheap]**
**Government Health Sector**			
1.Government Hospital	hlt2gh18	hltgham18	hlt6ghrt18
2.Government Health Center (RHC)	hlt2ghc18	hltghcam18	hlt6ghcrt18
3.Government Health Post (Sub Center)	hlt2ghp18	hltghpam18	hlt6ghprt18
4.Public Village Health Worker (Voluntary Health Worker)	hlt2pv18	hltpvam18	hlt6pvrt18
5.Government Mobile Clinic	hlt2gm18	hltgmam18	hlt6gmrt18
6. Public UHC Center	hlt2pu18	hltpuam18	hlt6purt18
7. Public Traditional Medical Clinic	hlt2pt18	hltptam18	hlt6ptrt18
8. Other Public Medical Sector	hlt2op18	hltopam18	hlt6oprt18
**Non-Government Sector**			
9. Marie Stopes	hlt2ms18	hltmsam18	hlt6msrt18
10. Myanmmart Red Cross	hlt2mr18	hltmram18	hlt6mrrt18
11. PSI/M (SUN)	hlt2ps18	hltpsam18	hlt6psrt18
12. MMA Private Sector	hlt2mp18	hltmpam18	hlt6mprt18
13. Other NGO Sector	hlt2on18	hltonam18	hlt6onrt18
**Private Sector**			
14. Private Hospital/Clinic	hlt2ph18	hltpham18	hlt6phrt18
15. Pharmacy	hlt2pm18	hltpmam18	hlt6pmrt18
16. Private Doctor	hlt2pd18	hltpdam18	hlt6pdrt18
17. Private Mobile Clinic	hlt2pc18	hltpcam18	hlt6pcrt18
18. Traditional Medical Clinic	hlt2tm18	hlttmam18	Hlt5tmrt18
19. Other Private Medical Sector	hlt2om18	hltomam18	hlt6omrt18
**Other source**			
20. Shop	hlt2sh18	hltsham18	hlt6shrt18
21. Traditional Practitioner	hlt2tp18	hlttpam18	hlt6tprt18

22. Other( )	hlt2oth18	hltotham18	hlt6othrt18
**Others**			
23. Not sure	hlt2ns18	hltnsam18	hlt6nsrt18

Q6. The following questions are about oral condition & medical check-up.

1) How many natural teeth do you presently have? "Natural teeth" includes repaired teeth by a crown. Including wisdom teeth, there are a total of 32 permanent teeth【teeth5_18】.
   1. I have no natural teeth
   2. I have 1 to 4 natural teeth
   3. I have 5 to 9 natural teeth
   4. I have 10 to 19 natural teeth
   5. I have 20 or more natural teeth

2) How often do you, or someone else, brush your teeth? 【to5fq18】
   1. At least three times a day 2. Twice a day 3. Once a day 4. Not daily 5. Never

3) Have you ever had a medical check-up at a health center, your workplace, a medical institution, or another place? 【exam4_18】
   1. I had one within a year.
   2. I had one sometime between 1 and 4 years ago.
   3. I had one more than 4 years ago.
   4. I've never had one.

Q7. The following questions are about your eating, drinking, and smoking status.

1) Do you find chewing hard food more difficult compared to half a year earlier? 【tok2ea18】
   1. Yes 2. No

2) During last 6 months, have you ever choked on tea or soup? 【tok2mu18】
   1. Yes 2. No

3) During last 6 months, are you bothered with dry mouth? 【tok2ku18】
   1. Yes 2. No

4) How often did you eat meat or fish over the past month? 【fq7prt18】
   1. Twice a day or more 2. Once a day 3. Four to six times a week 4. Two or three times a week
   5. Once a week 6. Less than once a week 7. None

5) How often did you eat fruits and vegetables over the past month? 【fq7veg18】
   1. Twice a day or more 2. Once a day 3. Four to six times a week 4. Two or three times a week
   5. Once a week 6. Less than once a week 7. None

6) Do you drink alcohol? 【alcl4_18】
   1. Yes, I currently drink.
   2. I quit drinking recently (less than 5 years ago) and don't drink now.
   3. I quit drinking more than 5 years ago and don't drink now.
   4. I never drank.

7) Do you smoke cigarettes? 【smok5_18】
   1. I smoke almost every day.
   2. I smoke sometimes (smoked in the past 30 days).
   3. I quit smoking recently (less than 5 years ago) and don't smoke now.
   4. I quit smoking more than 5 years ago and don't smoke now.
   5. I never smoked.

8) Do you chew betel with tobacco? 【btl5_18】
   1. I chew almost every day.
   2. I chew sometimes (chewed in the past 30 days).
   3. I quit chewing recently (less than 5 years ago) and don't chew now.
   4. I quit chewing more than 5 years ago and don't chew now.

5. I never chewed.

9) Whom do you eat meals with? Circle all that apply.
1. None (by myself) 【ml2al18】 2. My spouse 【ml2sp18】 3. My child 【ml2ch18】 4. My grandchild 【ml2gc18】 5. My friend 【ml2fr18】 6. Other ( ) 【ml2ot18】【ml2ottxt18】

10) How often do you eat meals with someone else? 【ml5op18】
1. Almost every day (4-7 times a week) 2. A few times (1-3) a week 3. A few times (1-3) a month
4. A few times a year (Less than 1 time a month) 5. Rarely (Less than 1 time a year)

Q8. The following questions are about going out in daily life and your routine activities.

1) How often do you go out (including to the field or immediate neighborhood, for shopping, to a hospital, and to a mosque, monastery, and temple etc.)? 【gout6fq18】
1. Four or more times a week 2. Two or three times a week 3. Once a week
4. One to three times a month 5. Less than once a month 6. Rarely

2) What do you use for transport when you go out? Circle the numbers of all the answers that apply.
1. On foot (don't use 2 to 16) 【gout2wk18】 2. Bicycle 【gout2bi18】 3. Motorcycle 【gout2mo18】 4. Car (drive myself) 【gout2dv18】
5. Car (someone else drives) 【gout2rd18】 6. Train 【gout2tr18】 7. Public bus 【gout2pb18】 8. Boat (steer myself) 【gout2st18】
9. Boat (someone else steers) 【gout2rb18】 10. Wheelchair 【gout2wc18】 11. Motorized wheelchair (handcart) 【gout2ct18】
12. Walker or rollator 【gout2sc18】 13. Taxi 【gout2tx18】 14.Trishaw 【gout2tw18】 15. Tractor or trailer 【gout2tt18】 16. Other ( ) 【gout2ot18】【gout2ottxt18】

3) Have you had any falls over the past year? 【fall3xp18】
1. Many times 2. Once 3. None

4) Are you worried about falls? 【fall4fe18】
1. Yes, very much 2. Yes, somewhat 3. Only a little 4. No

5) Do you go up stairs without holding onto the handrail or the wall? 【fall3ri18】
1. Yes, I can and do. 2. Yes, I can but usually don't. 3. No, I can't.

6) Do you get up out of chairs without holding anything? 【fall3st18】
1. Yes, I can and do. 2. Yes, I can but usually don't. 3. No, I can't.

7) How long do you walk a day on average? 【walk5tm18】
1. No, I can’t walk. 2. Less than 30 minutes
3. 30 to 59 minutes 4. 60 to 89 minutes
5. 90 minutes or more

Q9. The following questions are about your daily life. Circle the number of the appropriate answer.

1) Can you go out alone by train or bus?【iadl3bt_18】	1. always go with train or bus<br>2. Go with train or bus, but not always<br>3. Don’t go at all
2) Can you go shopping for daily necessities? 【iadl3pc_18】	1. always go with train or bus<br>2. Go with train or bus, but not always<br>3. Don’t go at all

3) Can you cook for yourself? 【iadl3me_18】	1. always cook<br>2. Cook, but not always<br>3. Don’t cook at all
4) Do you read newspapers? 【iadl3np18】	1. Yes 2. No 3. can’t read
5) Do you read books or magazines? 【iadl3bm18】	1. Yes 2. No 3. can’t read
6) Are you interested in health-related articles or TV programs? 【iadl2hl18】	1. Yes 2. No
7) Do you visit your friends’ homes? 【iadl2vf18】	1. Yes 2. No
8) Do you give advice to your family members or friends? 【iadl2gc18】	1. Yes 2. No
9) Can you visit people who have fallen ill? 【iadl2vp18】	1. Yes 2. No
10) Do you start conversations with young people? 【iadl2ty18】	1. Yes 2. No
11) Do you walk without stopping for about 15 minutes? 【iadl3wa18】	1. Yes, I can and do.<br>2. Yes, I can but usually don't.<br>3. No, I can't.
12) Do people around you notice your forgetfulness, for example, by telling you that you often ask the same thing? 【iadl2fo18】	1. Yes 2. No
13) Do you sometimes forget what date it is today? 【iadl2da18】	1. Yes 2. No
14) Do you think you are forgetful these days? 【iadl2fg18】	1. Yes 2. No

Q10. The following questions are about your activities related to your hobbies.

1) Do you have hobbies? 【hoby2_18】
1. Yes 2. No → Skip to Q11

2) What are the hobbies? Please write down your hobbies. 【hoby_txt18】
( )

Q11. The following questions are about clubs and groups you are currently engaged in.

1) How often do you attend activities for the following groups?

(1) Religious group【cmnt6rl18】
1. Four or more times a week 2. Two or three times a week 3. Once a week
4. One to three times a month 5. A few times in a year 6. Never

(2) Volunteer group (A group that does a job willingly without being paid: e.g. Red cross, Charity group, and Funeral services etc.) 【cmnt6vl18】
1. Four or more times a week 2. Two or three times a week 3. Once a week
4. One to three times a month 5. A few times in a year 6. Never

(3) Sports groups or clubs【cmnt6sp18】
1. Four or more times a week 2. Two or three times a week 3. Once a week
4. One to three times a month 5. A few times in a year 6. Never

(4) Hobby groups【cmnt6hb18】
1. Four or more times a week 2. Two or three times a week 3. Once a week

4. One to three times a month 5. A few times in a year 6. Never

(5) Community meetings (e.g. gathering of township association, homeage paying ceremony) 【cmnt6cm18】
1. Four or more times a week 2. Two or three times a week 3. Once a week
4. One to three times a month 5. A few times in a year 6. Never

(6) Political meetings or events【cmnt6pl18】
1. Four or more times a week 2. Two or three times a week 3. Once a week
4. One to three times a month 5. A few times in a year 6. Never

2) What is the group you are most frequently involved in among the above (1)-(6)? 【cmnt6fi18】
1. Religious group 2. Volunteer group 3. Sports groups or clubs
4. Hobby groups 5. Community meetings 6. Political meetings or events

Q12. For the group you are involved in most frequently, selected in Q11-2), circle the numbers for the answers that best apply.

1 Gender ratio【grp4ge_18】
1. Men or women only 2. More men than women
3. More women than men 4. Roughly an equal proportion of men to women

2 Area of residence【grp2ar_18】
1. Only people from the same ward/village tract 2. Some people from other ward/village tract

3 Age composition1. Mostly people of the same generation【grp2ag_18】
2. Mixture of different generations (age difference of at least 20 years)

4 Social standing【grp2so_18】
1. There is a person or people of high social standing (e.g., residents' association official, politician, city council member, company or trade association executive, doctor, lawyer, etc.)
2. There are no people of high social standing

5 Ethnic composition【grp2et_18】
1. The group consists of people of different ethnicities
2. The group consists of ethnically the same people

Q13. The following questions are about your relationships with your friends.

1) How often do you see your friends/acquaintance? 【meet6fr18】
1. Four or more a week 2. Two or three times a week 3. Once a week
4. One to three times a month 5. A few times a year 6. Hardly/None

2) How many friends/acquaintances have you seen over the past month? Count the same person as one, no matter how many times you have seen him/her. 【num5fr18】
1. None 2. 1 to 2 3. 3 to 5 4. 6 to 9 5. 10 or more

3) Who do you meet often? Circle the numbers of all the answers that apply.
1. Neighbors or acquaintance living in the same area 【frie2ne18】
2. Childhood friend 【frie2ch18】
3. Friend from your school days 【frie2st18】
4. Colleague or former colleague 【frie2wo18】
5. Friends sharing the same interest with me 【frie2ho18】
6. Friend in the same clubs or groups 【frie2gr18】
7. Other 【frie2ot18】
8. I do not have friends. 【frie2no18】

Q14. The following questions are about your social relationships.

1) Among those who are usually interacting with you, are there any people who live in the following places? Please answer about each of your family, relatives, co-workers, or other friends/acquaintances. Circle all that apply.

	Family (spouse, children, grandchildren, parents, siblings)	Other relatives	Work-related friend/ acquaintance	Non-work-related friend/ acquaintance	No one
1. Those who live within 10 minutes on foot	1 【lvft2_fa18】	2 【lvft2_or18】	3 【lvft2_wo18】	4 【lvft2_no18】	5 lvft2_nn18
2. Those who live within the same township, taking more than 10 minutes on foot	1 【lvto2_fa18】	2 【lvto2_or18】	3 【lvto2_wo18】	4 【lvto2_no18】	5 lvto2_nn18
3. Those who are not in the same township, but live within the same state/region	1 【lvst2_fa18】	2 【lvst2_or18】	3 【lvst2_wo18】	4 【lvst2_no18】	5 lvst2_nn18
4. Those who live outside the state/region where you live in	1 【lvou2_fa18】	2 【lvou2_or18】	3 【lvou2_wo18】	4 【lvou2_no18】	5 lvou2_nn18
5. Those who live in a foreign country	1 【lvfo2_fa18】	2 【lvfo2_or18】	3 【lvfo2_wo18】	4 【lvfo2_no18】	5 lvfo2_nn18

2) Generally speaking, would you say that most people can be trusted? 【for_3_18】
1. Yes 2. No 3. It depends on the situation

Q15. The following questions are asking about mutual assistance with people around you.

1) Do you have someone who listens to your concerns and complaints? Circle all that apply.
1. Spouse 【lsnd2fm18】 2. Children living together 【lsnd2ch18】 3. Children or relatives living apart 【lsnd2la18】
4. Brother/sister, relative, parents, grandchildren 【lsnd2of18】 5. Neighbor 【lsnd2ne18】 6. Friend 【lsnd2fr18】 7. Other ( ) 【lsnd2ot18】 【lsnd2ottxt18】
8. I do not have such person. 【lsnd2no18】

2) Do you listen to someone's concerns or complaints? Circle all that apply.
1. Spouse 【lstn2fm18】 2. Children living together 【lstn2ch18】 3. Children or relatives living apart 【lstn2la18】
4. Brother/sister, relative, parents, grandchildren 【lstn2of18】 5. Neighbor 【lstn2ne18】 6. Friend 【lstn2fr18】 7. Other ( ) 【lstn2ot18】 【lstn2ottxt18】
8. I do not have such person. 【lstn2no18】

3) Do you have someone who looks after you when you are sick and confined to a bed for a few days? Circle all that apply.
1. Spouse 【card2fm18】 2. Children living together 【card2ch18】 3. Children or relatives living apart 【card2la18】
4. Brother/sister, relative, parents, grandchildren 【card2of18】 5. Neighbor 【card2ne18】 6. Friend 【card2fr18】 7. Other ( ) 【card2ot18】 【card2ottxt18】
8. I do not have such person. 【card2no18】

4) Do you look after someone when he/she is sick and confined to a bed for a few days? Circle all that apply.
1. Spouse 【wcar2fm18】 2. Children living together 【wcar2ch18】 3. Children or relatives living apart 【wcar2la18】
4. Brother/sister, relative, parents, grandchildren 5. Neighbor 6. Friend 7. Other ( )

【wcar2of18】 【wcar2ne18】【wcar2fr18】【wcar2ot18】【wcar2ottxt18】
8. I do not have such person【wcar2no18】

5) Do you have someone you can consult with when you are in trouble? Circle all that apply.
1. Family member living together 【wi2fa18】 2. Children living apart 【wi2ch18】 3. Siblings/relatives/parents/grandchildren 【wi2bs18】
4. Neighbors/friends 【wi2ne18】 5. Community association/neighborhood association/senior club 【wi2se18】
6. Social welfare council/commissioned welfare volunteer 【wi2so18】

7. Physicians/dentists/nurses 【wi2do18】 8. Regional support centers/local government offices 【wi2ci18】
9. Other ( ) 【wi2ot18】【wi2ottxt18】 10. I do not have such person. 【wi2no18】

Q16. The following questions are about the area where you live.

1) Do you think people living in your area can be trusted in general? 【fot_5_18】
1. Very 2. Moderately 3. Neutral 4. Not really 5. Not at all

2) Do you think people living in your area try to help others in the most of situations? 【cmn5cntr18】
1. Very 2. Moderately 3. Neutral 4. Not really 5. Not at all

3) Do you like your area you live? 【cmn5at18】
1. Very 2. Moderately 3. Neutral 4. Not much 5. Not at all

4) Are you concerned about crime in your community? 【cmn5po10】
1. Very 2. Moderately 3. Neutral 4. Not much 5. Not at all

5) What kind of interactions do you have with people in your neighborhood? 【acqu4_18】
1. Mutual consultation, lending and borrowing daily commodities, and cooperation in daily life
2. Daily chatting
3. No more than exchanging greetings
4. None, not even greetings

6) Are the following present within walking distance of your home (within about half mile or 15-30 min walk)?
(1) Parks or foot paths suitable for exercise or walking 【env5prk18】
1. Yes 2. No 3. I don't know

(2) Shops, facilities, or mobile retailers selling fresh foods (e.g. meat, fish, vegetables, fruits) 【env5grc18】
1. Yes 2. No 3. I don't know

Q17. Circle the number of the appropriate answer in the following questions.
If respondents don't want to answer, leave it as blank.

1) Are you satisfied with your current life? 【gds_2sf18】	1. Yes 2. No
2) Do you sometimes feel there is no point in living? 【gds_2sa18】	1. Yes 2. No
3) Do you think your energy for daily life or your interest in what's going on in the world has been decreasing? 【gds_2ai18】	1. Yes 2. No

4) Do you feel your life is empty? 【gds_2em18】	1. Yes 2. No
5) Do you often feel bored? 【gds_2br18】	1. Yes 2. No
6) Do you usually feel good? 【gds_2fg18】	1. Yes 2. No
7) Do you feel something bad is going to happen? 【gds_2be18】	1. Yes 2. No
8) Do you think you are fortunate? 【gds_2hp18】	1. Yes 2. No
9) Do you often feel helpless?【gds_2nd18】	1. Yes 2. No
10) Do you prefer staying at home over going out?【gds_2hm18】	1. Yes 2. No
11) Do you think you are more forgetful than others?【gds_2fr18】	1. Yes 2. No
12) Do you think life is wonderful? (i.e., Do you think your life is very good, pleasant or enjoyable?) 【gds_2lb18】	1. Yes 2. No
13) Do you feel full of energy? 【gds_2vt18】	1. Yes 2. No
14) Do you think there is no hope in your life? 【gds_2nh18】	1. Yes 2. No
15) Do you think others are better off than you are? 【gds_2oc18】	1. Yes 2. No

Q18. The following questions are about your personal characteristics. Circle the number of the answer the best applies.

1) Sex【sex_2_hr18】
1. Male 2. Female

2) Age【age_ysl_hr18】
[ ] years old

3) What is your ethnicity? Circle all that apply.
1. Bamar 【et2bam_18】 2. Kachin 【et2kac_18】 3. Kayah 【et2kah_18】 4. Kayin 【et2kan_18】 5. Chin 【et2chi_18】 6. Mon 【et2mon_18】 7. Rakhine 【et2rak_18】 8. Shan 【et2sha_18】
9. Other Myanmar indigenous 【et2otm_18】 10. Others 【et2oth_18】 11. I don't want to answer 【et2no_18】

4) How often did you participate in charity events (ex. serving food, mentoring youth)? 【per6ch_18】
1. Four or more a week 2. Two or three times a week 3. Once a week
4. One to three times a month 5. A few times a year 6. None

5) How often do you donate? 【per6do_18】
1. Four or more a week 2. Two or three times a week 3. Once a week
4. One to three times a month 5. A few times a year 6. None

6) What is your religion? 【per6re_18】
1. Buddhism 2. Islam 3. Christian 4. Hindu 5. Other ( ) 6. None

7) How often do you go to a temple, mosque or church etc? 【per6go_18】
1. Four or more a week 2. Two or three times a week 3. Once a week
4. One to three times a month 5. A few times a year 6. None

8) How often do you meditate or pray at home? 【per6pr_18】
1. Four or more a week 2. Two or three times a week 3. Once a week

4. One to three times a month 5. A few times a year 6. None

9) Overall, how important would you say religion is in your life? 【per6im_18】
1. Not at all important 2. Slightly important 3. Moderately important 4. Very important
5. I don't know 6. I don't want to answer

10) To what degree do you feel you are currently happy? (Score "0" for "Very unhappy" and "10" for "Very happy.") 【happy10_18】

Very unhappy	Very Happy
0----------1----------2----------3----------4----------5----------6----------7----------8----------9----------10	

11) How long have you been living in your present township?

About	【rsdyrz_18】	years	【rsdmth_18】	months

12) In the past five years, have you ever moved to or from children's or relatives' house? 【mvhs4_18】
1. No 2. Once or twice 3. Three to five times 4. Six times or more

13) Please circle the highest educational level you have been below? (Single answer)
1. No school 【educ2no_18】 2. Monastic education (only read and write) 【educ2me_18】 3. Some primary 【educ2sp_18】 4. Finished primary 【educ2fp_18】
5. Middle school 【educ2ms_18】 6. High school 【educ2hs_18】 7. Vocational 【educ2vo_18】 8. College/university 【educ2co_18】

14) Have you experienced any of the following events over the past year? Circle the numbers of all answers that apply.
1. I started a new job. 【evnt2gj18】 2. I quit my job or retired. 【evnt2rs18】 3. I started living with my children. 【evnt2cc18】
4. I started living alone. 【evnt2al18】 5. I became better off financially. 【evnt2er18】 6. I became worse off financially. 【evnt2fd18】
7. A new grandchild or great-grandchild was born. 【evnt2ch18】 8. I lost my spouse. 【evnt2sd18】
9. A family member or close friend or relative passed away. 【evnt2cd18】 10. I acquired new friends. 【evnt2fr18】
11. I suffered a serious illness. 【evnt2di18】 12. I started caring for sick family members. 【evnt2cr18】 13. Other (   ) 【evnt2ot18】
14. No major changes. 【evnt2no18】

Q19. The following questions are about your family.

1) What is your marital status? 【mari5st18】
1. Married 2. Widowed 3. Divorced 4. Never married 5. Other

2) Do you have child(ren)? 【chld3n18】
1. Yes, and all (or some) are alive. 2. Yes, but all passed away 3. No, I don't have any child(ren).

3) Which of the following best describes your family composition? 【alone318】
1. I live alone 2. I live with my family (blood-related) 3. I live with other family (not blood-related, friend, subordinate family, etc.) 4. Other (e.g. institution, Home for Age, common shelter)

4) How many people are in your household, including yourself? 【mebr2nb18】

| | people

5) Who do you live with? Circle all that apply.
1. None 2. Spouse 3. Son(s) 4. Daughter(s) 5. Spouse(s) of child(ren) 6. Grandchild(ren)
【hous2no18】【hous2sp18】【hous2so18】【hous2da18】【hous2cs18】【hous2gc18】
7. Brother(s) or sister(s) 8. My father 9. My mother 10. My father-in-law
【hous2sb18】 【hous2fa18】【hous2mo18】【hous2lf18】
11. My mother-in-law 12. Other (【hous2ottxt18】)
【hous2lm18】 【hous2ot18】

6) What is the average total income for your household from all sources in a normal month?
1. Kyat (【hhinem_18】) 2. I don't know【hhinemidn_18】

7) Which of the following best describes your current financial situation in light of general economic conditions? 【sfs5_18】
1. Very difficult 2. Difficult 3. Average 4. Comfortable 5. Very comfortable

8) What do you think of your living condition at your child age in light of social average around you? 【ssl15_5_18】
1. Very good 2. Good 3. Normal 4. Bad 5. Very bad

9) Do you have sources of drinking water that required no more than 30 minutes per trip to collect water? 【srcwt2_18】
1. Yes 2. No

10) Do you use the following facilities which are not shared with other households?
1. Flush/pour flush toilets connecting to sewers, septic tanks or latrines 1. Yes 2. No
【factoi2_18】
2. Ventilated pit latrines, pit latrines with slabs, or composting toilets 1. Yes 2. No
【faclat2_18】

11) Does your household have mosquito nets for each household member? 【net3_18】
1. Yes 2. There are mosquito nets in household, but the number is not sufficient
3. There is no mosquito net in household

Q20. The following questions are about your past and current employment.

1) What is the type of the occupation that you have taken the longest in your life? 【empl9lgst18】
1. Professional/Technical 2. Managerial 3. Clerical 4. Sales/service 5. Skilled Labor
6. Agriculture, forestry or fisheries 7. Self-employment other than agriculture, forestry, and fisheries
8. Other (【empl9lgsttxt18】) 9. I have never had a job

2) What is your current employment status? 【empl3pt18】
1. I am employed. 2. I am retired from my job. 3. I have never had a job.

3) If you chose "2" or "3" in the above, are you currently looking for work? 【empl2sk18】
1. Yes 2. No

Q21. The following questions are asking about your permanent and durable properties.

Do you or any member of your household have the following items?

Radio【hv2ra_18】	1. Yes 2. No	Washing machine【hv2wm_18】	1. Yes 2. No
Black & white TV【hv2mt_18】	1. Yes 2. No	Gas cooker【hv2gc_18】	1. Yes 2. No

Color TV【hv2ct_18】	1. Yes 2. No		Electric cooker or rice cooker【hv2ec_18】	1. Yes 2. No
Video/DVD player【hv2vd_18】	1. Yes 2. No		Air conditioner【hv2ac_18】	1. Yes 2. No
Electric fan【hv2ef_18】	1. Yes 2. No		Bicycle【hv2bi_18】	1. Yes 2. No
Refrigerator【hv2rf_18】	1. Yes 2. No		Motorcycle【hv2mo_18】	1. Yes 2. No
Computer【hv2pc_18】	1. Yes 2. No		Car/truck【hv2ca_18】	1. Yes 2. No
Store bought furniture【hv2fu_18】	1. Yes 2. No		Microwave oven【hv2ov_18】	1. Yes 2. No
Personal music player【hv2mp_18】(Cassette, MP3 etc)	1. Yes 2. No		Mobile telephone【hv2te_18】	1. Yes 2. No
			Internet【hv2in_18】	1. Yes 2. No

Q22. The following questions are about your treatment by family members.
If someone is present around the respondent please tell him/ her to leave for a while

1) In the past year, did you ever experience physical violence from your family, such as being hit, kicked, having objects thrown at you, or being shut in a room. 【abus2phf18】
 1. Yes 2. No

2) In the past year, did you ever experience an act to harm your self-esteem from your family, such as verbal abuse, cutting remarks, or being ignored for long periods. 【abus2psf18】
 1. Yes 2. No

3) Do any of your family members take or use your money without your consent? 【abus2fnf18】
 1. Yes 2. No

Q23. The following questions are about your future.

1) When you need long-term care due to functional/cognitive decline and/or any disability, do you expect someone to care for you? 【ftr3ltc18】
1. Yes 2. No 3. I don't know

2) If you answer "1. Yes" to the question before (Q23-1), who do you expect to care for you? Please, circle all possible item(s).
1. Spouse【ftr2sp18】 2. Child(ren)【ftr2ch18】 3. Child(ren) in law【ftr2chl18】
4. Brother/sister【ftr2bs18】 5. Relative(s)【ftr2rl18】 6. Friend(s) 【ftr2fr18】
7. Neighbor(s) 【ftr2nb18】8. Other【ftr2ot18】(【ftr2ottxt18】)
