# Supplementary material for: Association between depressive symptoms and objective/subjective socioeconomic status among older adults of two regions in Myanmar
Source: PLoS One. 2021 Jan 28;16(1):e0245489. doi: 10.1371/journal.pone.0245489 (PMC7842968; doi:10.1371/journal.pone.0245489)
Supplement: Attachment — Submitted filename: Response to Editors and Reviewers (R1).docx [file pone.0245489.s003.docx]

**Response to Editor and Reviewers**

Re: PONE-D-20-15915

Association between depressive symptoms and objective/subjective socioeconomic status among older **adults of two regions in** Myanmar

**Response to editors**

We wish to thank the editor for giving us helpful comments regarding our manuscript. We have revised our manuscript accordingly.

**Editor comment 1:**

A rebuttal letter that responds to each point raised by the academic editor and reviewer(s). You should upload this letter as a separate file labeled 'Response to Reviewers'.

A marked-up copy of your manuscript that highlights changes made to the original version. You should upload this as a separate file labeled 'Revised Manuscript with Track Changes'.

An unmarked version of your revised paper without tracked changes. You should upload this as a separate file labeled 'Manuscript'.

**Our reply 1**

We wish to thank the editor for giving us the comments. We have uploaded ‘Response to Editors and Reviewers’, ‘Revised Manuscript with Track Changes’, and ‘Manuscript’, separately.

**Editor comment 2:**

If applicable, we recommend that you deposit your laboratory protocols in protocols.io to enhance the reproducibility of your results. Protocols.io assigns your protocol its own identifier (DOI) so that it can be cited independently in the future.

**Our reply 2**

We thank the editors for this suggestion. We submitted a paper the regarding cohort profile for this study, and it has been under review.

**Editor comment 3:**

Please ensure that your manuscript meets PLOS ONE’s style requirements, including those for file naming.

**Our reply 3**

We thank the editors for this requirement. We confirmed it.

**Editor comment 4:**

Please include additional information regarding the survey or questionnaire used in the study and ensure that you have provided sufficient details that others could replicate the analyses. For instance, if you developed a questionnaire as part of this study and it is not under a copyright more restrictive than CC-BY, please include a copy, in both the original language and English, as Supporting Information.

**Our reply 4**

We have added the questionnaire in both the original language (Burmese) and English, as Supporting Information.

**Editor comment 5:**

Please state whether you validated the questionnaire prior to testing on study participants. Please provide details regarding the validation group within the methods section.

**Our reply 5**

We thank the editors for this fruitful suggestion. We have added the following information in the Method section.

**“The questionnaire was first developed in English. After being translated into Burmese (dominant language), these were back translated to English to ensure clarity and consistency.**

**We hired research staff from the Myanmar Perfect Research Company, a group that conducts epidemiological surveys in Myanmar. The interviewers were recruited from the company. Before the commencement of the actual survey, a two-day training course on the research protocol, administration of the questionnaire, and ethical concerns was conducted for the interviewers.**

**A small pilot study was carried out before the actual survey for face validity in Urban Health Center, Dagon township, Yangon. Participants were the older adults, age of above 60 came to the out-patient clinic in the center. We recruited the 25 respondents who were gave consent to participate in the pilot study, during time of pilot data collection in June 2018. During the pilot study, the interviewers ensured sequence, flow, and clarity of the study. After the feedback from the interviewers, the questionnaire was revised accordingly.”**

**Editor comment 6:**

We note that you have indicated that data from this study are available upon request. PLOS only allows data to be available upon request if there are legal or ethical restrictions on sharing data publicly. For more information on unacceptable data access restrictions, please see http://journals.plos.org/plosone/s/data-availability#loc-unacceptable-data-access-restrictions.

・In your revised cover letter, please address the following prompts:

a) If there are ethical or legal restrictions on sharing a de-identified data set, please explain them in detail (e.g., data contain potentially sensitive information, data are owned by a third-party organization, etc.) and who has imposed them (e.g., an ethics committee). Please also provide contact information for a data access committee, ethics committee, or other institutional body to which data requests may be sent.

b) If there are no restrictions, please upload the minimal anonymized data set necessary to replicate your study findings as either Supporting Information files or to a stable, public repository and provide us with the relevant URLs, DOIs, or accession numbers. For a list of acceptable repositories, please see <http://journals.plos.org/plosone/s/data-availability#loc-recommended-repositories>.

**Our reply 6**

Thank you for your useful information. An ethics committee has placed an ethical restriction on sharing de-identified data, since the data may contain sensitive information regarding the respondents’ physical and mental health. The following is the contact information of the individuals responsible for the dataset.

Ethical Review Committee of the National Institute of Public Health, Japan

TEL +81-48-458-6111

FAX +81-48-469-1573

2-3-6 Minami, Wako City, Saitama, Japan, 351-0197

**Editor comment 7:**

PLOS requires an ORCID iD for the corresponding author in Editorial Manager on papers submitted after December 6th, 2016.

**Our reply 7:**

Thank you for this information. I, the corresponding author had the ORCID iD: <https://orcid.org/0000-0002-5832-7001>

**Responses to the Reviewers**

Thank you for your thorough consideration of our manuscript and for providing constructive feedback.

We highlighted all of our changes by underlining the new parts in the revised manuscript.

**Reviewer 1 comments:**

**Reviewer1-1 comment:**

Line 24: ‘It influences mental health through adverse effects on physical surroundings and psychosocial experiences.’ This suggests a causal relationship, which I don’t think can be made. An action is being attributed to inequalities that are not animate, and even then the causal pathways are much more complicated than described. This confusion may result from translation from the authors own language to English?

**Our reply 1-1:**

We thank the reviewer for this valuable comment.

We have revised the sentence as follows:

**“Low objective SES (e.g., household wealth; working and poverty status) has been correlated with poor physical and mental health”.**

**Reviewer1-2 comment:**

Line 26: ‘However, no studies have been conducted on the impact of both SES on depressive symptoms in older people.’ This sentence is not particularly clear, it would be better to say ‘both objective and subjective SES’ to be clear. Moreover, a quick search of the Web of Science identified handful of papers already published on this topic, and therefore I don’t think the sentence is correct. The distinctive effects of objective and subjective socioeconomic status have been recognised for quite a few years and have been the focus of significant research. Depression might not been examined specifically in each of these papers, but as the authors note in the discussion their questionnaire would not necessarily distinguish diagnosed cases of depression, and therefore the outcome might be identifying life satisfaction as much as depression. Here are the DOI of some of the papers I found:

• 10.1016/j.archger.2016.07.006

• 10.1186/1471-2458-11-166

• 10.1016/j.socscimed.2008.03.038

• 10.1016/j.socscimed.2006.06.025

• 10.1111/j.1532-5415.2005.53169.x

Further development of the introduction and discussion are needed to represent this wider body of literature. Myanmar seems to be a novel study location, and therefore the low-income setting is more unusual, but great inclusion of the previous literature is needed.

**Our reply 1-2:**

Thank you for this important comment. We have revised the sentence following your comments and added the following to the Introduction and Discussion sections.

**(Abstract)**

“However, no studies have been conducted on the impact of both objective and subjective SES on depressive symptoms in older adults **in Myanmar.”**

**(Introduction)**

**“Additionally, subjective social status (SSS), which is defined as a person’s perceived rank relative to others in their group, and concept similar to subjective SES (15) have been found to mediate the associations between objective SES and depression (16). Moreover, some studies have found associations between objective/subjective SES and health measures such as self-rated health and life satisfaction among older adults in Korea (17) and Taiwan (18). One meta-analysis revealed a significant independent association between the subjective SES and physical health in adults, beyond traditional objective indicators of SES such as education, occupation, and income (19). Therefore, both objective and subjective SES are hypothesized to be one of the most significant disparities, where physical and psychological health are poor among individuals of lower SES. However, this relationship does not seem to simply reflect the effects of poverty (19). Evidence for the association between SES (measured in various forms) and health has been interpreted as evidence that social stratification, not simply objective socioeconomic resources, have a meaningful impact on physical health (19, 20).**

However, **there are few studies** regarding the effects of objective/subjective SES on depressive symptoms among older adults in developing countries **including Myanmar.** However, depressive symptoms are common among older adults as the aging population grows, especially in Asian countries (21), and its socioeconomic cost is vast due to **higher rates of morbidity and mortality and increased healthcare utilization and economic cost compared to younger adults (22).”**

**“However, effective medical care systems including mental health services are still underdeveloped. Few studies regarding mental health for older adults have been published** due to the country’s history of military control from 1962 to 2011 **and ongoing conflicts in Myanmar’s ethnic areas (31-33).”**

**(Discussion)**

**“**The main contribution of this study was the identification of the associations between depressive symptoms and objective and subjective SES in older adults **living in low-income settings in Myanmar, where population aging is projected to increase rapidly.”**

**Reviewer1-3 comment:**

Line 91: ‘In rural areas, some villages exist under the village tract.’ I think the sentence is meant to say that there can be multiple villages within a single village tract.

**Our reply 1-3:**

Thank you for this comment. It is precisely as you said.

We have revised it following your comment.

**“In rural areas,** **there are multiple villages within a single village tract.”**

**Reviewer1-4 comment:**

Line 96: ‘we considered Yangon as representative of urban areas and Bago as representative of rural areas.’ Additional information is needed in order to justify this sentence, the authors opinion is not sufficient. More broadly speaking more information is needed comparing the sample with the population to explore any possibly biases. As it stands we do not have any way of assessing whether the findings might just relate to a biased sample, or might relate to a wider problem across Myanmar?

**Our reply 1-4:**

Thank you for this comment.

We have added the followings in the method section and limitation section.

**Method section**

**“The target population were those in the urban area of the Yangon region and the rural area of the Bago region 91 kilometers northeast of Yangon. The Republic of the Union of Myanmar is composed of seven regions and seven states. Given that this survey was conducted in only two regions, it may not have been representative of older adults in all of Myanmar.”**

**“**In this survey, we selected only wards from Yangon and only village tracts from Bago **to capture the features of urban and rural areas from each region;** we considered Yangon as representative of urban areas and Bago of rural areas**.”**

**Limitation section**

**“Second,** it is unknown whether these findings are generalizable beyond Yangon and Bago. People living in Bago could enjoy greater access to urban areas and be more likely to access health facilities relative to those living in other rural areas of the country. **Therefore, it is difficult to generalize the study findings to the population in areas where access to urban areas and health services are limited. However, we can estimate situations of older adults in other regions by the level of urbanization of the selected regions. Ideally,** this survey should be extended to include all surrounding regions in the future.**”**

**Reviewer1-5 comment:**

Line 260: ‘studies should be needed as to what types of social supports and human interaction are’. The English in this sentence is not great, I think you could talk about further studies being needed…

**Our reply 1-5:**

Thank you for your suggestion. We have revised the sentence as follows.

**“**Although we adjusted for instrumental social support, which was associated with depressive symptoms in a bivariate analysis, **further studies are needed to examine the association between types of social support and human interactions and depressive symptoms in Myanmar.”**

**Reviewer1-6 comment:**

It would be useful to know something about the order in which the questions were asked, as this could have influenced the responses received. If the depression questions led straight onto the subjective socioeconomic status questions, the participant’s mindset might have led to poorer responses.

**Our reply 1-6:**

Thank you for this comment. We have added the questionnaire which we used to obtain the “supporting information” and added the following information in the Method section.

**“****A 14-page s**tructured questionnaire—**based on a questionnaire used the “Japan Gerontological Evaluation Study (JAGES)(34)”—**was developed for face-to-face interviews. **JAGES was established in 2010 as a nationwide, population-based prospective cohort study for older community-dwelling Japanese adults. The linguistic translation and validation process followed the “Linguistic Validation Manual for Health Outcome Assessments (35).” The questionnaire was first developed in English. After being translated into Burmese (dominant language), these were back translated to English to ensure clarity and consistency.**

**///**

**A small pilot study was carried out before the actual survey for face validity in Urban Health Center, Dagon township, Yangon. Participants were the older adults, age of above 60 came to the out-patient clinic in the center. We recruited the 25 respondents who were gave consent to participate in the pilot study, during time of pilot data collection in June 2018. During the pilot study, the interviewers ensured sequence, flow, and clarity of the study. After the feedback from the interviewers, the questionnaire was revised accordingly. To avoid the question order bias, we have placed questions about depressive symptoms away from question about subjective socioeconomic status (see supporting information).”**

**Reviewer1-7 comment:**

It would be good to give some insight into whether the collection of data in 2018 might have impacted the findings. An international recession began in 2018, might this have impacted on findings?

**Our reply 1-7:**

Thank you for this comment.

Given that this is a cross-sectional study, we believe it may be difficult to evaluate the impact of international recession. Additionally, the Gross Domestic Product (GDP) in Myanmar was worth US$ 76.17 billion in 2018, according to official data from the World Bank (please see Figure a below). The distribution of household income also increased between 2000–2018. The proportion of the middle-income group (household income of US$ 5,000 to 34,999 US$) rose from about 1.2% in 2000 to 20.6% in 2018. In particular, the proportion of upper middle-income individuals (US$ 10,000 to 34,999) is increasing (please see Figure b) below. From these data, we expect that the international recession that began in 2018 has not had a significant influence on our findings.

Source: Tradingeconomics.com, World Bank

Figure a. Gross Domestic Product (GDP) in Myanmar

Source: Euromonitor International

Figure b. Distribution of household income in Myanmar

**Reviewer1-8 comment:**

As an epidemiological study I think the reporting needed to be improved to provide information about bias and aid the understanding of external validity. Using a reporting guideline like the STROBE checklist would ensure that all the relevant details are reported.

**Our reply 1-8:**

Thank you for this comment. We have added the followings in Discussion section.

**“The assessment based on self-report may have caused social desirability bias, resulting in misreporting of depressive symptoms.”**

**“Second,** it is unknown whether these findings are generalizable beyond Yangon and Bago. People living in Bago could enjoy greater access to urban areas and be more likely to access health facilities relative to those living in other rural areas of the country. **Therefore, it is difficult to generalize the study findings to the population in areas where access to urban areas and health services are limited. However, we can estimate situations of older adults in other regions by the level of urbanization of the selected regions. Ideally,** this survey should be extended to include all surrounding regions in the future.**”**

**Reviewer1-9 comment:**

There are a large number of statistical tests undertaken for the sample size, given that models for urban/rural and gender are presented. Therefore, some discussion of the potential risk of false positives from the multiple testing is needed. Also, there is some evidence of very wide confidence intervals which suggests small numbers of participants in those groups. The assumptions of the models should be tests to ensure outliers are not overly impacting on the results. A sentence reporting that no breeches of assumptions were found, should be included in the paper.

**Our reply 1-9:**

Thank you for this comment.

Given that we only used categorical variables, the influence of outliers is expected to be small. However, we have added the following in the limitation section.

**“Fourth, there were several statistical tests undertaken for the sample size, and there were wide confidence intervals after stratification by sex and region. Therefore, there is a potential risk of false positives from the multiple testing and of decreasing accuracy after the stratification.”**

**Reviewer1-10 comment:**

The authors report that the study data are available upon request, which does not match their response that the data are fully available. I completely understand the restrictions around this sensitive data, but the journal gives the author space to explain that the data are only available in specific circumstances.

**Our reply 1-10:**

Thank you for pointing out. We have revised the report that this data are only available in specific circumstances following the instruction from the PLOS ONE editor.

**Reviewer 2 comments:**

**Reviewer2-1 comment:**

The authors should briefly explain the situation in the mental health care system in Myanmar’s urban and rural areas in the introduction section. It is essential to understand “social support” available in the country at the time of this study. Information on the role of religious facilities in Myanmar’s daily life should also be added.

**Our reply 2-1:**

We thank the reviewer for this fruitful suggestion. We have added the following information in the Introduction section.

**“The 2017 Global Burden of Disease study reports that in Myanmar both depressive and anxiety disorders are among the top 10 contributors to years lived with disability and have increased over the past decade (24). Studies in Myanmar have found that depressive symptoms in older adults were strongly associated with less activities of daily living and lower quality of life (25) and economic and health status (26). Several health organizations provide community-based health care services even in remote areas and are seeking to coordinate health service provision with the central health care system (27). Religious organizations are also involved in service provision, and their roles are gaining value with the increasing need for collaborative action in health (28). Certain studies focusing on the effects of Vipassana mediation indicated that participants in the mediation courses reported reductions in anxiety and stress (29, 30). However, effective medical care systems including mental health services are still underdeveloped. Few studies regarding mental health for older adults have been published** due to the country’s history of military control from 1962 to 2011 **and ongoing conflicts in Myanmar’s ethnic areas (31-33)”**

**Reviewer2-2 comment:**

Lines 101–129

The authors need to reconstruct sections between Study tools and Confounding variables. All the study tools should be mentioned in the Study tools section.

**Our reply2-2**

Thank you for this suggestion. We revised the subheadings as follows

**Study tool (1) Questionnaire; Study tool (2) Dependent variable; Study tool (3) Independent variables; Study tool (4) Confounding variables**

**Reviewer2-3 comment:**

What was first translated into English in line 103? “Japan Gerontological Evaluation Study” is the name of a tool?

**Our reply2-3**

Thank you for this question. We have added the following information.

**“A 14-page** structured questionnaire**—based on a questionnaire used the “Japan Gerontological Evaluation Study (JAGES)(34)”—was developed for face-to-face interviews. JAGES was established in 2010 as a nationwide, population-based prospective cohort study for older community-dwelling Japanese adults. The linguistic translation and validation process followed the “Linguistic Validation Manual for Health Outcome Assessments (35).” The questionnaire was first developed in English. After being translated into Burmese (dominant language), these were back translated to English to ensure clarity and consistency.”**

**Reviewer2-4 comment:**

The authors also need to briefly explain what “abbreviated mental test” is like, including scoring and evaluation information.

**Our reply2-4**

Thank you for this suggestion. We have added the questionnaire as “supporting information” and the following sentences and references.

“We assessed depressive symptoms using the 15-item version of the Geriatric Depression Scale (GDS), which was validated previously (38-41). The GDS involves a simple yes/no format **(see supporting information Q17 1)-15)) that is easy to administer and score (42, 43). Participants were classified into two groups: no depressive symptom (GDS score < 5), and having depressive symptoms (GDS score ≥ 5) (39, 44-46).”**

**Reviewer2-5 comment:**

When the authors use any evaluation tools in a different language for the very first time, it is essential to show the linguistic translation and validation process. The explanation in this manuscript is too vague. The authors should refer to Linguistic validation manual for health outcome assessments (2012, MAPI Institute) or other previous validation studies with the translation process on how to carry out translation and validation procedures to create an evaluation tool in a different language. The authors need to show this process in detail and mention the insufficiency, if any, in the limitation section. The same process needs to be applied to GDS and the wealth index as well.

**Our reply2-5**

Thank you for this comment. We have added the followings.

“**The linguistic translation and validation process followed the “Linguistic Validation Manual for Health Outcome Assessments (35).” The questionnaire was first developed in English. After being translated into Burmese (dominant language), these were back translated to English to ensure clarity and consistency. ///**

**A small pilot study was carried out before the actual survey for face validity in Urban Health Center, Dagon township, Yangon. Participants were the older adults, age of above 60 came to the out-patient clinic in the center. We recruited the 25 respondents who were gave consent to participate in the pilot study, during time of pilot data collection in June 2018. During the pilot study, the interviewers ensured sequence, flow, and clarity of the study. After the feedback from the interviewers, the questionnaire was revised accordingly. To avoid the question order bias, we have placed questions about depressive symptoms away from question about subjective socioeconomic status (see supporting information).”**

**Reviewer2-6 comment:**

Lines 225–227

The authors should refer to any previous articles or data on “which has been considered an egalitarian society with relatively few inequalities in health” to state that this comment is not just the authors’ personal belief.

**Our reply2-6**

Thank you for this comment.

We have added 2 references and revised the sentence as follows.

**“Even in Japan, considered an egalitarian society—as reflected by a Gini coefficient of 63% in 2019 (54)—with relatively few inequalities in health (55),** substantial social inequalities in mental health, measured by SSS, were identified (11).”

**Reviewer2-7 comment:**

Lines 238–

Bonding social capital was not directly questioned in this research. This should be mentioned in the limitation section.

**Our reply2-7**

Thank you for this comment. We have mentioned “Bonding social capital” in the limitation section as follows.

**“Fifth, indicators for bonding social capital, which is derived from relationships between similar persons such as with respect to sociodemographic and socioeconomic characteristics, were not included in our analysis./**// This may also increase the risk of depressive symptoms in rural older adults. Although we adjusted for instrumental social support, which was associated with depressive symptoms in a bivariate analysis, **further studies are needed to examine the association between types of social support and human interactions and depressive symptoms in Myanmar.”**

**Reviewer2-8 comment:**

Lines 273–274

The authors referred to the paper by Sommanustweechai et al. (2016)(45), but this previous study does not mention GDS validation. The papers by Yesavage et al. (1982) Sasaki et al. (2019) can not confirm linguistic translation and validation in Burmese language and Burmese cultural background.

**Our reply2-8**

Thank you for this point.

As mentioned in “Our reply1-6,” we added the process of linguistic translation and validation as follows. We have also added references which mention GDS validation.

**“A 14-page** structured questionnaire—**based on a questionnaire used the “Japan Gerontological Evaluation Study (JAGES)(34)”**—was developed for face-to-face interviews. **JAGES was established in 2010 as a nationwide, population-based prospective cohort study for older community-dwelling Japanese adults. The linguistic translation and validation process followed the “Linguistic Validation Manual for Health Outcome Assessments (35).” The questionnaire was first developed in English. After being translated into Burmese (dominant language), these were back translated to English to ensure clarity and consistency.////**

**A small pilot study was carried out before the actual survey for face validity in Urban Health Center, Dagon township, Yangon. Participants were the older adults, age of above 60 came to the out-patient clinic in the center. We recruited the 25 respondents who were gave consent to participate in the pilot study, during time of pilot data collection in June 2018. During the pilot study, the interviewers ensured sequence, flow and clarity of the study. After the feedback from the interviewers, the questionnaire was revised accordingly. To avoid the question order bias, we have placed questions about depressive symptoms away from question about subjective socioeconomic status (see supporting information).”**

“We assessed depressive symptoms using the 15-item version of the Geriatric Depression Scale (GDS), which was validated previously (38-41). The GDS involves a simple yes/no format **(see supporting information Q17 1)-15)) that is easy to administer and score** (42, 43). **Participants were classified into two groups: no depressive symptom (GDS score < 5), and having depressive symptoms (GDS score ≥ 5) (39, 44-46)”**

**“40. Zalsman G, Aizenberg D, Sigler M, Nahshoni E, Weizman A. Geriatric depression scale-short form–validity and reliability of the Hebrew version. Clinical Gerontologist. 1998;18(3):3-9.**

**41. Sheikh JI, Yesavage JA. Geriatric Depression Scale (GDS): recent evidence and development of a shorter version. Clinical Gerontologist: The Journal of Aging and Mental Health. 1986.”**

**Reviewer2-9 comment:**

Line 160 and other

There are some vague expressions such as “These were ...” in line 160.

**Our reply2-9**

Thank you for this comment.

We have revised the sentence as follows

**“Both experiences of illness during the preceding year and no school** were significantly higher among those who had depressive symptoms (64.9% and **10.6%, respectively)** than those who did not **have depressive symptoms** (47.3% and **8.0%, respectively**).”

**Reviewer2-10 comment:**

Line 274

“Addition” should be “In addition” or “Additionally” in line 274.

**Our reply2-10**

Thank you for this comment.

We have revised it as follows based on the reviewer1-9 comment.

“**Second,** it is unknown whether these findings are generalizable beyond Yangon and Bago.”

**Reviewer 3 comments:**

**Reviewer3-1 comment:**

Abstract

In the abstract, in this statement “no studies have been conducted on the impact of both SES on depressive symptoms in older people”, please specify where is?

**Our reply 3-1:**

Thank you for this comment.

We have added the site as follows.

**“**However, no studies have been conducted on the impact of both objective and subjective SES on depressive symptoms in older adults **in Myanmar.”**

**Reviewer3-2 comment:**

Introduction

1. Please elaborate on the contextual factors affecting depression and the status of socio-economic condition in elder Myanmar population.

**Our reply 3-2:**

Thank you for this comment. There are few studies regarding the contextual factors in Myanmar. However, we have added the following.

**“The 2017 Global Burden of Disease study reports that in Myanmar both depressive and anxiety disorders are among the top 10 contributors to years lived with disability and have increased over the past decade (24). Studies in Myanmar have found that depressive symptoms in older adults were strongly associated with less activities of daily living and lower quality of life (25) and economic and health status (26). Several health organizations provide community-based health care services even in remote areas and are seeking to coordinate health service provision with the central health care system (27). Religious organizations are also involved in service provision, and their roles are gaining value with the increasing need for collaborative action in health (28). Certain studies focusing on the effects of Vipassana mediation indicated that participants in the mediation courses reported reductions in anxiety and stress (29, 30). However, effective medical care systems including mental health services are still underdeveloped. Few studies regarding mental health for older adults have been published** due to the country’s history of military control from 1962 to 2011 **and ongoing conflicts in Myanmar’s ethnic areas (31-33).**”

**Reviewer3-3 comment:**

Introduction

2. More cogent and clear arguments need to be made as to why the associations were investigated between objective, subjective social economic status and depressive symptoms.

**Our reply 3-3:**

Thank you for this comment. We have added the followings

**“In addition, subjective social status (SSS), which is defined as a person’s perceived rank relative to others in their group, and similar concept to subjective SES (15), has been found to mediate the associations between objective SES and depression (16). Moreover, there are a few studies which have found the association between objective/subjective SES and health measures such as self-rated health and life satisfaction among older adults in Korea (17) and Taiwan (18). One meta-analysis revealed a significant independent association between the subjective SES and physical health in adults, above and beyond traditional objective indicators of SES such as education, occupation, and income (19). Thus, both objective and subjective SES are hypothesized to be one of the most significant disparities, where physical and psychological health condition is poor among individuals of lower SES. However, this relationship does not seem to simply reflect the effects of poverty (19). Evidence for the association between SES (measured in various forms) and health has been interpreted as evidence that social stratification, not simply objective socioeconomic resources, has a meaningful impact on physical health (19, 20).**

However, there are no previous studies about whether objective/subjective SES have a greater effect on older adults’ depressive symptoms in developing countries including Myanmar, although their depressive symptoms are common as the aging population grows especially in Asian countries (21), and its socioeconomic cost is vast due to **higher rates of morbidity and mortality, increased healthcare utilization and economic cost in comparison to younger adults (22).**”

**“Additionally, subjective social status (SSS), which is defined as a person’s perceived rank relative to others in their group, and concept similar to subjective SES (15) have been found to mediate the associations between objective SES and depression (16). Moreover, some studies have found associations between objective/subjective SES and health measures such as self-rated health and life satisfaction among older adults in Korea (17) and Taiwan (18).** **One meta-analysis revealed a significant independent association between the subjective SES and physical health in adults, beyond traditional objective indicators of SES such as education, occupation, and income (19). Therefore, both objective and subjective SES are hypothesized to be one of the most significant disparities, where physical and psychological health are poor among individuals of lower SES. However, this relationship does not seem to simply reflect the effects of poverty (19). Evidence for the association between SES (measured in various forms) and health has been interpreted as evidence that social stratification, not simply objective socioeconomic resources, have a meaningful impact on physical health (19, 20).**

However, there are few studies regarding the effects of objective/subjective SES on depressive symptoms among older adults in developing countries **including Myanmar.** However, depressive symptoms are common among older adults as the aging population grows, especially in Asian countries (21), and its socioeconomic cost is vast due to **higher rates of morbidity and mortality and increased healthcare utilization and economic cost compared to younger adults (22).”**

**Reviewer3-4 comment:**

Methods:

1. The study design that you mentioned in line 85, 86 and 87 is rather vague. Please clearly specify your study design and time period.

**Our reply 3-4:**

Thank you for this comment. We have revised the sentence as follows.

“This was **a cross-sectional baseline survey** for a longitudinal study. It **was conducted between September and December 2018 in Myanmar** and examined the predictors of physical and psychological health in **1,200** community-dwelling older adults aged ≥ 60 years.

”

**Reviewer3-5 comment:**

Methods:

2. Reason as to why Yangon and Bago region was chosen seem tenuous. There is also rural areas in Yangon region why do you choose Bago region as rural (area).

**Our reply 3-5:**

Thank you for this comment. We have described our target population more clearly and added the following. In addition, we have revised the title in this study.

**“The target population were those in the urban area of the Yangon region and the rural area of the Bago region 91 kilometers northeast of Yangon. The Republic of the Union of Myanmar is composed of seven regions and seven states. Given that this survey was conducted in only two regions, it may not have been representative of older adults in all of Myanmar.”**

**“The difference between a ward and village tract is the degree of urbanization.** The ward is the minimum unit of a residential district in an urban area, and the village tract is the corresponding level in rural areas. However, wards and village tracts sometimes co-exist within a township. In this survey, we selected only wards from Yangon and only village tracts from Bago **to capture the features of urban and rural areas from each region;** we considered Yangon as representative of urban areas and Bago of rural areas.”

**“**Association between depressive symptoms and objective/subjective socioeconomic status among older **adults of two regions** in Myanmar**”**

**Reviewer3-6 comment:**

Methods:

3. In your abstract, you described multi-stage sampling so you need to mentioned detail sampling procedure.

**Our reply 3-6:**

Thank you for this comment. We have added the following information.

**“****The target population was the urban area of the Yangon region, and the rural area of the Bago region located 91 kilometers north-east of Yangon. The Republic of the Union of Myanmar is composed by seven regions and seven states. Since this survey was conducted only in the two regions, it could not be representative sample of older adults in whole Myanmar nation.**

**Multistage random sampling was conducted in the two regions. There are 45 townships in the Yangon region, and 28 townships in the Bago region. First,** six townships were randomly selected from each region via population proportionated sampling **based on the population of each township. Next,** in Yangon, 10 wards were further randomly selected from each township, while in Bago, 10 village tracts were selected from each township again via population proportionated sampling based on the population of each township/village tract. **Finally, 10 people were randomly selected from each extracted ward/village tract using the ledger lists of the residents aged 60 years or more. In rural areas, there are multiple villages within a single village tract.** In such cases, one of the villages was randomly selected from the village tract.

**Difference between ward and village tract is in degree of urbanization in Myanmar.** The ward is the minimum unit of a residential district in an urban area, and the village tract is the corresponding level in rural areas. However, wards and village tracts sometimes co-exist within a township. In this survey, we selected only wards from Yangon and only village tracts from Bago **to capture the feature of urban areas and rural areas from each region:** we considered Yangon as representative of urban areas and Bago as representative of rural areas.

Trained surveyors visited homes with a public health nurse to meet participants. **In Yangon, the surveyors visited 1,083 older adults and 610 were at home. Ten were excluded due to refusing the survey (n=6), and severe dementia or bed ridden (n=4), thus response rate was 98.4% in Yangon. In Bago, surveyors visited 1044 older adults and 694 were at home. Ninety-four were excluded due to severe dementia or bed-ridden, thus the response rate was 86.5% in Bago. In total, six hundred from Yangon region (222 men and 378 women), and also six hundred from Bago region (261 men and 339 women) were finally surveyed.**”

**“The target population were those in the urban area of the Yangon region and the rural area of the Bago region 91 kilometers northeast of Yangon. The Republic of the Union of Myanmar is composed of seven regions and seven states. Given that this survey was conducted in only two regions, it may not have been representative of older adults in all of Myanmar.**

**Multistage random sampling was conducted in the two regions. There are 45 townships in the Yangon region and 28 in the Bago region. First,** six townships were randomly selected from each region via population proportional sampling **based on the population of each township. Next,** in Yangon, 10 wards were further randomly selected from each township, while in Bago, 10 village tracts were selected from each township again based on the population of each township/village tract. **Finally, 10 people were randomly selected from each extracted ward/village tract using the ledger lists of residents aged 60 years or more. In rural areas, there are multiple villages within a single village tract.** In such cases, one of the villages was randomly selected from the village tract.

**The difference between a ward and village tract is the degree of urbanization.** The ward is the minimum unit of a residential district in an urban area, and the village tract is the corresponding level in rural areas. However, wards and village tracts sometimes co-exist within a township. In this survey, we selected only wards from Yangon and only village tracts from Bago **to capture the features of urban and rural areas from each region;** we considered Yangon as representative of urban areas and Bago of rural areas.”

**Reviewer3-7 comment:**

Methods:

4. In line 93 “Differences between wards and village tracts involve the degree of urbanization” What does it mean?

**Our reply 3-7:**

Thank you for this question.

We have changed the sentence as follows.

**“The difference between a ward and village tract is the degree of urbanization.”**

**Reviewer3-8 comment:**

Methods:

5. Sample size and/or participation rate need to be mentioned

**Our reply 3-8:**

Thank you for this comment. We have added the followings.

**“In Yangon, the surveyors visited 1,083 older adults; 610 were at home. Ten were excluded due to refusing the survey (*n* = 6) or to severe dementia or being bedridden (*n* = 4); the response rate was 98.4% in Yangon. In Bago, surveyors visited 1044 older adults; 694 were at home. Ninety-four were excluded due to severe dementia or being bedridden, thus the response rate was 86.5% in Bago. In total, six hundred people each from the Yangon (222 men and 378 women) and Bago regions (261 men and 339 women) were surveyed.”**

**Reviewer3-9 comment:**

Methods:

6. How did you train the trained surveyors and how did you recruit the participants?

**Our reply 3-9:**

Thank you for this comment. We have added the followings.

**“We hired research staff from the Myanmar Perfect Research Company, a group that conducts epidemiological surveys in Myanmar. The interviewers were recruited from the company. Before the commencement of the actual survey, a two-day training course on the research protocol, administration of the questionnaire, and ethical concerns was conducted for the interviewers. ”**

**Reviewer3-10 comment:**

Methods:

7. Description of survey tools is rather vague, please clarify clearly. What is Japan Gerontological Evaluation Study and how did you used in your study.

**Our reply 3-10:**

Thank you for this comment. We have added the followings

**“A 14-page** structured questionnaire—**based on a questionnaire used the “Japan Gerontological Evaluation Study (JAGES)(34)”**—was developed for face-to-face interviews. **JAGES was established in 2010 as a nationwide, population-based prospective cohort study for older community-dwelling Japanese adults. The linguistic translation and validation process followed the “Linguistic Validation Manual for Health Outcome Assessments (35).” The questionnaire was first developed in English. After being translated into Burmese (dominant language), these were back translated to English to ensure clarity and consistency.////**

**A small pilot study was carried out before the actual survey for face validity in Urban Health Center, Dagon township, Yangon. Participants were the older adults, age of above 60 came to the out-patient clinic in the center. We recruited the 25 respondents who were gave consent to participate in the pilot study, during time of pilot data collection in June 2018. During the pilot study, the interviewers ensured sequence, flow, and clarity of the study. After the feedback from the interviewers, the questionnaire was revised accordingly. To avoid the question order bias, we have placed questions about depressive symptoms away from question about subjective socioeconomic status (see supporting information).”**

**Reviewer3-11 comment:**

Methods:

8. You described the exclusion criteria in the study tool in “line 104”. You should mention both inclusion and exclusion criteria under the study design and participants.

**Our reply 3-11:**

Thank you for this comment. We have added the following.

**“The inclusion criteria were individuals aged 60 or older who were residents of the selected ward or village tract.”**

**Reviewer3-12 comment:**

Methods:

9. In your dependent variable, why do you use GDS question as screening tool for symptoms of depression in elder population. Please specify short version of your GDS items and mentioned where this question validated (only in Japan or other countries).

**Our reply 3-12:**

Thank you for this comment. As mentioned in “Our reply1-6, 2-4, 2-8, 3-10”, we have added the process of linguistic translation and validation as follows. We have also added reference which mention GDS validation and the questionnaire as “supporting information”.

**“A small pilot study was carried out before the actual survey for face validity in Urban Health Center, Dagon township, Yangon. Participants were the older adults, age of above 60 came to the out-patient clinic in the center. We recruited the 25 respondents who were gave consent to participate in the pilot study, during time of pilot data collection in June 2018. During the pilot study, the interviewers ensured sequence, flow, and clarity of the study. After the feedback from the interviewers, the questionnaire was revised accordingly. To avoid the question order bias, we have placed questions about depressive symptoms away from question about subjective socioeconomic status (see supporting information).”**

**“**We assessed depressive symptoms using the 15-item version of the Geriatric Depression Scale (GDS), which was validated previously (38-41). The GDS involves a simple yes/no format **(see supporting information Q17 1)-15)) that is easy to administer and score** (42, 43). **Participants were classified into two groups: no depressive symptom (GDS score < 5), and having depressive symptoms (GDS score ≥ 5) (39, 44-46).”**

**“40. Zalsman G, Aizenberg D, Sigler M, Nahshoni E, Weizman A. Geriatric depression scale-short form–validity and reliability of the Hebrew version. Clinical Gerontologist. 1998;18(3):3-9.**

**41. Sheikh JI, Yesavage JA. Geriatric Depression Scale (GDS): recent evidence and development of a shorter version. Clinical Gerontologist: The Journal of Aging and Mental Health. 1986.”**

**Reviewer3-13 comment:**

Methods:

10. In independent variable, please a brief specify wealth index indicators.

**Our reply 3-13:**

Thank you for this comment. We have added the followings.

**“**The wealth index used as an objective SES was calculated from household asset items using a method described in a previous report (47). **Principal component analysis was performed on the asset items (e.g., radio, black & white television, color television, Video/DVD player, electric fan, refrigerator, computer, store-bought furniture, personal music player, washing machine, gas cooker, electric cooker or rice cooker, air conditioner, bicycle, motorcycle, van/truck, microwave oven, mobile telephone, and internet), and the principal component score was calculated based on the participants’ possession of each item and used as the wealth index.”**

**Reviewer3-14 comment:**

Methods:

11. Regarding confounding variable, how to do you ask social support question. Please specify this question.

**Our reply 3-14:**

Thank you for this comment. We have added the followings.

**“Social support was assessed by asking four questions. “Do you listen to someone else’s concerns and complaints (giving socioemotional support)?” “Do you take care of someone who is sick (giving instrumental social support)?” “Do you have someone who listens to your concerns and complaints (receiving emotional social support)?” “Do you have someone who takes care of you when you are sick (receiving instrumental social support)?” For these questions, the possible responses were 1. none, 2. spouse, 3. children living with them, 4. children or relatives living apart, 5. neighbor, 6. friend, and 7. other. Based on their responses, participants were categorized as “having no social support” (i.e. answering with ‘none’) or “having social support” (answering with any of choices 2 to 7) (48).”**

**Reviewer3-15 comment:**

Discussion

1. While discussing prevalence of depressive symptoms, there has been recently published prevalence of depressive symptoms in elder Myanmar population and you should also use it as reference.

**Our reply 3-15:**

Thank you for this comment. We have added the followings sentences and a reference.

“Previous research on community-dwelling older adults in Myanmar indicated that 22.2% of them appeared to have depressive symptoms (25) **and that about 16% to 56% of older adults in Myanmar have indications of depressive symptoms (26); the 22.1% found in the present study falls within this range.** The median prevalence rate of depressive symptoms for adults aged 60 years and above in the world is estimated to be 10.3% (49). **Although we cannot directly compare the prevalence in this study with that of previous studies because of differences in the study period, the measure of depressive symptoms used, and the inclusion and exclusion criteria for the study population,** the prevalence in Myanmar was relatively higher than the global prevalence.”

**“26. Yamada H, Yoshikawa K, Matsushima M. Geriatric Depressive Symptoms in Myanmar: Incidence and Associated Factors. Journal of Applied Gerontology. 2019:0733464819879605.”**

**Reviewer3-16 comment:**

Discussion

2. While appreciating your work, Have your screening instrument been standardized in Myanmar? If not, please talk about it in limitation.

**Our reply 3-16:**

Thank you for this comment. We have added the process of validation in Method and also added it in limitation.

**Method**

**A 14-page** structured questionnaire—**based on a questionnaire used the “Japan Gerontological Evaluation Study (JAGES)(34)”—**was developed for face-to-face interviews. **JAGES was established in 2010 as a nationwide, population-based prospective cohort study for older community-dwelling Japanese adults. The linguistic translation and validation process followed the “Linguistic Validation Manual for Health Outcome Assessments (35).” The questionnaire was first developed in English. After being translated into Burmese (dominant language), these were back translated to English to ensure clarity and consistency.** **///**

**A small pilot study was carried out before the actual survey for face validity in Urban Health Center, Dagon township, Yangon. Participants were the older adults, age of above 60 came to the out-patient clinic in the center. We recruited the 25 respondents who were gave consent to participate in the pilot study, during time of pilot data collection in June 2018. During the pilot study, the interviewers ensured sequence, flow, and clarity of the study. After the feedback from the interviewers, the questionnaire was revised accordingly. To avoid the question order bias, we have placed questions about depressive symptoms away from question about subjective socioeconomic status (see supporting information).”**

**Discussion**

**Finally, we developed the questionnaire in the dominant language based on the “Linguistic Validation Manual for Health Outcome Assessment (35).” However, it is necessary to improve the accuracy of the questionnaire.**

**Reviewer3-17 comment:**

Discussion

3. More cogent and clear arguments need to be made as to why low subjective SES is associated with depressive symptoms in older adults.

**Our reply 3-17:**

Thank you for this comment. We have revised and added the argument as follows.

**“This may be due to several factors. One reason may be that health disparities due to differences in subjective SES may increase depressive symptoms in older adults in Myanmar. Persistent inequalities exist in health outcomes in Myanmar's seven states and seven regions (52). Although a new National Health Plan (2017-2021) was launched in Myanmar, resource allocation does not seem to be closely aligned with the goal of reducing health disparities (52, 53). Even in Japan, considered an egalitarian society—as reflected by a Gini coefficient of 63% in 2019 (54)—with relatively few inequalities in health (55), substantial social inequalities in mental health, measured by SSS, were identified (11). In a previous systematic review and meta-analysis, there also appeared to be a consistent and statistically significant increase in the odds of coronary artery disease, hypertension, diabetes, and dyslipidemia when comparing low and high SSS (13). Although we adjusted for illness during the preceding year, low subjective SES may affect physical health due to the perception of status differentiation, which could lead to the risk of having depressive symptoms.**

**Another reason may be related to negative psychological consequences through stress-related psychological pathways due to low subjective SES. Research has often identified stress-related neuroendocrine processes as mechanisms underlying the association between perceptions of relative deprivation and poor health (56, 57). This idea was applied to a model of the relationships between objective SES, SSS, and obesity; the study suggests that SSS mediates the relationship between low SES and obesity through the activation of the hypothalamic–pituitary–adrenal (HPA) axis, a part of the neuroendocrine system controlling responses to stress (58). This idea is supported by empirical evidence showing that low SSS is associated with higher physiological stress markers (57, 59, 60). Similarly, a neuroendocrine pathway may link subjective SES to depressive symptoms since evidence suggests that the stress-related dysregulation of the HPA axis also predicts the onset and recurrence of depression (61, 62).**

**An alternative explanation of why low subjective SES is associated with depressive symptoms may be related to the difference between the extent to which the subjective SES and objective SES can be captured. One study indicates that SSS might not only represent a cognitive average of current socioeconomic circumstances, but also take account of past trajectories and perceived future prospects (63). Additionally, when people rank themselves on the SSS ladder, they might refer to socioeconomic factors other than (or additional to) objective SES (62, 64). Similar to the SSS ladder, subjective SES may be a comprehensive measure of people’s socioeconomic situation, beyond the traditional objective indicators of SES.”**

**Reviewer3-18 comment:**

Discussion

4. I am not sure as to why some of the results were presented in the Discussion Section (line 211, 214, 215, 216)

**Our reply 3-18:**

Thank you for this comment.

We have deleted the results which were presented in the Discussion Section.

**Reviewer3-19 comment:**

Discussion

5. I found the arguments presented in the Discussion section rather disjointed. There needs to be more flow of discussion that tells a clear story. Some of the arguments are lacking in substance and/or details- for example, in line 216 to 219.There needs to be a clear discussion.

**Our reply 3-19:**

Thank you for this comment. We have revised and added the argument as showing in “our reply 3-17”.

**Reviewer3-20 comment:**

Conclusion

Clarification and more meaningful detail/interpretation required.

**Our reply 3-20:**

Thank you for this comment. We have added implication and revised conclusion as follows.

**“Intervention programs for depression in older adults, which include social protection, sustainable livelihood, and wealth creation, should consider regional differences in the context of subjective SES by reducing economic disparities between rural and urban areas and within communities. Religiousness must also be considered.**

**In conclusion, the influence of subjective SES on depressive symptoms may have been greater than that of objective SES in the two urban and rural areas in Myanmar,** especially in the rural area. **Approaches to subjective SES rather than material wealth are considered more important for depression in older adults in the area.”**

”

**Reviewer 4 comments:**

**Reviewer4-1 comment:**

Abstract

1. Methods: I suggest including the number of participants in the first sentence of this section.

**Our reply 4-1:**

Thank you for this comment. We have revised the sentence as follows.

**“**Multistage random sampling and face-to-face interviews were conducted in two regions in Myanmar, **and 1,200 participants, aged 60 years and above, were included in the database.”**

**Reviewer4-2 comment:**

Abstract

2. Methods: what is the “cutoff of five” referring to in the sentence regarding the Geriatric Depression Scale?

**Our reply 4-2:**

Thank you for this comment. We have added the sentence as follows.

“The Geriatric Depression Scale (GDS) was used to evaluate the depressive symptoms. **Participants were classified into two groups: no depressive symptom (GDS score <5) and having depressive symptoms (GDS score ≥5).”**

**Reviewer4-3 comment:**

Introduction

1. What is meant by objective and subjective socioeconomic status? Authors need to provide definition to these concepts, as well as contrasts between the two concepts.

**Our reply 4-3:**

Thank you for this comment. We have added the sentence as follows.

“Research supports the conclusion that low objective socioeconomic status (SES)— **defined as the economic and social position in relation to others, such as working status, household wealth, and poverty status (1-4)—**is correlated with poor health in older adults (4-8).”

“It is also suggested that subjective SES— **a person’s conception of his or her position compared with that of others (1-3) (e.g.,** perceptions of having a lower/higher SES, **which is determined by whether respondents rated themselves as lower than middle class in the country or the community; lower ratings suggested a lower SES),** is of importance (11-13).”

**Reviewer4-4 comment:**

Introduction

2. Line 63: Need to clarify how the perception of lower/higher SES is determined. Are individuals self-reporting? Are they asked to compare their status to others? Please expand the discussion of this concept.

**Our reply 4-4:**

Thank you for this comment. We have added the followings as showing in “Our reply 4-3”.

“It is also suggested that subjective SES— **a person’s conception of his or her position compared with that of others (1-3) (e.g.,** perceptions of having a lower/higher SES, **which is determined by whether respondents rated themselves as lower than middle class in the country or the community; lower ratings suggested a lower SES),** is of importance (11-13).”.

**Reviewer4-5 comment:**

Introduction

3. Line 74: What is meant by the “socioeconomic cost of depression”? Please expand discussion.

**Our reply 4-5:**

Thank you for this comment. We have added the followings.

“However, there are few studies regarding the effects of objective/subjective SES on depressive symptoms among older adults in developing countries **including Myanmar.** However, depressive symptoms are common among older adults as the aging population grows, especially in Asian countries (21), and its socioeconomic cost is vast due to **higher rates of morbidity and mortality and increased healthcare utilization and economic cost compared to younger adults (22).”**

**Reviewer4-6 comment:**

Introduction

4. Line 77: Needs clarity in writing. Authors should present the “doubling” statistic, or the 25% increase statistic. Both are not needed.

**Our reply 4-6:**

Thank you for this comment. We have revise the sentence as follows.

“**In Myanmar, the proportion of the population aged ≥ 60 years is anticipated to increase from about 10% in 2020 to nearly 18.5% by 2050 (23).**”

**Reviewer4-7 comment:**

Introduction

5. Line 80: Unclear what authors are examining in the study. Are you interested in both objective AND subjective SES? Examining in the same models? In separate models? Please be precise.

**Our reply 4-7:**

Thank for this comment. We have added the followings.

“This study aimed to examine whether objective/subjective SES, **which are examined in the same model**, are associated with depressive symptoms in older adults **in urban areas of Yangon and rural areas of Bago** in Myanmar.”

“Variables for objective and subjective SES and the other variables with an associated p-value level less than 0.05 **in the bivariate analysis** were entered **into the same model using multiple logistic regression.**”

**Reviewer4-8 comment:**

Methods

1. Line 85: Include how many participants are in the study up front.

**Our reply 4-8:**

Thank you for this comment. As “our reply 3-4, we have revised the sentence as follows.

“This was **a cross-sectional baseline survey** for a longitudinal study. It **was conducted between September and December 2018 in Myanmar** and examined the predictors of physical and psychological health in **1,200** community-dwelling older adults aged ≥ 60 years.

”

**Reviewer4-9 comment:**

Methods

2. Line 114: Please provide a more in depth explanation of the objective SES measure.

**Our reply 4-9:**

Thank you for this comment. As “our reply 3-13”, we have added a sentence as follows.

“The wealth index used as an objective SES was calculated from household asset items using a method described in a previous report (47). **Principal component analysis was performed on the asset items (e.g., radio, black & white television, color television, Video/DVD player, electric fan, refrigerator, computer, store-bought furniture, personal music player, washing machine, gas cooker, electric cooker or rice cooker, air conditioner, bicycle, motorcycle, van/truck, microwave oven, mobile telephone, and internet), and the principal component score was calculated based on the participants’ possession of each item and used as the wealth index.”**

**Reviewer4-10 comment:**

Methods

3. Line 115: Better describe the subjective SES measure. What does “average” mean in the possible responses? Average compared to who? What is “very difficult” or “very comfortable” supposed to mean?

**Our reply 4-10:**

Thank you for this comment. The response is based on their perception of their current economic situation in light of general economic conditions. We have described it more clearly as follows.

“Subjective SES was assessed by asking: “Which of the following best describes your current financial situation in light of general economic conditions?” **The participants were asked to select from five options; their perception of their current financial situation was 1. very difficult, 2. difficult, 3. average, 4. comfortable, and 5. very comfortable.** Based on their responses, participants were categorized as having “average or more” (answering 3, 4, or 5) or “difficult or very difficult” (answering 1 or 2) perceived SES **considering general economic conditions.”**

**Reviewer4-11 comment:**

Methods

4. Line 127: Please better describe the social support variable. What is emotional vs instrumental help? Are these different variables? Or are they all included into 1 variable?

**Our reply 4-11:**

Thank you for this comment. As “our reply 3-14”, we have added followings.

**“Social support was assessed by asking four questions. “Do you listen to someone else’s concerns and complaints (giving socioemotional support)?” “Do you take care of someone who is sick (giving instrumental social support)?” “Do you have someone who listens to your concerns and complaints (receiving emotional social support)?” “Do you have someone who takes care of you when you are sick (receiving instrumental social support)?” For these questions, the possible responses were 1. none, 2. spouse, 3. children living with them, 4. children or relatives living apart, 5. neighbor, 6. friend, and 7. other. Based on their responses, participants were categorized as “having no social support” (i.e. answering with ‘none’) or “having social support” (answering with any of choices 2 to 7) (48).”**

**Reviewer4-12 comment:**

Methods

5. Line 135: Were all variables included in 1 model? Was there a model for objective SES and a different model for subjective SES? This needs to be better described.

**Our reply 4-12:**

Thank you for this comment. As “our reply 4-7”, we have added followings.

“This study aimed to examine whether objective/subjective SES, **which are examined in the same model,** are associated with depressive symptoms in older adults **in urban areas of Yangon and rural areas of Bago** in Myanmar.”

“Variables for objective and subjective SES and the other variables with an associated p-value level less than 0.05 **in the bivariate analysis** were entered **into the same model using multiple logistic regression.**”

**Reviewer4-13comment:**

Methods

6. Line 136: When and how was statistical significance assessed for the covariates?

**Our reply 4-13:**

Thank you for this comment. As “our reply 4-7 & 4-12”, we have added followings.

“Variables for objective and subjective SES and the other variables with an associated p-value level less than 0.05 **in the bivariate analysis** were entered **into the same model using multiple logistic regression.**”

**Reviewer4-14 comment:**

Results

1. Line 158: among those who did not what?

2. Line 162: among those who did not what?

**Our reply 4-14:**

Thank you for this comment. We have revised the sentences as follows.

“The above sociodemographic characteristics were compared between participants who had depressive symptoms (GDS score ≥ 5) and those who did not **have depressive symptoms** (GDS score <5) using Pearson’s chi-square test.”

“The rates of both low objective and subjective SES were significantly higher among respondents who had depressive symptoms (51.7% and 43.8%, respectively) than those who did not **have depressive symptoms** (36.3% and 13.9%, respectively).”

**“Both experiences of illness during the preceding year and no school** were significantly higher among those who had depressive symptoms (64.9% and **10.6%, respectively**) than those who did not **have depressive symptoms** (47.3% and **8.0%, respectively).”**

“Although most participants had social support (giving and receiving emotional and instrumental help), the rate of instrumental support received was significantly lower among those who had depressive symptoms compared with those who did not **have depressive symptoms** (95.1% and 98.6%, respectively).”

“Nearly half of the respondents visited religious facilities once a week or more (48.6%), and the rate was significantly higher among those who did not have depressive symptoms than those who **had depressive symptoms** (50.7% and 42.3%, respectively).”

**Reviewer4-15 comment:**

Results

3. Line 163: What do authors mean by “significantly higher”? Clarify this.

**Our reply 4-15:**

Thank you for this comment. We have revised the sentence as follows.

“Most of the participants lived with others (94.3%), and **the number of participants who lived with others was significantly higher among those who did not have depressive symptoms (96.0%) than those who had depressive symptoms (88.7%).”**

**Reviewer4-16 comment:**

Results

4. Line 166: Need to clarify what is meant by emotional vs instrumental support

**Our reply 4-16:**

Thank you for this comment. As “our reply 3-14”& “4-11”, we have added followings in the Method section.

**“Social support was assessed by asking four questions. “Do you listen to someone else’s concerns and complaints (giving socioemotional support)?” “Do you take care of someone who is sick (giving instrumental social support)?” “Do you have someone who listens to your concerns and complaints (receiving emotional social support)?” “Do you have someone who takes care of you when you are sick (receiving instrumental social support)?” For these questions, the possible responses were 1. none, 2. spouse, 3. children living with them, 4. children or relatives living apart, 5. neighbor, 6. friend, and 7. other. Based on their responses, participants were categorized as “having no social support” (i.e. answering with ‘none’) or “having social support” (answering with any of choices 2 to 7) (48).”**

**Reviewer4-17 comment:**

Results

5. Line 180: report AOR and CI for objective SES

6. Line 182: report AOR and CI for objective SES

**Our reply 4-17:**

Thank you for this comment. We have added AOR and CI for objective SES.

“Even after adjusting for these confounding variables, low subjective SES was positively associated with depressive symptoms **(AOR: 4.18, 95% CI: 2.98–5.87),** but low objective SES was not significantly associated **(AOR: 0.90, 95% CI: 0.61–1.32)** (Table 2).”

“Low subjective SES was still significant after being stratified by region—Yangon (AOR: 2.10, 95% CI: 1.08–4.05) and **Bago (AOR: 5.65, 95% CI: 3.69–8.64)**—, but low objective SES was not **(Yangon, AOR: 1.65, 95% CI: 0.80–3.41; Bago, AOR: 0.74, 95% CI: 0.47–1.17)** (Table 2a).”

**Reviewer4-18 comment:**

Results

7. Line 183: Authors just presented regional stratification. No need to mention here.

**Our reply 4-18:**

Thank you for this comment. We have deleted the sentence.

**Reviewer4-19 comment:**

Results

8. Line 185: Need clarity as to why the authors are only reporting results for the Bago region. This needs to be clarified in the methods section.

**Our reply 4-19:**

Thank you for this comment. We have added the followings.

“Among men and women in Yangon, both low objective and subjective SES were not associated with depressive symptoms **(objective SES for urban men, AOR: 1.27, 95% CI: 0.22–7.52; subjective SES for urban men, AOR: 2.84, 95% CI: 0.74–10.86; objective SES for urban women, AOR: 1.88, 95% CI: 0.82–4.27; subjective SES for urban women, AOR: 1.91, 95% CI: 0.88–4.14). Meanwhile receiving instrumental support and the frequency of visits to religious facilities once per week or more were both negatively associated with depressive symptoms in urban men (receiving instrumental support for urban men, AOR: 0.05, 95% CI: 0.01–0.38; frequency of visits to religious facilities for urban men, AOR: 0.23, 95% CI: 0.05–0.96), but not in urban women (receiving instrumental support for urban women, AOR: 0.12, 95% CI: 0.01–1.19; frequency of visits to religious facilities for urban women, AOR: 1.02, 95% CI: 0.60–1.76)** (Table 2a). Among residents of Bago, low subjective SES was positively associated with depressive symptoms among both men and women **(rural men: AOR: 8.97, 95% CI: 4.46–18.07; rural women: AOR: 4.45, 95% CI: 2.57–7.72),** while the frequency of visits to religious facilities once per week or more was negatively associated, respectively **(rural men: AOR: 0.45, 95% CI: 0.23–0.87; rural women: AOR: 0.39, 95% CI: 0.23–0.67)** (Table 2b).”

**Reviewer4-20 comment:**

Discussion

1. Line 222: Authors need to describe how SSS compares to SES and why this is applicable.

**Our reply 4-20:**

Thank you for this comment. We have added the following in the Introduction section.

**“Additionally, subjective social status (SSS), which is defined as a person’s perceived rank relative to others in their group, and concept similar to subjective SES (15) have been found to mediate the associations between objective SES and depression (16)”.**

**Reviewer4-21 comment:**

Discussion

2. Line 238: Authors need to better link their results to this discussion of the potentially unique aspects of rural life. Also, can authors look at the rural vs urban differences in social support and tie that into this section? Social support may be a good proxy for social capital. Further explore this variable in a post-hoc analysis and greatly expand this discussion.

**Our reply 4-21:**

Thank you for this comment. As in the comment of Reviewer2-7 (Lines 238–Bonding social capital was not directly questioned in this research. This should be mentioned in the limitation section.), we have moved this section to the limitation section as follows. The point you have suggested is of utmost importance. In the future, we would like to analyze these points and author a paper focusing on rural and urban differences in social support.

**“Fifth, indicators for bonding social capital, which is derived from relationships between similar persons such as with respect to sociodemographic and socioeconomic characteristics, were not included in our analysis./**// This may also increase the risk of depressive symptoms in rural older adults. Although we adjusted for instrumental social support, which was associated with depressive symptoms in a bivariate analysis, **further studies are needed to examine the association between types of social support and human interactions and depressive symptoms in Myanmar.”**

**Reviewer4-22 comment:**

Table 1.

1. What does the “Missing” column add to the table? This data can be noted elsewhere on the table and save space.

**Our reply 4-22:**

Thank you for this suggestion. We have deleted the “Missing” column, and noted it on the Table foot (Please see Table 1)

**Reviewer4-23 comment:**

Table 2.

Tables 2a-2b

Tables 2c-2f

1. Add more space between the rows. This will make it easier to read.

**Our reply 4-23:**

Thank you for this suggestion. We add more space between the rows (Please see Tables 2, 2a-2b). We combined some Tables based on your comment 4-26& 4-27.

**Reviewer4-24 comment:**

Table 2.

Table 2a-2b

Tables 2c-2f

2. Why is Monastic education tied in with No School? Should these be 2 different categories?

**Our reply 4-24:**

Thank you for this comment. We have divided it into two categories and reanalyzed the data. (Please see the Results Section and Tables 1, 2a, and 2b).

**Reviewer4-25 comment:**

Table 2.

Tables 2a-2b

Tables 2c-2f

3. Why are widowed and divorced the same category? Could they have different effects on depression?

**Our reply 4-25:**

Thank you for this question. We wanted to know the present marital status and the difference between those married and not. Additionally, the number of widowed and divorced individuals were small; it was difficult to analyze the data statistically by categorizing them.

**Reviewer4-26 comment:**

Tables 2a-2b

4. Include the rural and urban results on the same table, with the AOR and CI next to one another. This will make it easier to compare. I don’t need to see the SE or the z statistic.

**Our reply 4-26:**

Thank you for this suggestion. We have combined Table 2a with Table 2b (Please see Table 2a)

**Reviewer4-27 comment:**

Tables 2c-2f

4. Include the rural and urban results for men and women on the same table, with the AOR and CI next to one another. This will make it easier to compare. I don’t need to see the SE or the z statistic.

**Our reply 4-27:**

Thank you for this suggestion. We have included the rural and urban results for men and women on the same table (please see Table 2b)
