# Supplementary material for: Association between depressive symptoms and objective/subjective socioeconomic status among older adults of two regions in Myanmar
Source: PLoS One. 2021 Jan 28;16(1):e0245489. doi: 10.1371/journal.pone.0245489 (PMC7842968; doi:10.1371/journal.pone.0245489)
Supplement: Attachment — Submitted filename: Response to Reviewers(R2).docx [file pone.0245489.s004.docx]

Response to Reviewers

Re: PONE-D-20-15915(R2)

Association between depressive symptoms and objective/subjective socioeconomic status among older adults of two regions in Myanmar

Response to reviewers

We wish to thank the reviewers for giving us helpful comments regarding our manuscript. We have revised our manuscript accordingly.

Reviewer comment 1-1:

Having expanded the discussion of existing literature in the Introduction, it would be good to reflect back on this and demonstrate where the studies findings agree or disagree with that literature. Did you find anything novel, and are there new research questions that we now need to address?

Our reply 1-1

We thank the reviewer for this precious comment.

We have added the followings in Discussion

➡

**The result was not in accordance with studies in middle- or high-income countries that only investigated the association between objective SES and mental health (5, 6, 9). However, it was similar to the results of a previous meta-analysis showing that subjective SES affects physical health more than objective SES (19). As far as we know, this is the first study showing similar results for mental health. Moreover, our study also found that, after being stratified by region and sex, low subjective SES was still associated with depressive symptoms in rural older adults, but not in urban older adults.**

///

**A detailed study should also be conducted on how unique factors of the cultural background such as religiosity affect the mental health of older men and women in each region in Myanmar.**

Reviewer comment 1-2:

Lines 331-333, more information is needed to support the statements made in this sentence. What is it about the resource allocation that does not align with reducing health disparities?

Our reply 1-2

Thank you for this important comment. We have added the followings

**For example, according to Zaw et al. (52), conventional budget allocations related to population and infrastructure provide disproportionately more resources to regions with better health and less resources to some areas with high health needs.**

Reviewer comment 1-3:

Thank you for the explanation about the lack of impact in Myanmar from the 2018 recession. I think it would be helpful to add a sentence in the discussion to explain that the 2018 recession had minimal effect on Myanmar, just to help anyone considering the external validity of your findings and to demonstrate that this had been considered.

Our reply 1-3

Thank you for this comment.

We have added the followings in Discussion section.

➡

**In addition, for the external validity of our findings, we expect that the international recession that began in 2018 has not had a significant influence on our findings, since the Gross Domestic Product (GDP) in Myanmar was equivalent to US$ 76.17 billion in 2018 (80), and the distribution of household income also increased between 2000–2018: The proportion of the population in the middle-income group (household income of US$ 5,000–34,999) rose from about 1.2% in 2000 to 20.6% in 2018 (81).**

Reviewer comment 1-4:

Sentences were extra clarity is needed

Our reply 1-4.

Thank you for carefully reviewing our manuscript.

We have revised them and had native speakers of English proofread our English writing, accordingly.

We

• Lines 46-48

Interventions for depression in older adults should consider regional differences in the context of their subjective SES by reducing disparities among the community in Myanmar.

➡

**Interventions for depression in older adults should take regional differences into consideration in the context of subjective SES by reducing socio-economic disparities among the communities of Myanmar.**

• Line 56, I wonder if describing objective SES using the term ‘in relation to others’ could be confusing.

Thank you for this comment. We have deleted ‘in relation to others’ as follows.

—defined as the economic and social position in relation to others, such as working status, household wealth, and poverty status (1-4)—is///

➡

**Objective socioeconomic status (SES) is defined as the economic and social position such as working status, household wealth, and poverty status (1-4).**

• Line 77, I wonder if it should be ‘concepts’ plural rather than ‘concept’ singular.

Thank you for this comment. We have revised it based on your comment.

///which is defined as a person’s perceived rank relative to others in their group, and concept similar to subjective SES///

➡

///which is defined as a person’s perceived rank relative to others in their group, and **concepts** similar to subjective SES///

• Lines 85-86

Therefore, both objective and subjective SES are hypothesized to be one of the most significant disparities, where physical and psychological health are poor among individuals of lower SES.

➡

**Therefore, both objective and subjective SES are hypothesized to indicate the most significant disparities, such that individuals of lower SES tend to have physically and psychologically poor health.**

• Lines 93-96 could be broken into two sentences to be clearer.

Thank you for this comment. We have revised it as follows.

However, depressive symptoms are common among older adults as the aging population grows, especially in Asian countries (21), and its socioeconomic cost is vast due to higher rates of morbidity and mortality and increased healthcare utilization and economic cost compared to younger adults (22).

➡

However, depressive symptoms are common among older adults as the aging population grows, especially in Asian countries **(21).The** socioeconomic cost for older adults is vast due to higher rates of morbidity and mortality and increased healthcare utilization and economic cost compared to younger adults (22).

• Line 98 needs more detail, explanation and a reference

Thank you for this comment. We have described it in detail and added references.

The status of depression is also expected to be severe. ➡

The status of depression is also expected to be severe **(24). Depression and anxiety account for five per cent of disability-adjusted life years, which puts them in the top 10 contributors to disability in Myanmar (24, 25). However, there is no policy on mental health in Myanmar. As a result, mental health services have not received priority in primary healthcare, preventing thousands of people from accessing the mental health services they need (24).**

• Line 102, it should be fewer activities of daily living. Is the point that people undertake fewer activities or that they struggle with them more?

Thank you for this comment. We described it clearly as follows.

Studies in Myanmar have found that depressive symptoms in older adults were strongly associated with less activities of daily living and lower quality of life (26) and economic and health status (27).

➡

Studies in Myanmar have found that depressive symptoms in older adults were strongly associated **with less independence in seven items (walking, ascending and descending stairs, feeding, dressing, going to the toilet, bathing, and grooming)** and lower quality of life (26) and economic and health status (27)

• Lines 107-109 I think this sentence introduced a new concept that is not sufficiently explained, so the sentence can be deleted.

Thank you for this comment. We have deleted the sentence below.

Certain studies focusing on the effects of Vipassana mediation indicated that participants in the mediation courses reported reductions in anxiety and stress (29, 30).

• Line 113, I am not sure of the meaning of ‘Myanmar’s ethic areas’, could this be phrased differently to make more sense to people outside of Myanmar?

Thank you for this question. We have intended to refer to the internal conflicts. We have revised the sentence as follows.

///and ongoing conflicts in Myanmar’s ethnic areas (30-32).

➡

**/// and the ongoing internal conflicts in some states of Myanmar (30-32).**

• Line 148, 1,044 as you have used the comma separator elsewhere.

Thank you for this comment. We have revised it.

In Bago, surveyors visited 1044 older adults///

➡

In Bago, surveyors visited **1,044** older adults///

• Lines 169-170, I think ‘who’ needs to be added before came to make the meaning clearer.

Thank you for this comment. We have added ‘who’.

Participants were the older adults, age of above 60 came to the out-patient clinic in the center.

**➡**

Participants were the older adults, age of above 60 **who** came to the out-patient clinic in the center.

• Line 232, the term multivariate implies that you were using models that predicted multiple dependent variables, where I think you want ‘multivariable’ which means that there were multiple independent variables being used to predict a single dependent variable.

Thank you for this comment. We have revised it as follows.

Variables for objective and subjective SES and the other variables with an associated p-value level less than 0.05 in the bivariate analysis were entered into the same model using multiple logistic regression.

**➡**

Variables for objective and subjective SES and the other variables with an associated p-value level less than 0.05 in the bivariate analysis were entered into the same model using **multivariable** logistic regression. The **multivariable** adjusted results were expressed as adjusted odds ratios (AOR) with 95% confidence intervals (CI).

• Line 234, add a citation for Stata

Thank you for this comment. We have added a citation.

We used STATA14 to perform all statistical analyses

➡

We used STATA14 to perform all statistical analyses **(48)**

• Lines 285-286, I think it is best to say that they were not ‘significantly’ associated, as the lack of statistical significance does not imply the lack of association, just that you cannot accept the alternative hypothesis.

Thank you for this comment. We have revised it as follows.

Among men and women in Yangon, both low objective and subjective SES were not associated with depressive symptoms

➡

Among men and women in Yangon, both low objective and subjective SES were not **significantly** associated with depressive symptoms

• Lines 307-308, the beginning of this sentence is not clear.

Thank you for this comment. We have revised the sentence as follows.

The model with potential confounding factors was adjusted, suggesting that older adults with low subjective SES were more likely to experience depressive symptoms relative to those with average or more subjective SES, ///

➡

**Those with low subjective SES were more likely to experience depressive symptoms than those with average or higher subjective SES, even after adjusting for potential confounding factors.**

• Line 393, I would say you undertook a ‘large number of statistical tests for the sample size’.

Thank you for this comment. We have revised it as follows.

Fourth, there were several statistical tests undertaken for the sample size///

**➡**

Fourth, there **was a large number of statistical tests** for the sample size **///**

• Line 417-418, why is it necessary？

Thank you for this comment. We have added the reasons as follows

However, it is necessary to improve the accuracy of the questionnaire.

➡

However, it is necessary to improve the accuracy of the questionnaire **to minimize such errors and obtain higher-quality results.**

Reviewer #2: I agree with the authors’ response and correction to my previous review comments.

Still, the authors should review their logical flow and expressions.

Reviewer comment 2-1

On page 2, the background section in the abstract, this paragraph starts from ‘This study examines....’ Here, the authors should start from this study’s background, then explain how the background made the authors launch this study.

Our reply 2-1.

Thank you for this valuable comment.

We have revised it as follows.

➡

**Background**

**Low objective SES (e.g., household wealth; working and poverty status) has been correlated with poor physical and mental health. Some suggest that subjective SES (perceptions of having lower SES) is also as important. However, no studies have been conducted on the impact of both objective and subjective SES on depressive symptoms in older adults in Myanmar. This study examines whether subjective or objective socioeconomic status (SES) is associated with depressive symptoms in older adults in Myanmar.**

Reviewer comment 2-2

On page 4, the introduction section, ‘Research’ should be ‘Previous research,’ and it looks strange to find the word ‘conclusion’ in the very first sentence of the paper. There are numerous better alternatives such as ‘Previous studies demonstrated ...,’ ‘...has been recognized...’, and so on.

A description of SES was also embedded into one sentence, making the sentence a little unreadable. The authors should explain SES first, then refer to the relation between SES and poor health in older adults.

Our reply 2-2.

Thank you for this important comment.

We have revised it based on your comment.

➡

**Objective socioeconomic status (SES) is defined as the economic and social position such as working status, household wealth, and poverty status (1-4).** Previous studies have been recognized that low objective SES is correlated with poor health in older adults (4-8).///

**Subjective SES is defined as** a person’s conception of his or her position compared with that of others (1-3) (e.g., perceptions of having a lower/higher SES, which is determined by whether respondents rated themselves as lower than middle class in the country or the community; lower ratings suggested a lower SES) (11-13).
