# Supplementary material for: Association between depressive symptoms and objective/subjective socioeconomic status among older adults of two regions in Myanmar
Source: PLoS One. 2021 Jan 28;16(1):e0245489. doi: 10.1371/journal.pone.0245489 (PMC7842968; doi:10.1371/journal.pone.0245489)
Supplement: Attachment — Submitted filename: renamed_cc23e.docx [file pone.0245489.s005.docx]

**Editor’s comments**

Thank you for your efforts in addressing our reviewers’ comments. The manuscript has been remarkably improved. However, before this manuscript can be published, the writing quality requires substantial improvement to ensure the manuscript's readability and accuracy. Below are some examples of incorrect sentences, long sentences, excessive paragraphs, grammatical errors, and typos that can be easily found throughout the text and need corrections. Please note that my comments are exhaustive. Please proofread the revised manuscript carefully before resubmission.

**Abstract**

1. Please ensure that the manuscript's style and formats are aligned with those of PLOS ONE, including spacing, capitalization, punctuations, etc.
2. Please ensure that all abbreviations/acronyms are spelled out at the first use (e.g., SES, line 21). The abbreviations should be used consistently afterward (e.g., SES, line 26).
3. Please use a comma, instead of semi-column, between words or phrases (e.g., line 21). This comment is applied throughout the manuscript.
4. Please ensure consistencies, e.g., socioeconomic (line 26) or socio-economic (line 49).
5. Background:

- First sentence: … has been correlated with poor physical and mental health. Among whom? Please state the populations specifically.

- Second sentence: Some suggest that…is also important. Some studies, some researchers or else? Important for what? Please specify.

- The last two sentences can also be improved. The authors stated that “no studies have been conducted on the impact of both objective and subjective SES on depressive symptoms in older adults in Myanmar.” Then the objective read, “…whether subjective or objective SES is associated with depressive symptoms in older adults in Myanmar.” The expression redundancy makes the reading quite uncomfortable.

6. Methods:

- Please revise the first sentence to read that you conducted the study using ‘a multistage random sampling method’ to recruit the participants and ‘face-to-face interviews’ to collect the data. The current statements mean you conducted the sampling methods and face-to-face interviews, which is not entirely correct. Please also provide essential information in this section (study period, design, and participants). Consider something like, “This cross-sectional study was conducted in… A multistage sampling method was used to recruit participants from two regions for face-to-face interviews.” No need to repeat ‘in Myanmar.”

Please summarize the variable measurements and keep space for briefly describing statistical methods.

Please move the number of participants and age to results.

7. The results section is too slim as compared to other sections:

- The authors may provide the number of participants included in the analyses with a mean age (or median) and SD (or IQR) and sex.

- Please also provide the proportion of participants with depressive symptoms.

- Were the results in the first sentence from bivariate analyses? If so, please state it clearly. Alternatively, the authors may present only multiple regression results in the abstract.

- Please provide statistical values to the result in the last sentence.

8. Conclusions:

- The term ‘influence’ is not appropriate to describe findings of cross-sectional studies that can only tell associations.

- Also, please specify your study participants in interpreting your results.

**Introduction**

1. The second sentence (lines 57-59): Previous studies have shown that… Also, when mentioning ‘health,’ please specify if it was ‘physical’ or ‘mental health.’
2. Line 59: It is also related to daily care needs (7) and long-term care needs (4, 5, 9).
3. Lines 65-68: The sentence is too long, particularly the part in parenthesis. The references (1-3) can be moved to join those at the end of the sentence. The authors may break the sentence into two.
4. The second (lines 65-89) and third (lines 90-116) are too long and difficult to follow. Please consider restructuring them.
5. Lines 111-113: Please justify how the country’s history of military control and internal conflicts are related to the lack of studies and publications on mental health among older adults.

**Methods**

1. Please change to ‘Materials and methods.’
2. Line 124: The target populations were those…
3. The information on lines 115-116 and 124-125 is somewhat overlapping.
4. The discussion on the representativeness of study samples (lines 126-128) is not in the right place and should be moved to the limitations section.
5. Line 129: A multistage random sampling method was used to select participants from the two regions.
6. Line 131:… using a probability proportional to size sampling method?
7. Line 134: What did ‘again’ mean?
8. Line 135: What did ‘extracted’ mean?
9. Line 136:…aged 60 years or older.
10. Line 150: In total, 600 people…
11. Line 154, 182, 189, 206: Please correct the subtitles, which are not understandable.
12. Line 155: …based on a questionnaire used in the…
13. Lines 160-162: Consider revise it as follows: “The questionnaire was developed in English, translated into Burmese, and back-translated to English to ensure clarity and consistency.”
14. Lines 163-164: “…a group with experiences in conducting epidemiological surveys in Myanmar.” The following sentence (The interviewers were recruited from the company) should be removed.
15. Line 168: Remove ‘small’ from ‘pilot study.’
16. Lines 169-170: Participants were older adults aged 60 years or older who came to the center's out-patient clinic.
17. Lines 170-17: We recruited 25 respondents who gave consent to participate in the pilot study in June 2018.
18. Line 173: …of the questionnaire?
19. Lines 177-180: Please move the inclusion criteria to the earlier section under the ‘Participants.’ Also, the second sentence should read, “We excluded individuals who were bed-ridden or had severe dementia.
20. Line 184: Please clarify if the scale was previously validated in Myanmar or other countries?
21. Line 191: a principal component analysis was performed…
22. Line 196: The principal component score was calculated…
23. Lines 199-200: The participants were asked to select from five options. Their perception of their current financial situation was…
24. Lines 206: Please consider better terminology to replace ‘Confounding variables.’ I don’t they are not all absolute confounders. ‘Sociodemographic characteristics?’
25. Line 209: What does ‘monastic’ mean?
26. Line 214: …The questions included (1) Do you listen to…; (2)…
27. Line 225: Change the subtitle to ‘Statistical analyses.’
28. Line 226: Sociodemographic characteristics were compared…
29. Line 231: …in bivariate analyses…
30. Line 232: …were simultaneously entered in a model. Also, please remove ‘using multivariable logistic regression.’
31. Please use consistent terminologies: ‘multiple’ ‘multivariable,’ or ‘multivariate’
32. Lines 232-233: Adjusted odds ratios (AOR) were presented with 95% confidence intervals (CI).
33. Further descriptions of data analyses are required to reflect the analyses actually conducted (e.g., stratified by region, sex, etc.). These were not understood until the reader goes through the results.

**Results**

1. Lines 259-262: The results of the living status are interesting. The authors reported that more than 90% of the participants lived with others. In several cultures, living with others refers to living people other than their family or relatives (negative). However, in the table, the authors indicated two categories of the living status – ‘alone’ and ‘not alone.’ I assumed that living with others in this context means living with family or relatives (positive, with more social support). This is confirmed by the comparative results showing that the proportion of participants who did not live alone was significantly higher in those who did not have depressive symptoms. I would suggest changing the term ‘living with others’ to ‘not living alone.’
2. Lines 173-278: The sentence is too long and hard to read. Consider revising it: “Depressive symptoms were positively associated with being female (AOR: 1.64, 95% CI: 1.15–2.34), experiencing illness during the preceding year (AOR: 1.92, 95% CI: 1.41–2.61), and living in Bago (AOR:1.62, 95% CI: 1.10–2.38). Depressive symptoms were negatively associated with receiving instrumental support (AOR: 0.31, 95% CI: 0.12–0.77) and frequency of visits to religious facilities once per week or more (AOR: 0.57, 95% CI: 0.42–0.77).”
3. Line 278: “Even after adjusting for these confounding variables,…” brings more confusion than help. In my understanding, the results in this section are from multiple regression (adjusted). Suggest removing this or stating this at the beginning of the paragraph. Lines 280-281: “…., but low objective SES was not significantly associated (AOR: 0.90, 95% CI: 0.61–1.32)” is incomplete. Suggest removing it as it is not statistically significant.
4. Lines 281-284: Like the above comment, please make the comparisons expressions complete; e.g., still significant, low objective SES was not.
5. Lines 285-289: Suggest removing all non-significant results from the text. If needed, the authors may summarize them in a sentence and refer the reader to the tables.
6. Line 289: The expression ‘Meanwhile’ sounds awkward in the sentence as the earlier results were not statistically significant, and the following were.
7. Lines 289-301: Please rewrite the sentences, breaking them into smaller and simpler sentences as they are complicated to read.

**Discussion**

1. Lines 310-311: The discussion needs further elaboration – what were the results differences?
2. Lines 312-313: How this study’s result was similar to the results of a previous meta-analysis showing that subjective SES affects physical health more than objective SES? Did this study also examine the association between subjective SES and physical health? Also, please clarify the settings of studies included in the meta-analysis – Lower- and middle-income or high-income countries?
3. Line 314: “… this is the first study showing similar results for mental health.” This sentence is not understandable – similar to what? Mental health and what?
4. Lines 314-316: Consider simplifying this sentence: “Moreover, we found that low subjective SES was associated with depressive symptoms in rural older adults, but not in urban older adults.”
5. Lines 318-319: Please clarify ‘…appeared to have depression’ and ‘have indications of depressive symptoms.’ Are these equivalent to ‘having depressive symptoms?’ If not, the prevalence rates shouldn’t be compared. The authors may have to go to the original scales or measures used in those studies.
6. Line 325: What is the global prevalence? Please cite the sources.
7. Line 328: As commented earlier, please be specific when using the terminologies. It may not be accurate to say ‘confounding factors’ when the authors based only on the available variables associated with the outcome variable in bivariate analyses. A confounding variable must be associated with both independent and dependent variables. Suggest simply say something like “other covariates in the multiple regression model.”
8. Lines 326-348: Please consider re-structuring the paragraph, which is too long and difficult to read. Do not discuss different results in one paragraph. For example, I cannot see the connection between the first and second paragraphs.
9. Lines 331-332: I cannot get what this sentence says, “However, the impact of low subjective SES was more than two times stronger than these variables.” How was ‘impact’ measured in a cross-sectional study? What are ‘these variables?’
10. Lines 332-348: The authors spent too much space discussing the country’s health disparities and inequitable resource allocation. This information is important; however, it does not help explain the direct relationships between SES and mental health. The literature on the pathways from health disparities and mental health may help.
11. Lines 352-356: Please consider refining the long sentence. Also, the authors should focus on the relationships between SES and mental health, not physical health. The mechanisms and pathways to the outcomes can be different.
12. Lines 378-380: I am not sure if this statement supports the finding of the relationship between religiosity and mental health. While religiosity was higher in older adults and people living in rural areas, this study also shows that depression was also high among them.
13. Lines 384-434: The study’s limitations section is excessive and presented in a giant paragraph, which is very difficult to follow. Please condense and restructure it, reducing at least 70% of the contents.
14. Line 384: Please clarify the data collection method and make it consistent across the paper – face-to-face or self-administered?
15. Lines 384-390: In the first limitation, the authors discussed two different issues – self-report measures and depressive symptoms measurement. These issues should not come along together.
16. Lines 391-397: I am not sure if the explanation on the disparities in health care access is really helpful, while this study looked at the relationship between SES and depressive symptoms.
17. Lines 403-422: The authors spent 19 lines to discuss one limitation. This can be reduced at least by 80%. This is also true for the final limitation (lines 422-434)
18. Lines 435-440: Not sure what the paragraph is about – limitations or recommendations? If it is the latter, it should be integrated with the conclusions.
19. Line 441-444: Overall, the conclusion is weak and hard to understand. The term ‘influence’ is not appropriate for describing cross-sectional associations. It’s also difficult to tell what the last sentence is about – was it based on this study or from other studies or the general perception (who considered it in that way?).
20. The authors may conclude the paper by clearly summarizing the key findings, followed by brief recommendations for policy, programs, or future studies.

**References**

Please revise the references list to be consistent and aligned with the journal’s guidelines.
